# Diversification Rate is Associated with Rate of Molecular Evolution in Ray-Finned Fish (Actinopterygii)

**DOI:** 10.1007/s00239-022-10052-6

**Published:** 2022-03-09

**Authors:** Andrew M. Ritchie, Xia Hua, Lindell Bromham

**Affiliations:** 1 Research School of Biological Sciences, Australian National University, Canberra, ACT 2600 Australia; 2 Mathematical Sciences Institute, Australian National University, Canberra, ACT 2600 Australia; 3 Research School of Biological Sciences, Australian National University, Robertson Building, 134 Linnaeus Way, Canberra, ACT 2600 Australia

**Keywords:** Sister pairs, Molecular rates, Diversification, Life history, Comparative methods, Phylogeny, Synonymous substitution rates

## Abstract

Understanding the factors that drive diversification of taxa across the tree of life is a key focus of macroevolutionary research. While the effects of life history, ecology, climate and geography on diversity have been studied for many taxa, the relationship between molecular evolution and diversification has received less attention. However, correlations between rates of molecular evolution and diversification rate have been detected in a range of taxa, including reptiles, plants and birds. A correlation between rates of molecular evolution and diversification rate is a prediction of several evolutionary theories, including the evolutionary speed hypothesis which links variation in mutation rates to differences in speciation rates. If it is widespread, such correlations could also have significant practical impacts, if they are not adequately accounted for in phylogenetic inference of evolutionary rates and timescales. Ray-finned fish (Actinopterygii) offer a prime target to test for this relationship due to their extreme variation in clade size suggesting a wide range of diversification rates. We employ both a sister-pairs approach and a whole-tree approach to test for correlations between substitution rate and net diversification. We also collect life history and ecological trait data and account for potential confounding factors including body size, latitude, max depth and reef association. We find evidence to support a relationship between diversification and synonymous rates of nuclear evolution across two published backbone phylogenies, as well as weak evidence for a relationship between mitochondrial nonsynonymous rates and diversification at the genus level.

**Supplementary Information:**

The online version contains supplementary material available at 10.1007/s00239-022-10052-6.

## Introduction

Variation in the tempo and mode of evolution is an important feature of the tree of life, which can potentially reveal evolutionary patterns and processes. At the largest and most obvious scales, the wide variation in diversity observed among higher-order taxa has been a target of evolutionary study for decades (Fischer 1960; May 1988; Currie 1991; Rohde 1992; Farrell 1998; Fine 2015). Although some of this pattern likely reflects differences in the amount of time lineages have to accumulate species (McPeek and Brown 2007; Hedges et al. 2015; Marin et al. 2018), it is clear that the net rate of species diversification varies quite significantly among taxa even after accounting for time (Cardillo 1999; Alfaro et al. 2009; Rabosky et al. 2012; Kozak and Wiens 2016; Scholl and Wiens 2016). At the other end of the scale, the microevolutionary process governing the rate of genomic evolution, driven by spontaneous mutation, segregation and fixation of molecular substitutions, is itself subject to variation on evolutionary timescales. Empirical patterns of molecular rate variation may be significantly correlated to variation in life history characters such as body size (Barrera-Redondo et al. 2018; Berv and Field 2018), lifespan (Galtier et al. 2009) or the rate of reproduction (Welch et al. 2008). These findings of consistent patterns of rate variation between lineages open the door to viewing the rate of molecular evolution as a trait that can itself evolve and diversify (Bromham 2009, 2011), subject both to selective pressures and random variation (Dobzhansky 1970; Lynch et al. 2016).

An increasing number of empirical studies explore the connection between microevolutionary change at the molecular level and macroevolutionary change at the level of biodiversity. In particular, over the past two decades, a pattern of correlation between the rate of molecular evolution and net diversification rate, as reflected in species richness of lineages, has been observed in several groups of organisms, including plants (Lancaster 2010; Bromham et al. 2015), birds (Lanfear et al. 2010a; Iglesias-Carrasco et al. 2019) and reptiles (Eo and DeWoody 2010). Similar relationships have been reported across a large range of phylogenies (Pagel et al. 2006). Several possible explanations have been given for the observation of a connection between substitution rates and species richness. The process of speciation may itself give rise to a period of accelerated molecular evolution, whether due to population structure, rapid adaptation, or rapid genomic divergence following from reproductive isolation (Barton and Charlesworth 1984; Venditti and Pagel 2010). Higher rates of molecular evolution may cause separated populations to achieve reproductive isolation more rapidly, increasing the chance that temporary separation leads to long-term incompatibility and speciation (Hua and Bromham 2017). Alternatively, diversification rates and rates of molecular evolution may be indirectly related if both are correlated with other variables. For example, climate and environment may be underlying drivers of variation in both molecular evolution and diversification (Barraclough and Savolainen 2001; Gillman and Wright 2014), or both diversification rate and mutation rate might be impacted by species traits such as sexual selection (Iglesias-Carrasco et al. 2019).

A relationship between molecular evolutionary rates and diversification rates is not only of theoretical interest but has direct implications for the relationship between observed molecular variation and the shape of molecular phylogenies. This could affect the inference of phylogenies, divergence times and macroevolutionary processes. Molecular rate variation can complicate the inference of diversification rates from phylogenies (Shafir et al. 2020), especially when there is a link between diversification and molecular rates (Duchêne et al. 2017). In Bayesian divergence time estimation, diversification (“tree”) models and rate variation (“clock”) models are typically specified independently (Bromham et al. 2018), and interactions between them are rarely considered (Sarver et al. 2019). While the impact of variation in rates of molecular evolution with diversification has been studied by simulations, the impact on empirical molecular dating and macroevolutionary studies is currently unknown because the extent of these relationships has not yet been characterised for most groups of organisms. It is, therefore, desirable to investigate how widespread the relationship between diversification rates and rates of molecular evolution is across the tree of life and evaluate the strength of the relationship.

Ray-finned fish (Actinopterygii) are the largest and most diverse major group of vertebrates, with currently over 35,000 described species (Fricke et al. 2022). The degree of diversity varies to a stunning extent among taxonomic groups, from the astounding richness of true gobies (Gobiidae, > 1900 species; McCraney et al. 2020) to the sparseness of the bowfin (Amiidae, 1 species) or sunfish lineages (Molidae, 5 species; Bass et al. 2005; Thompson et al. 2021; Fricke et al. 2022). This variability in diversity extends to biological characteristics: some groups show extreme morphological and ecological variation, such as the cichlids (Henning and Meyer 2014; Salzburger et al. 2014), while others show extreme morphological stasis, such as the paddlefish and bichir (Near et al. 2014; Cheng et al. 2021). It is particularly fascinating to see such diversity in marine or lake environments, because many of the mechanisms of terrestrial speciation, involving geographical barriers, are largely absent (Wiens 2015). Ecological gradients have been suggested to play a prominent role in driving speciation in fish (Schluter et al. 1996; Puebla 2009), and there are many accounts of such processes in specific species pairs, genera or families (e.g. Schliewen et al. 1994; Nagel and Schluter 1998; Rocha et al. 2008; Ingram 2011; May-McNally et al. 2015; Burress et al. 2017).

Other key features of fish diversity include the influence of coral reefs and coastal habitats as hotbeds of species richness and endemism (Cowman and Bellwood 2011; Parravicini et al. 2013; Cowman 2014) and the role of depth gradients in promoting both speciation and ecological divergence (Ingram 2011; Jennings et al. 2013; Ohlberger et al. 2013). An association between depth changes and speciation has been demonstrated in the speciose rockfish genus (Ingram 2011). Body size, usually measured as maximum specimen length, has been found to be negatively related to species richness (Fisher et al. 2010), but size has also been observed to be positively correlated with diversification rates in some reef fish, leading to uncertainty about the nature of the influence of size on speciation and extinction (Siqueira et al. 2020). The rate of body size evolution in fish is also positively correlated with speciation rates (Rabosky and Adams 2012). A latitudinal diversity gradient exists for both marine and freshwater fish species and may reflect factors such as temperature, primary productivity and ecological opportunity (Macpherson 2002; Tittensor et al. 2010; Chaudhary et al. 2016; Hanly et al. 2017), as well as time and historical glaciation cycles (Bernatchez and Wilson 1998; April et al. 2013; Miller and Román-Palacios 2021). However, despite the trend for reduced extant diversity with distance from the equator, rates of diversification have been shown to associate positively with latitude across the entire fish tree of life, potentially reflecting fish lienages that have radiated to fill new niches during their recolonisation of the higher latitudes following the last ice age (Rabosky et al. 2018).

The link between molecular rates and diversification rates has not received as much attention as the effects of geography or ecological traits. In addition to the practical interest, this correlation could have important implications for hypotheses of fish diversification. For instance, such a correlation would be consistent with a prediction of the evolutionary speed hypothesis, which suggests that temperature drives faster mutation rates and/or more rapid life history cycles (Rohde 1992; Gillman and Wright 2014). Several correlates of rate of molecular evolution have been identified in fish. Intraspecies genetic diversity has been related to surface temperature and region-specific factors (Manel et al. 2020) and surface temperature is also associated with differences in molecular rates between closely related species (Wright et al. 2011). Molecular rates have been shown to vary with ecological and life history characters, including body size, depth range and association with coral reefs, accounting for phylogenetic covariance across the fish tree of life (May et al. 2020). Body size in particular is frequently identified as a negative correlate of molecular rates (April et al. 2013; Hua et al. 2015; May et al. 2020). Since some of these correlates are also related to species richness or diversification rates, it is possible that molecular rates are related to diversification through life history or habitat, but this has not been thoroughly tested yet.

Rabosky et al. (2018) examined the relationship of tip-specific speciation rates, measured by the DR statistic (Jetz et al. 2012), and root-to-tip substitution path lengths, but the focus was on molecular rate as a confounding factor for the inverse latitudinal speciation gradient, and the relationship was not tested formally. Therefore, while some correlates of both diversification rates and rates of molecular evolution have been identified, it is not yet clear whether a relationship between molecular and diversification rates exists in ray-finned fish, either as an indirect effect of the relationship between life history and habitat with diversification and molecular rates, or as a direct connection between diversification and molecular evolution. This question is timely, as last decade has seen the coverage of the fish tree of life improve in leaps and bounds, providing more and more raw data to address this question (Near et al. 2012; Hughes et al. 2018; Rabosky et al. 2018). This provides the opportunity to determine whether ray-finned fish may be added to the growing number of taxa in which these scales of macro- and micro-evolution are related.

Common approaches for investigating relationships between molecular rates, diversification and life history or ecological traits can be divided into sister-pair analyses and tree-based analyses (Lanfear et al. 2010b). The method of sister-pair comparison is a robust way of exploring interactions between molecular evolution, life history and diversification (Garland et al. 1992; Freckleton 2000; Welch and Waxman 2008; Lanfear et al. 2010b; Bromham et al. 2015). In a sister-pair analysis, taxa are chosen that form pairs of non-overlapping sister clades, with each pair of sister clades sharing a common ancestor that is not included in any other sister comparison (Garland et al. 1992). Tree-based analyses instead infer substitutions for all branches in the phylogeny and correlate these with node numbers on root-to-tip paths in the tree (Webster et al. 2003).

Analysing correlates of diversification rates using sister clades has several important advantages. First, it satisfies the requirement of statistical independence because the differences in substitution rates and diversification rates between sister pairs are independent of any other such comparison, because they represent separate evolutionary histories. Second, because the two sister clades share an initial divergence date, the contrast in net diversification rates between the two clades can be estimated from the difference in number of extant species in these clades (clade sizes), and the contrast in molecular rates can be derived from the average number of substitutions occurring in each clade since divergence. The contrast in clade sizes can then be related to the contrast in inferred substitution rates using ordinary least squares regression. Calculating these contrasts does not require an knowledge of the absolute ages of these clades to serve as the denominator for calculating the rate, although an estimate of relative pair age, which may be based on molecular dates or the sum of branch lengths in the clade, can be used to correct for heteroscedasticity (Garland et al. 1992; Hua et al. 2015). Beyond requiring that the sister pairs be phylogenetically independent so that they are mutually monophyletic and do not overlap on a phylogeny, the method does not require knowledge of the deeper topology of the tree.

The method of sister pairs allows an uncomplicated means of estimating and comparing relative diversification rates and rates of molecular evolution, while satisfying the requirements for statistical independence needed for the analyses and minimises the impact of the many assumptions required to construct time-resolved molecular phylogenies. For example, phylogenetic estimates of substitution rates can be strongly affected by choice and placement of fossil calibrations or the choice of phylogenetic model (Duchêne et al. 2014; Bromham et al. 2018). Sister pairs also offer relatively simple ways of controlling confounding artefacts such as the node-density effect. The node-density effect in phylogenetics refers to a phenomenon in which genetic distances tend to be underestimated along branches in a phylogeny where there are fewer intervening nodes (Venditti et al. 2006; Hugall and Lee 2007). Longer, unbroken branches are more likely to have ‘hidden’ substitutions that are not inferred by the maximum likelihood algorithm unless evidence for their existence arises from sequences that from intervening lineages that inherited that substitution. Because more substitutions are inferred along paths when there are more intervening lineages, substitution rate estimates could be inflated in clades where there are more species, leading to a spurious positive correlation between species richness and rates of molecular evolution. In a sister-pairs analysis, the effect can be ameliorated by selecting an even number of sequences for each of the two sister clades when estimating substitution rates (Lanfear et al. 2010b).

Despite these many advantages, a perceived disadvantage of sister-pair analyses is that they use only a subset of the available data, unlike tree-based analyses where all available tips in the phylogeny are included, as well as all internal branches. However, tree-based approaches are highly dependent on model-based inference of both speciation and substitution rates which are not only dependent on the assumptions of the model, but are also subject to sampling and inference biases such as incomplete sampling or the node-density effect. To provide a contrast to our sister-pair analyses, we also perform an analysis using the tree-based method of Webster et al. (2003), in which the root-to-tip molecular path length (sum of branch lengths) is regressed against the number of inferred nodes on the root-to-tip path accounting for covariation due to shared history via a linear mixed model (Webster et al. 2003; Pagel et al. 2006; Venditti et al. 2006; Venditti and Pagel 2010). This method is designed to detect the effect of punctuational bursts of substitutions or morphological traits related to speciation events, but could also detect correlated evolution between traits and speciation rates (Rabosky 2012).

Here, we examine pairs of sister clades at different taxonomic levels to investigate the relationship between synonymous and nonsynonymous substitution rates and diversification rates in ray-finned fishes (Actinopterygii). We also perform tree-based analyses for mitochondrial and nuclear gene sequences and for substitutions inferred from a published supermatrix (Rabosky et al. 2018). We investigate potential confounding effects of life history, depth and latitude and examine whether molecular rate variation has a role in mediating their relationships with fish diversity.

## Materials and Methods

### Molecular Phylogenies

We used two alternative phylogenies to select species pairs for analysis. First, we used a recent ray-finned fish phylogeny incorporating almost all presently described species (Rabosky et al. 2018). The backbone for this phylogeny has been constructed via maximum likelihood estimation using a concatenated matrix of 27 mitochondrial and nuclear coding genes for 11,638 species. The timescale was derived from a penalised likelihood rate smoothing procedure (treePL; Smith and O’Meara 2012). The molecular backbone contains representatives of every extant ray-finned fish family, with an accompanying taxonomy that places nearly all named actinopterygian species on the tree. The authors extended this backbone to make a series of complete trees by inserting species without molecular data using a birth–death-sampling interpolation procedure. This produces a distribution of 100 complete ‘all-taxon-assembled’ time trees with 32,100 tips total (Chang et al. 2020), which we obtained from https://fishtreeoflife.org/.

All phylogenies have some limitations in terms of taxon sampling, analysis method or assumptions made in the analysis. Since Rabosky et al. (2018) take a supermatrix approach, the molecular data are sparse and some genes are represented in many more taxa than others, particularly the mitochondrial barcoding gene cytochrome oxidase I (COI). This means that the data are not equally informative at all resolutions and backbone relationships may be uncertain. To test whether the choice of underlying phylogeny influenced the results, we also ran the analysis on the Euteleost Tree of Life dataset (Betancur-R et al. 2013). The Euteleost Tree of Life backbone phylogeny consists of 1416 taxa and was reconstructed via maximum likelihood. While this phylogeny has fewer species than the phylogeny of Rabosky et al. (2018), the data matrix is more complete, meaning that most species have most of the sequences available.

### DNA Sequence Selection

**Fig. 1:**
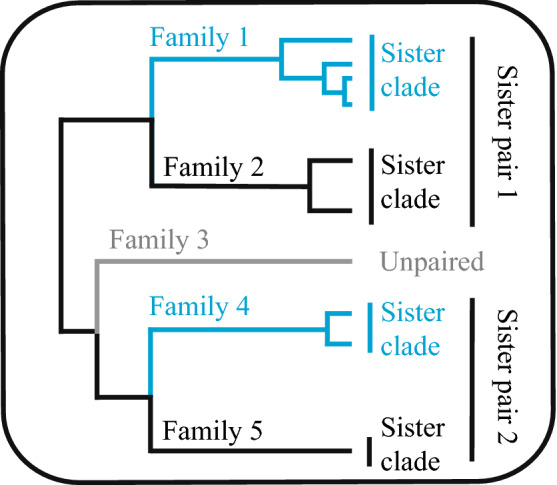
Terminology used to describe sister pairs. A sister pair is composed of two sister clades. The sister clades are the two daughter lineages of an ancestral lineage and all their known descendants. They do not share their MRCA with any other clades on the phylogeny. For inferring molecular rates, the larger sister clade would be pruned at random so that the same number of sequences would be analysed on each side

A sister pair consists of two sister clades that share an immediate common ancestor. We use the word ‘sister pair’ for the containing group and ‘sister clades’ for each of the two daughter groups throughout, that is each sister clade is one half of a sister pair (Fig. 1). To maximise the amount of available data for the sister-pairs analysis, we chose genes to provide broad coverage across many sister pairs.

For the analysis based on the phylogeny of Rabosky et al. (2018), the genes chosen were mitochondrial 12S and 16S ribosomal RNA (12s, 16s), cytochrome oxidase I (*co*I) and cytochrome B (*cyt*B), and nuclear recombination activating gene 1 (*rag*1). These sequences were obtained from GenBank using the accessions provided by Rabosky et al. (2018). All four mitochondrial genes (Mitochondrial Total sequences, labelled Mito.All) were concatenated for the tree-based analysis and to estimate total substitution rates. For determining relative nonsynonymous and synonymous substitution rates (dS and dN), only protein-coding sequences can be used, and so *co*I and *cyt*B coding sequences (Mitochondrial Coding sequences, labelled Mito.Coding) were concatenated without the rRNA sequences. The nuclear data consisted only of protein-coding sequences and so were used for inferring Total substitutions, dS and dN (Nuclear Coding with *rag*1, labelled Nuc.RAG1).

For the analysis based on the published phylogeny and dataset of Betancur-R et al. (2013), we selected all 19 protein-coding nuclear exonic markers taken from this dataset (labelled Nuc.ETOL). Non-protein-coding and intron data were not used. This dataset allowed for a smaller but more data-rich set of sister pairs, which could provide more power for substitution rate estimation. We used 19 loci from this study, each of which includes exon sequence from nuclear protein-coding genes (ENC1, FICD, GlyT, KIAA1239, Myh6, Panx2, Plagl2, Ptchd1, Rag1, Rag2, rh, Ripk4, SH3PX3, SIDKEY, SREB2, SVEP1, Tbr1, VCPIP, Zic1).

### Sister-Pair Selection

**Fig. 2:**
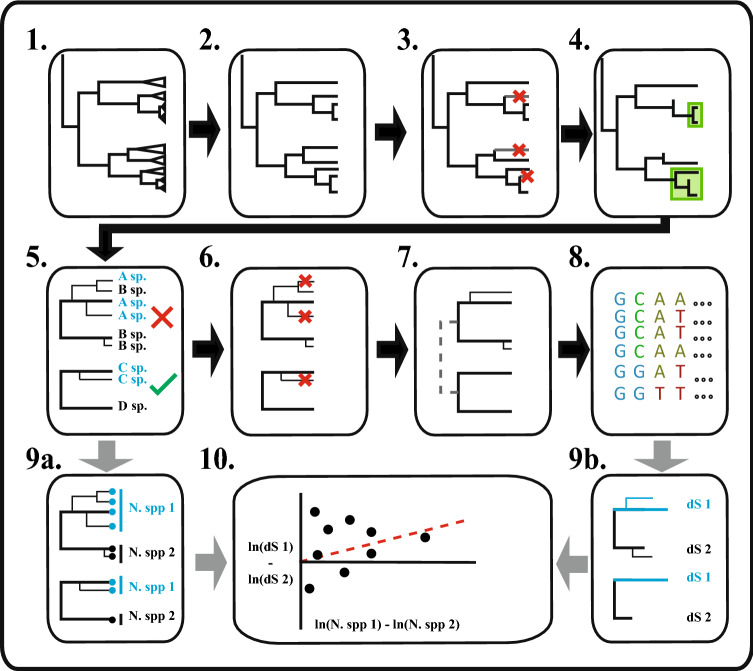
Schematic of the process used to select sister pairs and analyse the relationship between molecular evolution and diversification. (1) Tips are grouped into the selected taxonomic level, such as families. (2) A family-level tree is produced by pruning each family to a single tip. (3) Families with nosequence data available in the downloaded data set, if any, are removed. (4) From the remaining tips, we select all immediately adjacent pairs of tips (once without sequence data are removed) as our sister pairs. (5) To form our Filtered dataset, we perform checking for mutual monophyly of the clades within each sister pair. Pairs where one or more genera are present in both clades in > 80% of all-taxon-assembled trees in Rabosky et al. (2018) are removed. This was only done for the Rabosky et al. phylogeny, as most families in the Euteleost Tree of Life tree are monophyletic and no all-taxon-assembled trees are available. (6) To reduce the node-density effect, we randomly delete tips from the larger clade of each sister pair until both clades have the same number of tips. (7) To create outgroups for analysis, each sister pair is attached to its nearest sister pair to form a quartet. (8) Molecular sequence alignments are produced for each quartet. (9) Our final dataset consists of a set of contrasts, each consisting of a pair of sister clades, for which we have data on (a) clade sizes for each pair, and (b) molecular substitution rates (branch lengths) inferred from the sequence alignment. (10) A regression is conducted for contrasts in log clade sizes against contrasts in log substitution rates to test for a relationship between substitution and diversification rates

The sister-pair selection process is illustrated in Fig. 2. The same procedure applies to both phylogenies. We first assigned all species on the backbone phylogeny to families according to the molecular taxonomy provided with the tree on https://fishtreeoflife.org. We then identified families in which at least one tip possessed the target sequences for the dataset—rRNA, *co*I and *cyt*B for Mito.All, *co*I and *cyt*B only for Mito.Coding, and *rag*1 for Nuc.RAG1. We produced a tree in which each of these families was represented by its own tip, the first in the order in which the phylogeny was presented in the tree file, and all other families were absent. From this tree, we chose all pairs of families that descended from the same node, with no intervening tips – equivalent to all remaining clades of size 2. The pairs obtained by this method constituted our full set of “family-level” sister pairs. To examine the effect of pair age and taxonomic level, we repeated this procedure using a tree in which tips represented genera with available sequence rather than families. We refer to these as our “genus-level” pairs. At both family and genus levels, this was repeated for each of the Mito.All, Mito.Coding and Nuc.RAG1 target sequences for a total of six sets of sister pairs (see Table 1).

**Table 1:** Summary of sister-pair datasets for examining the relationship between substitution rates and diversification rates

Rank	Alignment	Genes	Phylogeny	Substitutions	Number of pairs			
					All	Mono only (not tested)	Filtered (Mono + Welch)	Full (Welch)
Families	Mito.All	*rRNA, coI, cytB*	Rabosky	Total	54	41	41	50
Families	Mito.Coding	*coI, cytB*	Rabosky	dS	88	59	58	77
				dN	88	65	63	78
Families	Nuc.RAG1	*rag1*	Rabosky	Total	119	82	82	113
				dS	119	44	44	65
				dN	119	45	43	62
Genera	Mito.All	*rRNA, coI, cytB*	Rabosky	Total	183	71	48	64
Genera	Mito.Coding	*coI, cytB*	Rabosky	dS	475	193	144	235
				dN	475	204	83	138
Genera	Nuc.RAG1	*rag1*	Rabosky	Total	547	249	121	256
				dS	547	250	199	355
				dN	547	239	131	247
Families	Nuc.ETOL	19 nuclear protein-coding exons	Betancur-R	Total	118	98	78	–
				dS	118	98	75	–
				dN	118	98	78	–

Rank is the taxonomic rank at which pairs are selected. Three marker sets are used for the main analysis, with the Mito. All set including non-coding sequences for additional power in estimating total substitution rates. An additional analysis uses the euteleost phylogeny of Betancur-R et al. (2013) and an associated dataset of 19 nuclear exonic sequences (Table S2 available as Supplementary Information). Datasets are further divided by the type of substitution rate estimated. Different substitution types may have different numbers of usable pairs because some PAML analyses fail due to short branch lengths or because pairs are deleted by the Welch filter, which removes shallow pairs with high variance in branch length estimates. The number of pairs remaining is shown after filtering for mutual monophyly (Mono) and after conducting the Welch filter(Welch). The monophyly filter removes pairs in which members of one or more genera are found on both sides of the pair in 80% or more of the all-taxon-assembled trees incorporating taxa without molecular data. The set of pairs filtered by both Monophyly and Welch filters formed our Filtered dataset, whereas the set of pairs filtered only by the Welch filter constituted our Full dataset

For the phylogeny of Rabosky et al. (2018) only, because of the sparse nature of the sequence data in the backbone phylogeny, genus-level relationships were often poorly resolved, which renders true clade sizes uncertain and may affect the comparative analysis. To examine this, we also produced a ‘Filtered’ set by performing an additional filter for mutual monophyly of pairs to the ‘full’ sets of sister pairs. If members of any genus were found in both sister clades of a given sister pair in a given tree of the all-taxon-assembled pseudoposterior (generated by stochastic placement of tips without molecular data), we considered the sister clades not to be mutually monophyletic in that tree. For our optional filtration step, we removed all pairs which were not mutually monophyletic in 80% or more of the 100 all-taxon-assembled trees. We performed this step for each of the six datasets described above (Table 1), producing separate ‘Full’ and ‘Filtered' analyses for each. Comprehensive code for reproducing our initial sister-pair selections is available online (https://github.com/amritchie/fish-diversity-molecular-evolution).

As a check for possible confounding or mediation relationships between either clade size or substitution rates and life history, we also attempted to construct a set of datasets filtered for the availability of life history data, including body size and depth range. (Supplementary Methods S2 available as online Supplementary Information). However, we found that this extra filtering step reduced the power sufficiently that results found on the Full and Reduced datasets could not be confirmed. We provide some comments on this issue in the Discussion.

### Species Richness (Clade Sizes)

Each of our comparisons consist of two closely related sister clades, defined by a unique shared common ancestor that is shared with no other such pairs in the dataset. Because of this, each sister clade has had the same amount of time to accumulate species. Therefore, the number of extant lineages in each sister clade can be used to infer differences in net diversification rate between each sister lineage: because they are defined by the same ancestor, they are the same age, and any difference in species richness must indicate a difference in the speciation rate or extinction rate or both. Therefore, we want to know how many extant species can be assigned to each sister clade of each comparison.

If the phylogeny contains all extant species, then we can use clade size to estimate net diversification rate. The Rabosky et al. (2018) tree includes nearly all named actinopterygian species; however, the placement on these species varies between alternative phylogenetic solutions. We estimate clade sizes for in our sister pairs by averaging the number of tips descending from the most recent common ancestor (MRCA) of that clade in each of the 100 all-taxon-assembled sister pairs. In detail, for each all-taxon-assembled tree, we counted how many tips were contained in each sister clade, including species with no molecular data. We then averaged these clade sizes over all 100 all-taxon assembled trees and took these values as our approximate clade sizes. This procedure accounts for uncertainty in the placement of species without molecular data. We checked whether these clade sizes were a good match to the sizes obtained from formal taxonomies, as done for example in (Lanfear et al. 2010a).

To check whether the average clade sizes produced in this way are a good reflection of species richness, we also generated species richness estimates from formal taxonomy. We used Family- and Genus-level classifications from FishBase and produced clade sizes by counting all valid species names for all families or genera within each sister clade of each sister pair. Because the correlation between clade sizes produced from all-taxon-assembled trees and from formal taxonomies was strong (Supplementary Figs. S1, S2), we used the averaged clade sizes only.

### Molecular Rate Estimation

We used the molecular data to estimate average synonymous (dS) and nonsynonymous (dN) substitutions per site across root-to-tip paths in each sister clade within each sister pair. To do this, for each sister pair, we collected all tips that had target genes available for each sister pair (Fig. 2). We then evened out the number of tips in the two sister clades within each pair by sampling the appropriate number of tips uniformly at random from all tips with sequence data in the larger sister clade. This step helps reduce the impact of the node-density artefact, which can result in clades with fewer tips having lower estimates of substitution rate (Hugall and Lee 2007).

Sister pairs comparisons of rates of molecular evolution require an outgroup to allow the position of the root to be defined. The outgroup should ideally be the nearest relative to the sister pair that is certain not to fall within the sister comparison, yet has the minimum length of branch joining the two pairs in order to minimise saturation. For the sparse dataset of Rabosky et al. (2018), to ensure an outgroup with sufficient data coverage, we selected the most closely related sister pair to form a quartet. Each sister pair in the quartet acts as the outgroup to the other sister pair, rooting the tree and allowing substitutions to be inferred for each sister pair. For the more complete dataset of Betancur-R et al. (2013), we simply selected the nearest single tip with the most complete data as outgroup.

For both phylogenies, we produced preliminary alignments of all mitochondrial and nuclear genes using MAFFT (Katoh and Standley 2013) with the L-INS-I algorithm. We then generate an alignment for each comparison by extracting just the tips included in each quartet. These alignments were then pruned to remove any codons that did not have data for at least one tip from each sister in each pair and from the corresponding outgroup. We removed any pairs for which 100 or fewer bases would have remained after pruning. For the phylogeny of Rabosky et al. (2018), the pruned alignments averaged approximately 1.2 kb for nuclear data and up to 2 kb for full mitochondrial datasets (for distribution of alignment lengths for each comparison see Figs. S3, S4 available as Supplementary Information). For the phylogeny of, pruned alignments averaged 4.3 kb (ranging from 0.6 to 10.3 kb).

For each alignment, we first inferred the placement of tips within each sister clade (phylogenetic topology) via maximum likelihood tree search using RAxML v8 (Stamatakis 2014): topology was constrained to ensure each sister pair and its dependent sister clades were monophyletic. We estimated average substitutions along all branches of these quartet phylogenies separately for each dataset (see Table 1) using baseml within the PAML 4 software suite (Yang 2007) using the RAxML topology. Similarly, for each clade within each sister pair, we estimated dS (average synonymous substitutions per site) and dN (average nonsynonymous substitutions per site) with codeml in PAML 4. For the underlying mutation model in codeml and the substitution model in baseml, we used the general time-reversible (GTR) model with gamma-distributed rate variation and 4 gamma categories. A separate codon model was assigned to each sister clade within each sister pair in the quartet. The molecular clock option was not used, meaning that we inferred average substitutions per site rather than branch-specific molecular rates. For each sister clade in each sister pair, a phylogenetic average substitution rate was estimated by successively averaging the lengths of sister branches from the tip down and adding the result to the length of the parent branch. This provides an estimate of the average substitutions per site from the root of a clade to each tip of the clade while ensuring that the estimate is not dominated by speciose subclades, so the contrast in this estimate between sister clades reflects difference in the substitution rate between the sister clades. We removed any pairs for which the estimated average number of synonymous substitutions per site was greater than 2, as this would likely indicate saturation of the available sequence.

### Regression Analysis of Sister Pair Contrasts in Molecular Rates and Diversification

To explore the relationship between molecular rates and diversification rates, we calculated differences between the logarithms of the phylogenetic average dS and dN values for the two clades within each sister pair. Likewise, to estimate contrasts in net diversification rate, we used the difference between the logarithm of the clade size (as calculated from the average of the number of tips in the 100 all-taxon-assembled phylogenies) between the two sister clades of each sister pair. We performed additional tests and data transformations to check whether the size of contrasts was affected by the depth of the pair, as represented by the inferred age of the MRCA of each sister pair, or by the ancestral value of the diversification or substitution rates (using values of time and rates derived from the published phylogeny: see Table S1 and Supplementary Methods S1 available as online Supplementary Information), but these analyses did not materially alter the results, so the analysis presented here uses comparisons without any adjustment for age or ancestral values.

As suggested by Welch and Waxman (2008), we also tested for a negative relationship between the absolute value of the normalised molecular rate contrasts and the square root of the inferred age of the pair (derived from the published phylogeny: see Supplementary information). This relationship arises because inferred substitution rates in shallow pairs have higher variance due to the uncertainty of rate estimation with few differences between sequences. The presence of these pairs can reduce the power of the test and lead to false negative results. As suggested by the study, we successively remove the shallowest pair from each dataset until no negative relationship remains, as tested by a simple linear model (Figs. S21, S22, S23 available as Supplementary Information). We repeated this test using Total, dS and dN substitution rate estimates, removing different numbers of pairs for each dataset. Following this procedure, we also observed a distinct grouping of pairs with very small mean substitution rates that nevertheless had extremely large contrasts in substitution rates, which we attributed to the unreliability of the maximum likelihood optimization in PAML for very short branches. We, therefore, removed these points from the analysis (summarised in Table 1). Following this “Welch Filter”, we tested for a relationship between molecular rate and diversification rate contrasts by ordinary least squares regression through the origin using R v3.13 (R Core Team 2019). The regression is forced through the origin because we do not have any expectation regarding the direction of the substitution rate contrast for two clades of identical size, so the expected substitution rate contrast must be zero.

### Tree-based Analysis

For the tree-based analysis, we analysed three subsets of the molecular backbone phylogeny inferred by Rabosky et al. (2018). The first was the complete molecular backbone. This is a large tree of 11,638 tips with substitutions inferred from a supermatrix of 27 genes. Although being a supermatrix with significant incomplete data means that inferred substitutions may be less reliable in some parts of the tree and means that we cannot determine separate effects for nuclear and mitochondrial genes, this is by far the most comprehensive dataset presently available and allows the most powerful test for whether a correlation between molecular evolution and diversification is likely to exist. To investigate results for mitochondrial and nuclear sequences separately and enable comparison with our sister-pair analysis, we construct a second set of substitution rate inferences directly from a dataset of all species in the molecular backbone tree having sequences for the full set of four mitochondrial genes (Mito.All) used in the sister-pair analysis (*12s*, *16s*, *coI* and *cytB*), and a third dataset of all species with nuclear *rag*1 sequences is available (Nuc.RAG1). We removed 50% gapped positions and aligned sequences using MAFFT. We then fixed the topology of the tree using the molecular backbone phylogeny. We estimated total substitutions per site using RAxML with the GTR + Gamma substitution model and the − f e option to infer substitutions on the fixed backbone and removed the outgroup. After these measures, the mitochondrial alignment consisted of 1133 species and 1.6 kb of sequence, while the nuclear alignment consisted of 3033 species and 1.38 kb.

For each root-to-tip path in the three RAxML phylogenies (based on the Rabosky et al. 2018 dataset), we calculated the number of nodes and the total path length in substitutions per site. The full backbone phylogeny only includes species with available sequence, so it does not include the approximately 31,000 fish species for which molecular data was not available. Therefore, most nodes along the root-to-tip path are missing in the trees produced from mitochondrial data and the *rag*1 nuclear gene. These can be dealt with either by simply using the number of nodes from root to tip in each RAxML phylogeny, as in Webster et al. (2003), or averaging the number of nodes for the same tip over the 100 all-taxon-assembled trees, as was done in Rabosky et al. (2018). In practice, we found that the root-to-tip node numbers produced by these two methods were highly correlated and produced very similar results for all parameters in both the subsequent regression and node-density test. We, therefore, use the simple counts of nodes from root to tip for all analyses.

We performed a least squares regression across root-to-tip paths using a variance–covariance matrix derived from the phylogeny to account for the shared history of each path. We used the number of nodes as the independent variable with an intercept, and the path length as the response. Significance of the relationship was determined by a likelihood ratio test of the fitted model against a model with the coefficient of node number fixed to 0. Because this method is vulnerable to the node-density effect, we also use a previously described test (Venditti et al. 2006) to check for the presence of this artefact. To do this, we fit a three-parameter model with

$${\text{nodes}}\,{\text{on}}\,{\text{path}} = {\upbeta } \times {\text{path}}\,{\text{length}}^{{1/{\updelta }}} + {\text{intercept}} + \varepsilon,$$

where $\beta$ is the regression coefficient, residual vector $\epsilon$ has a multivariate normal distribution with covariance matrix derived from the phylogeny, and $\delta$ is a parameter controlling the degree of non-linearity in the response. An optimised value of $\delta$ > 1 in combination with a significant likelihood ratio test for $\beta$ > 0 is taken as evidence for the presence of the node-density effect (Venditti et al. 2006). Simulations indicate that the test for this artefact is quite robust, with a false positive rate of only 1.7% (Venditti et al. 2006). We ran this test with both node numbers and path lengths as the response model and inspected the resulting plots in cases of serious discrepancy, as the three-parameter model can be affected by outliers or groups of tips with the same node number.

## Results

### Data Selection

The number of pairs selected by the procedure above is shown in Tables 1 and 2, including the Full Dataset (all sister pairs) and the Filtered Dataset (after additional filtering steps). Imposing a requirement that all genera be monophyletic resulted in the removal of up to three quarters of the initial genus-level pairs (Table 1). Additionally, the procedure of Welch and Waxman (2008) for removing shallow sister pairs resulted in greater reductions for dN than dS datasets because of the approximately tenfold smaller number of nonsynonymous substitutions.

**Table 2:** Regressions through the origin relating contrasts in log substitution rates to contrasts in transformed clade sizes across sister pairs

Phylogeny	Mono. Filter	Rank	Sequence set	Subst. Rate	No. pairs	Coeff	Std. Err	*t*	P value ( >|*t*|)
Rabosky et al. (2018)	Full	Families	Mito.All	Total	50	0.82	0.68	1.20	0.24
			Mito.Coding	dS	77	− 0.68	1.11	− 0.61	0.54
				dN	78	− 0.07	0.35	− 0.21	0.83
			Nuc.RAG1	***Total***	***113***	***0.92***	***0.25***	***3.63***	** < *0.01***
				***dS***	***65***	***0.72***	***0.28***	***2.60***	***0.01***
				dN	62	0.47	0.33	1.43	0.16
		Genera	Mito.All	Total	64	0.07	0.55	0.12	0.90
			Mito.Coding	dS	235	0.08	0.14	0.62	0.54
				dN	138	− 0.05	0.10	− 0.53	0.60
			Nuc.RAG1	***Total***	***256***	***0.22***	***0.10***	***2.14***	***0.03***
				dS	355	0.02	0.08	0.29	0.77
				dN	247	0.18	0.10	1.90	0.06
	Filtered	Families	Mito.All	Total	41	0.97	0.79	1.24	0.22
			Mito.Coding	dS	58	0.24	0.57	0.42	0.68
				dN	63	0.03	0.37	0.07	0.94
			Nuc.RAG1	***Total***	***82***	***0.74***	***0.31***	***2.38***	***0.02***
				dS	44	0.43	0.37	1.17	0.25
				dN	43	0.50	0.42	1.20	0.24
		Genera	Mito.All	Total	48	− 0.19	0.55	− 0.35	0.73
			Mito.Coding	dS	144	− 0.05	0.13	− 0.40	0.69
				***dN***	***83***	***0.21***	***0.10***	***2.13***	***0.04***
			Nuc.RAG1	Total	121	0.32	0.19	1.71	0.09
				dS	199	− 0.02	0.11	− 0.19	0.85
				dN	131	0.07	0.14	0.49	0.63
Betancur-R et al. (2013)	Yes	Families	Nuc.ETOL	***Total***	***78***	***0.64***	***0.30***	***2.13***	***0.04***
				***dS***	***75***	***0.81***	***0.36***	***2.26***	***0.03***
				dN	78	0.08	0.21	0.36	0.72

Regressions were conducted on the phylogenies of Rabosky et al. (2018) and Betancur-R et al. (2013), and with or without filtering pairs for mutual monophyly of formal taxa (Mono. Filter). Data is shown for mitochondrial sequences with rRNA (Mito.All) and without (Mito.Coding). Nuclear data consists of RAG1 only for the Rabosky tree and of 19 nuclear marker from the Euteleost tree of life (ETOL) for the Betancur tree. Total, synonymous (dS) and nonsynonymous (dN) substitution rates are tested as predictors of clade size. *t* statistics and P values are for Wald tests. Results with P < 0.05 are in italics

### Sister-Pair Analysis

For each of our datasets (listed in Table 1), we performed linear regressions of transformed clade size contrasts against log dS or dN substitution rate contrasts (Table 2). For Full Dataset comparisons chosen from the Rabosky et al. tree, clade size had a significantly positive relationship with Total rate of molecular evolution for the nuclear sequences at both family and genus level. Clade size was also significantly associated with dS at family level. For the Filtered Dataset, there was a significant relationship between total nuclear substitution rate at the family level and for nonsynonymous mitochondrial substitution rate (dN) at the genus level. For the comparisons chosen from the Euteleost Tree of Life, both total substitution rate and dS were significantly positively associated with clade size.

### Tree-Based Analysis

The tree-based analysis related the number of nodes on each root-to-tip path on the full backbone, mitochondrial and nuclear trees to the total number of substitutions estimated along the path (the path length) (Table 3, Figs. S13, S14 available as Supplementary Information). The values with path length as response variable are shown because inspection of the associated plots indicated that the fitted curves for the mitochondrial tree were strongly affected by outlying values when node number was the response variable. Significant positive correlations were discovered for the backbone tree (P < 10^–6^) and the mitochondrial tree (P < 10^–4^). The nuclear tree did not show a significant correlation between path lengths and number of nodes per path. For the mitochondrial tree, $\delta$ is much greater than 1, indicating that some of the trend may be a node-density effect caused by underestimation of long branches. For the Rabosky et al. backbone tree, $\delta$ is close to one and becomes slightly less than one if the node path is used as response variable (0.95), indicating a small or no contribution to the significant positive trend from the node-density effect.

**Table 3:** Results of the tree-based analyses performed on the full molecular backbone of Rabosky et al. (2018) and two sampled trees with inferred mitochondrial and nuclear substitutions

Tree	Slope	Likelihood ratio	P value	$\delta$
Backbone	$2.4\times {10}^{-4}$	27.05	$1.98\times {10}^{-7}$	1.03
Mitochondrial	$2.4\times {10}^{-3}$	25.80	$3.79\times {10}^{-5}$	1.98
Nuclear	$1.2\times {10}^{-5}$	0.54	0.46	1.00

The results are shown with root-to-tip path length as the response variable. A significant likelihood ratio test (bold type) indicates a likely correlation between substitutions and node numbers. The combination of a significant likelihood ratio test and a $\delta$ curvilinearity value greater than one indicates the likely presence of the node-density artefact in the Actinopterygian tree. Trends are plotted in Figs. S13, S14 available as Supplementary Information

### Power to Detect Rate Differences

If molecular rates are too slow in the loci analysed to generate consistent difference in branch length, or if the pairs used diverged too short a time ago, there may be too few sequence differences to correctly reconstruct the difference in molecular rates. We consider the expected effect of the number of pairs, relative age of pairs and the length of the alignment on the ability to correctly infer substitution rate differences. For the tree-based analyses, the alignments were about 1.38 kb for the nuclear analysis and 1.6 kb for the mitochondrial genes. Our sister-pair alignments averaged 1.2 kb for nuclear and mitochondrial coding alignments (Nuc.RAG1, Mito.Coding) and 2.5 kb for the full mitochondrial alignment (Mito.All). Sister-pair analyses of similar sized datasets have yielded significant results in previous studies (Bromham et al. 2015). The mean age of sister pairs in this study was about 74 million years for families and 48 million years for genera, and molecular rates were on the order of ${10}^{-3}$ substitutions per site per million years. Without resorting to extensive simulations, we consider the informativeness of the dataset by approximating the number of substitutions for our average alignment as a Poisson distribution, with mean 74 Myr $\times {10}^{-3}\mathrm{ subs }/\mathrm{ site }/\mathrm{ Myr} \times 2.5\times {10}^{3}$ sites = 185 substitutions. We can then consider how frequently the contrast in underlying rates of molecular evolution would show up as positive contrasts in the number of substitutions in each clade within each sister pair. Assuming a conservative average 10% difference in underlying substitution rates between sister pairs, about 82% of underlyingly positive rate contrasts would show up as positive substitution rate contrasts for the family-level mitochondrial dataset, whereas that number would only be about 67% for the nuclear genus-level dataset with average 1.2 kb alignments. By this calculation, adding an extra marker with 1.5 kb of alignment would improve the power to reconstruct substitution rate contrasts by about the same amount as adding 6 additional sister pairs. In the case of the fish data, having more stringent pair selection, requiring taxa with more available sequences to increase the alignment length, would have reduced the number of possible pairs by considerably more than half a dozen pairs. Therefore, we consider our sister-pair selection to be as good as practically possible given the available data, and that given the available data, we could not have increased our power to detect consistent rate differences by only choosing pairs with longer alignments.

## Discussion

Our results provide evidence for a relationship between molecular evolution and diversification in ray-finned fish, with significant positive associations between clade size and rates of molecular evolution in nuclear sequences. This association was found for nuclear total substitution rates and synonymous substitution rates, as well as for mitochondrial nonsynonymous substitution rates (in the Filtered dataset only). The result is consistent with the finding of a relationship between root-to-tip path lengths and number of nodes on the full backbone phylogeny of Rabosky et al. (2018).

Our results are necessarily dependent on the accuracy of the phylogenies and taxonomies used, which may affect estimates of both diversification rates and molecular rates, in particular if the relationships between sister families or genera are reconstructed incorrectly. However, the findings for nuclear nonsynonymous substitution rates are replicated on two separate phylogenies with different molecular datasets and are, therefore, unlikely to be artefacts of particular markers or of the details of phylogenetic reconstruction. A tree-based analysis of the complete supermatrix from Rabosky et al. (2018) also found an association between molecular path lengths to node counts along root-to-tip paths, as has been found in many other taxa across the tree of life, and this is probably not primarily a result of the node-density effect based on a value of $\delta$ close to 1. This relationship is also found in a mitochondrial tree-based dataset, though here the node-density effect cannot be ruled out. We, therefore, consider that these results are consistent with a general association between nuclear synonymous substitution rates and diversification rates in bony fish.

These results from both sister-pairs analyses and whole-tree analyses suggest that ray-finned fish conform to a widespread relationship between speciation and molecular divergence (Webster et al. 2003; Pagel et al. 2006; Venditti et al. 2006), consistent with previous studies in plants, birds and other reptiles (Lanfear et al. 2010a; Eo and DeWoody 2010; Bromham et al. 2015). Detecting an association between substitution rates and clade size does not distinguish between alternative explanations for the relationship between diversification rate and rate of molecular evolution. However, the positive relationship between nuclear synonymous substitutions and diversification rates from our sister-pairs analysis indicates an association between mutation rate and diversification. This observation suggests a causal direction for the association. A correlation between mutation rate and diversification rate could arise if generation of variation during molecular evolution drives reproductive isolation or ecological divergence (Hua and Bromham 2017). Higher mutation rates cause more rapid accumulation of Bateson–Dobzhansky–Muller incompatibilities, speeding the acquisition of genetic barriers between species. It is more challenging to think of a plausible way that a higher rate of diversification could consistently raise the mutation rate. If speciation involves a strong population bottleneck it could cause the fixation of slightly deleterious changes to DNA repair proteins, but presumably these would be subject to negative selection after the speciation event to reduce the mutation rate. Further, in this instance we might expect to see strong relationships between dN rates and diversification rates, whereas this is not found for nuclear data in our analysis and is only found in one Filtered dataset for mitochondrial data. An association between net diversification and synonymous substitution rate could potentially be generated if lower mutation rate was linked to higher rates of extinction, for example through reduced capacity to respond to environmental change, though this would be challenging to evaluate given the lack of independent evidence of extinction rates in bony fish lineages across the phylogeny.

These results add to a growing picture linking change at the genomic level to the generation of biodiversity. If lineages that produce more diversity tend to have higher rates of molecular evolution, and particularly if this relationship is observed for synonymous substitution rates which are considered to predominantly reflect differences in the underlying mutation rate, this suggests that increased supply of changes to DNA sequences is somehow connected to increased speciation rate (or decreased extinction rate or both). Since the genes we have examined here are predominantly “housekeeping genes” associated with basic biochemical, genetic or metabolic functions, it seems unlikely that the association between rate of molecular change and diversification is primarily driven by selective processes such as competition, adaptation to new niches, mate recognition or reinforcement. Instead, the results are consistent with higher mutation rate driving a higher substitution rate which drives faster genome divergence and more rapid completion of speciation through genomic incompatibility.

Life history variation is a potential confounding or mediating factor in an analysis of the association between molecular rates and diversification. Body mass in fish has previously been found to be negatively associated with molecular rates (May et al. 2020) and has been shown to associate with diversification rates for reef fish, although this association was positive, whereas the relationship in our data was consistently negative (Siqueira et al. 2020). Other traits known or believed to associate with fish diversity. Specifically, depth range has been to correlate with extant species richness (Smith and Brown 2002; Fisher et al. 2010), but this may be the result of accumulation of species over longer stable time periods rather than diversification rate variation. Unfortunately, it was not possible to test comprehensively for these effects because, for most of our datasets, adding an additional filtering step for the availability of life history variables reduced the power of the analysis so much that the relationship between molecular rates and diversification rates could no longer be detected. The one dataset where a weak relationship remained (Full data, Family level, Nuc.Rag1) indicated that a model including both a body size measure (maximum length) and synonymous substitution (dS) rates explained significantly more variation in clade size than a model with body size alone, suggesting that the observed relationship between dS rates and clade sizes is not wholly explained by an indirect relationship with body size. However, the utility of this test is low because of the reduced power of the dataset and the lack of confirmation from other substitution rate types. It will be valuable for future studies to revisit this question as more molecular data are obtained for resolving genus- and species-level relationships across a wider range of ray-finned fish taxa.

It has previously been suggested that higher rates of mutation at warmer temperatures lead to faster molecular evolution and more rapid diversification (Rohde 1992; Gillman and Wright 2014; Orton et al. 2019) and that fish diversification is associated with temperature (Wright et al. 2011), although the exact mechanism linking temperature and mutation is unclear. These studies identify interesting correlates of diversification rate that suggest a role for rate of genomic divergence in fish diversification; however, it is likely to be one contributing factor in a complex pattern of diversification influenced by many environmental and evolutionary factors. The importance of glaciation history (Bernatchez and Wilson 1998; April et al. 2013; Miller and Román-Palacios 2021), recent radiations at high latitudes (Rabosky et al. 2018) and coral reefs (Cowman and Bellwood 2011; Cowman 2014) makes it clear that there is much more to the story of fish diversification than general patterns in energy and molecular evolution, but these remain a plausible part of the tale.

## Conclusion

We find evidence for a link between rate of molecular evolution and rates of diversification in ray-finned fish. Significant associations between clade size and synonymous, but not nonsynonymous, nuclear substitution rates suggest that the observed relationship may be driven by lineage-specific differences in mutation rate. We confirm this relationship on two separate phylogenies and molecular datasets and at family and genus taxonomic scales. Future investigations should be undertaken as more species-level molecular data accumulate, finer-scale taxonomic resolution is achieved, and more comprehensive data on life history and ecology are compiled.

## Supplementary Information

Below is the link to the electronic supplementary material.

Supplementary file1 (PDF 4124 KB)

## Data Availability

All data are from freely available online sources. Data and code for this project are also available online at https://github.com/amritchie/fish-diversity-molecular-evolution.
