# Supplementary material for: Diversification Rate is Associated with Rate of Molecular Evolution in Ray-Finned Fish (Actinopterygii)
Source: J Mol Evol. 2022 Mar 9;90(2):200–14. doi: 10.1007/s00239-022-10052-6 (PMC8975766; doi:10.1007/s00239-022-10052-6)
Supplement: Supplementary file 1 — Supplementary file1 (PDF 4124 KB) [file 239_2022_10052_MOESM1_ESM.pdf]

**Supplementary Information**

# Molecular rates and net diversification rates in ray-finned fish (Actinopterygii)

Table of Contents

***Supplement 0. Regression results with validation checks and re-standardisation.*** (Supplemental Methods S1, Table S1).

***Supplement 1. Sister pair analyses of life history and diversification rates.*** (Supplemental Methods S2, Table S2).

***Supplement 2. Validating clade size measures.*** (Figure S1-S2). Comparison of clade sizes derived from taxonomic counts with clade sizes derived from averages over all-taxon-assembled pseudo-posterior from Rabosky et al. (2018).

***Supplement 3. Alignment length distributions.*** (Figure S3-S4). Alignment length distribution of quartet alignments used to estimate substitution rates.

***Supplement 4. Pair age distributions.*** (Figure S5-S6). Tests for heteroskedasticity of sister pair clade size contrasts following transformation.

***Supplement5. Relationship between clade size and substitution rate contrasts.*** (Figure S7-*S15*).

***Supplement 6. Validation checks – Homoskedasticty of variables*** (Figure S16-S19).Tests for heteroskedasticity of transformed sister pair clade size and substitution rate contrasts.

***Supplement 7. Validation checks – Relationship of variance with pair age.*** (Figure S20-S23). Tests for residual effect of sister pair age on clade size and substitution rate contrasts following standardisation.

***Supplement 8. Whole tree analyses.*** (Figure S24-S25). Scatter plots showing relationship between substitutions and speciation events on root-to-tip paths for tree-based analyses of molecular and diversification rate correlation (Webster et al. 2003; Venditti et al. 2006).

***Supplement 9. Phylogenetic relationships among sister pairs.*** (Figure S26-S37). Visualisations of the phylogenetic relationships and ages of sister pair data sets used in the sister pair analysis of correlated molecular and diversification rates.

**Supplementary References.**

Rabosky DL, Chang J, Title PO, Cowman PF, Sallan L, Friedman M, Kaschner K, Garilao C, Near TJ, Coll M, Alfaro ME (2018) An inverse latitudinal gradient in speciation rate for marine fishes. Nature 559:392

Venditti C, Meade A, Pagel M (2006) Detecting the node-density artifact in phylogeny reconstruction. Systematic Biology 55:637

Webster AJ, Payne RJH, Pagel M (2003) Molecular phylogenies link rates of evolution and speciation. Science 301:478

# 0. Regression results with validation checks and re-standardisation

**Supplemental Methods S1. Validation checking and standardisation of substitution rate and clade size data.**

To ensure that sister pair contrasts in diversification rates (estimated from clade sizes) and substitution rates (estimated from substitution rates) are independent of their ancestral trait values and do not violate the assumption of homoscedasticity, we re-ran our main results after testing for and removing any relationship between the absolute contrast in the dependent variable for each sister pair and the mean of the two clades in each sister pair, which may be taken as an estimate of the ancestral diversification or substitution rate (see Freckleton 2000; Lanfear et al. 2010a). We therefore plotted the contrasts in clade sizes $N$ against their means, and performed Kendall rank-correlation tests to search for a strong relationship under different transformations of the clade sizes. Family-level contrasts showed no significant correlation with the standard log transformation, but a relationship remained in the four sets of Genus-level clade size contrasts, necessitating a different transformation of the data. By experiment we chose the power transformation $N^* = -1/N^{0.3}$, for which no correlation remained (Figures S16-S17). We conduct a similar test for the relevant branch length estimates (Figure S18-S19).

Following the recommendations of Garland et al. (1992), we standardised the variance of all contrasts by dividing through by the square root of the inferred age of each pair. This is recommended because the variance in trait values among lineages should increase with the amount of time since they diverged. We used the time estimates from Rabosky et al. (2018) as our estimate for this normalisation. We checked for a positive relationship between the normalised contrasts in transformed clade sizes and the square root of time (figures S20-S23). In all but one data set none was found, indicating that this normalisation factor was adequate. A weak negative correlation occurred in the mitochondrial dS data set at the genus level. Therefore we used a slightly weaker standardising factor of (pair age)$^{0.3}$ for this data set alone, which removed the correlation (Figure S9-S10 available as Supplementary Information).

**Table S1.** Regression results for data standardised following validation checks (See Supplementary Methods S1 and Supplements 6-7). Regressions through the origin relating contrasts in log substitution rates to contrasts in transformed clade sizes across sister pairs. Regressions were conducted on the phylogenies of Rabosky et al. (2018), and with or without filtering pairs for mutual monophyly of formal taxa (Mono. Filter). Data is shown for mitochondrial sequences with rRNA (Mito.All) and without (Mito.Coding). Nuclear data consists of RAG1 only for the Rabosky tree. for the Betancur tree. Total, synonymous (dS) and nonsynonymous (dN) substitution rates are tested as predictors of clade size. *t* statistics and P-values are for Wald tests. Results with p < 0.05 are in bold.

| Phylogeny | Mono. Filter | Rank | Sequence set | Subst. Rate | No. pairs | Coeff. | Std. Err. | *t* | P-value (>\|*t*\|) |
|---|---|---|---|---|---|---|---|---|---|
| Rabosky et al. (2018) | No | Families | Mito.All | Total | 50 | 0.82 | 0.68 | 1.20 | 0.24 |
| | | | Mito.Coding | dS | 77 | -0.68 | 1.11 | -0.61 | 0.54 |
| | | | | dN | 78 | -0.07 | 0.35 | -0.21 | 0.83 |
| | | | Nuc.RAG1 | ***Total*** | ***113*** | ***0.92*** | ***0.25*** | ***3.63*** | ***< 0.01*** |
| | | | | ***dS*** | ***65*** | ***0.72*** | ***0.28*** | ***2.60*** | ***0.01*** |
| | | | | dN | 62 | 0.47 | 0.33 | 1.43 | 0.16 |
| | | Genera | Mito.All | Total | 64 | 0.02 | 0.07 | 0.31 | 0.76 |
| | | | Mito.Coding | dS | 235 | 0.02 | 0.02 | 0.96 | 0.34 |
| | | | | dN | 138 | 0.00 | 0.01 | -0.29 | 0.78 |
| | | | Nuc.RAG1 | ***Total*** | ***256*** | ***0.03*** | ***0.02*** | ***2.22*** | ***0.03*** |
| | | | | dS | 355 | 0.00 | 0.01 | 0.11 | 0.91 |
| | | | | ***dN*** | ***247*** | ***0.03*** | ***0.01*** | ***2.14*** | ***0.03*** |
| | Yes | Families | Mito.All | Total | 41 | 0.97 | 0.79 | 1.24 | 0.22 |
| | | | Mito.Coding | dS | 58 | 0.47 | 1.32 | 0.35 | 0.73 |
| | | | | dN | 63 | 0.03 | 0.37 | 0.07 | 0.94 |
| | | | Nuc.RAG1 | ***Total*** | ***82*** | ***0.74*** | ***0.31*** | ***2.38*** | ***0.02*** |
| | | | | dS | 44 | 0.43 | 0.37 | 1.17 | 0.25 |
| | | | | dN | 43 | 0.50 | 0.42 | 1.20 | 0.24 |
| | | Genera | Mito.All | Total | 48 | -0.01 | 0.08 | -0.13 | 0.90 |
| | | | Mito.Coding | dS | 144 | -0.01 | 0.02 | -0.62 | 0.53 |
| | | | | ***dN*** | ***83*** | ***0.04*** | ***0.02*** | ***2.34*** | ***0.02*** |
| | | | Nuc.RAG1 | Total | 121 | 0.05 | 0.03 | 1.52 | 0.13 |
| | | | | dS | 199 | -0.01 | 0.02 | -0.61 | 0.54 |
| | | | | dN | 131 | 0.01 | 0.02 | 0.30 | 0.77 |

# 1. Sister pair analyses of life history traits and diversification

**Supplemental Methods S2. Sister pair analysis of life history and diversification rates**

The relationship between molecular evolution rate and diversification rate can be complicated by the presence of relationships in other life history and ecological traits with molecular evolution rate and diversification rate (Bromham et al. 2015). We attempted to examine potential confounding or mediating life history and ecological factors for the relationship between molecular rates and diversification rates.

Previous macroevolutionary studies have found a widespread positive relationship between diversification rates and latitude (Rabosky et al. 2018), and a positive relationship between diversification rate and body size at least in reef-associated fish families (Siqueira et al. 2020). Latitude and body size also correlate negatively with molecular rates across Actinopterygii, potentially creating confounding (latitude) and concealing (body size, using maximum body Length as a proxy) relationships (May et al. 2020). Additionally, depth is known to correlate negatively with molecular rates as one component of the relationship between molecular rates and temperature (Wright et al. 2011; May et al. 2020), while an association between depth changes and speciation has been demonstrated in the speciose rockfish genus (Ingram 2011), suggesting a possible relationship. Finally, taxa with higher proportions of reef-associated lineages have been shown to have higher diversification rates (Cowman and Bellwood 2011), though this is not replicated in more recent studies (Rabosky et al. 2018). Coral reef habitat is also associated with higher molecular rates (May et al. 2020), making this another possible confounding factor. We sought to determine whether these relationships were detected in our limited data sets, and whether incorporating them in the model affected the relationship between molecular evolution and diversification.

To obtain life history data, we used the online FishBase database (Froese and Pauly 2000) via the package ‘rfishbase’ for R (Boettiger et al. 2012; R Core Team 2019). Species names from the taxonomy developed by Rabosky et al. (2018) were matched against the FishBase nomenclature using

either valid names or a synonym listed in FishBase. We obtained data on maximum Length, maximum depth, and categorical data on habitat type from FishBase. We recoded habitat data as a binary variable which was given the value of 1 if the habitat field in FishBase included the term "reef-associated" and 0 otherwise. For latitude, we used the curated range centroids produced by Rabosky et al. (2018), which we found to be highly correlated with latitude midpoints calculated from maximum north and south values in FishBase. Data were collected for all valid ray-finned fish species names listed in FishBase.

To test for an effect of life history or environment in our dataset, we generated four sets of comparisons. We generated them as described for the molecular-only data sets above, except thatwe used only families or genera for which at least one measurement of each life history or ecological trait was available (not necessarily for the same tips). For each sister clade, a taxonomic average of all available life history and ecological traits associated with its descendent species in each all-taxon-assembled tree was produced by averaging trait values successively across each species, genus, and sister clade, similar to the procedure for substitutions. These values were then averaged across the 100 all-taxon-assembled posterior trees. For habitat, we found that the proportion of reef-associated species in each genus and sister clade was almost universally close to 1 or 0, so we recorded a 1 for each sister clade in which >= 50% of genera had a 1 for a majority of descendant species. These procedures resulted in data sets with complete trait and taxonomic data. The numbers of remaining comparisons are displayed in Table 3.

We selected the best fitting linear model regressing diversification rate contrasts against trait contrasts by minimising the Akaike Information Criterion (AIC) via stepwise regression using the R 'step' function. This was done for each trait data set separately. We then performed multiple regression with substitution rate contrasts to determine whether adding the contrast in substitution rates significantly

improved prediction of the diversification rate once life history and ecology had been incorporated. This test asks whether all the differences in net diversification rate can be explained by life history variation and chance, or whether there is a significant signal for differences in diversification rate that can be attributed to variation in rate of molecular evolution.

*Supplementary results and discussion*

Results of the life history regressions are shown in Supplementary Table S2. Stepwise regression universally selected either a null model with no predictors, or a model with Length only as a predictor. In all datasets but one, the relationship between substitution rates and clade sizes was not significant in the life history data even when it was significant in our Full and Reduced datasets, indicating that the additional filtering step for life history availability reduced the power of the dataset. The only life history dataset for which this relationship remained was for Family-level contrasts of nuclear synonymous substitutions (Nuc.Rag1, dS). For this dataset, we detected a significant improvement for a model including both Length and nuclear dS as predictors of clade size over a model only including Length (F-test, $p < 0.05$). However, given the low power of these datasets as well as the lack of a confirming signal in the Total nuclear substitution dataset, this result is low confidence and may change with additional data.

**Table S2.** Testing whether detected relationships between molecular rate and clade size contrasts are explained by life history. The Trait model is the best-fitting model predicting clade size based on life history, selected from a full model containing all life history traits by stepwise regression with AIC. In all cases, either a model with Length (maximum body Length) alone or a null model with no predictors was selected as the best model. The Rate Model is a model relating substitution rate contrasts to clade size contrasts with no life history. The final column shows the results of an F-test testing whether a multiple regression including life history traits and substitution rates explains significantly more variance in clade size than the Trait Model alone.

| Rank | Sequence | Subst. Type | Trait Model | Coeff. (Trait Model) | Pvalue (Trait Model) | Coeff. (Rate model) | P-value (Rate Model) | P-value (F-test – Trait + Rate > Trait Model) |
|---|---|---|---|---|---|---|---|---|
| Families | Mito.All | Total | ***Length*** | ***-1.36*** | ***0.004*** | -0.093 | 0.920 | 0.571 |
| | Mito.Coding | dS | Length | -0.47 | 0.152 | -0.7 | 0.120 | 0.120 |
| | | dN | NA | NA | NA | -0.097 | 0.800 | 0.795 |
| | Nuc.Rag1 | Total | ***Length*** | ***-0.74*** | ***0.020*** | 0.611 | 0.070 | 0.571 |
| | | dS | ***Length*** | ***-0.63*** | ***0.026*** | ***0.543*** | ***0.036*** | ***0.047*** |
| | | dN | ***Length*** | ***-0.63*** | ***0.026*** | 0.474 | 0.157 | 0.258 |
| Genera | Mito.All | Total | Length | -0.15 | 0.060 | 0.139 | 0.210 | 0.482 |
| | Mito.Coding | dS | ***Length*** | ***-0.1*** | ***<0.001*** | 0.005 | 0.789 | 0.681 |
| | | dN | Length | -0.05 | 0.084 | -0.016 | 0.251 | 0.144 |
| | Nuc.Rag1 | Total | ***Length*** | ***-0.09*** | ***0.001*** | -0.007 | 0.720 | 0.482 |
| | | dS | ***Length*** | ***-0.08*** | ***0.002*** | 0.017 | 0.365 | 0.728 |
| | | dN | NA | NA | NA | 0.026 | 0.313 | 0.313 |

# 1. Validating clade size measures

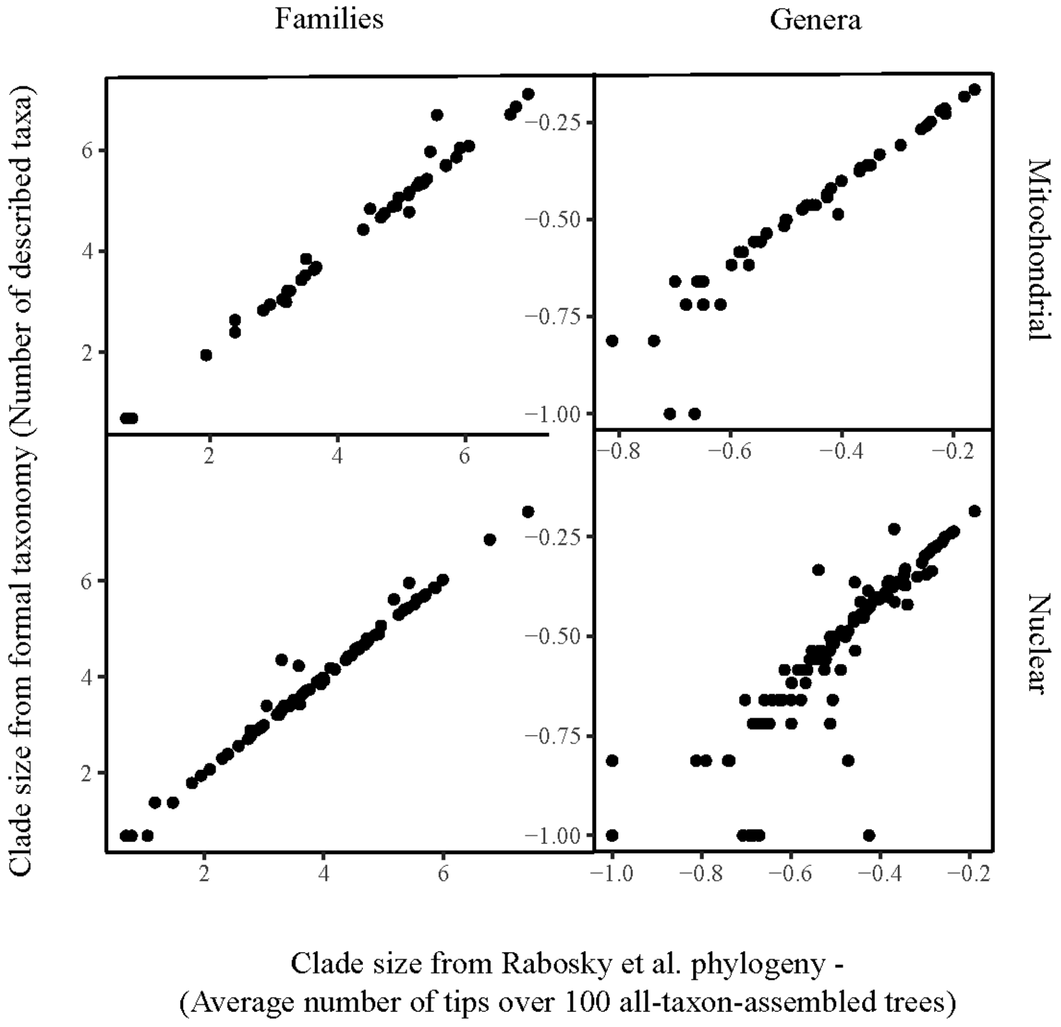


**Figure S1.** Validation of our clade size measure for Total substitution rate (baseml) data. In order to examine the relationship between the diversity of sister clades and their relative substitution rates, we need a measure of the species richness or size of the clade. This is complicated by uncertainty in the positioning of species among genera, and sometimes genera among closely related families. This uncertainty is summarised in 100 all-taxon-assembled phylogenies by Rabosk et al. (2018), in which species without molecular data are distributed randomly within applied taxonomic constraints according to a birth-death process model of diversification. To accommodate this taxonomic uncertainty, we chose to use the average size of a clade across these trees as our diversity measure (Horizontal axis). Since this is a difference from previous sister-pair diversification analyses, which typically use the number of described species in a given taxonomic group as a measure of species richness/clade size (Vertical axis), we use this figure to see whether our method produces substantial differences from this standard method. The formal taxonomic count is the sum of FishBase described species for each genus descending from the common ancestor of that sister clade in the phylogeny of Rabosky et al. (2018). Some genera are not represented in the backbone tree, meaning that relying on formal taxonomy misses these genera, leading to very small clade sizes. This is remedied by our methodology Otherwise the two measures of species richness/clade size are highly correlated.

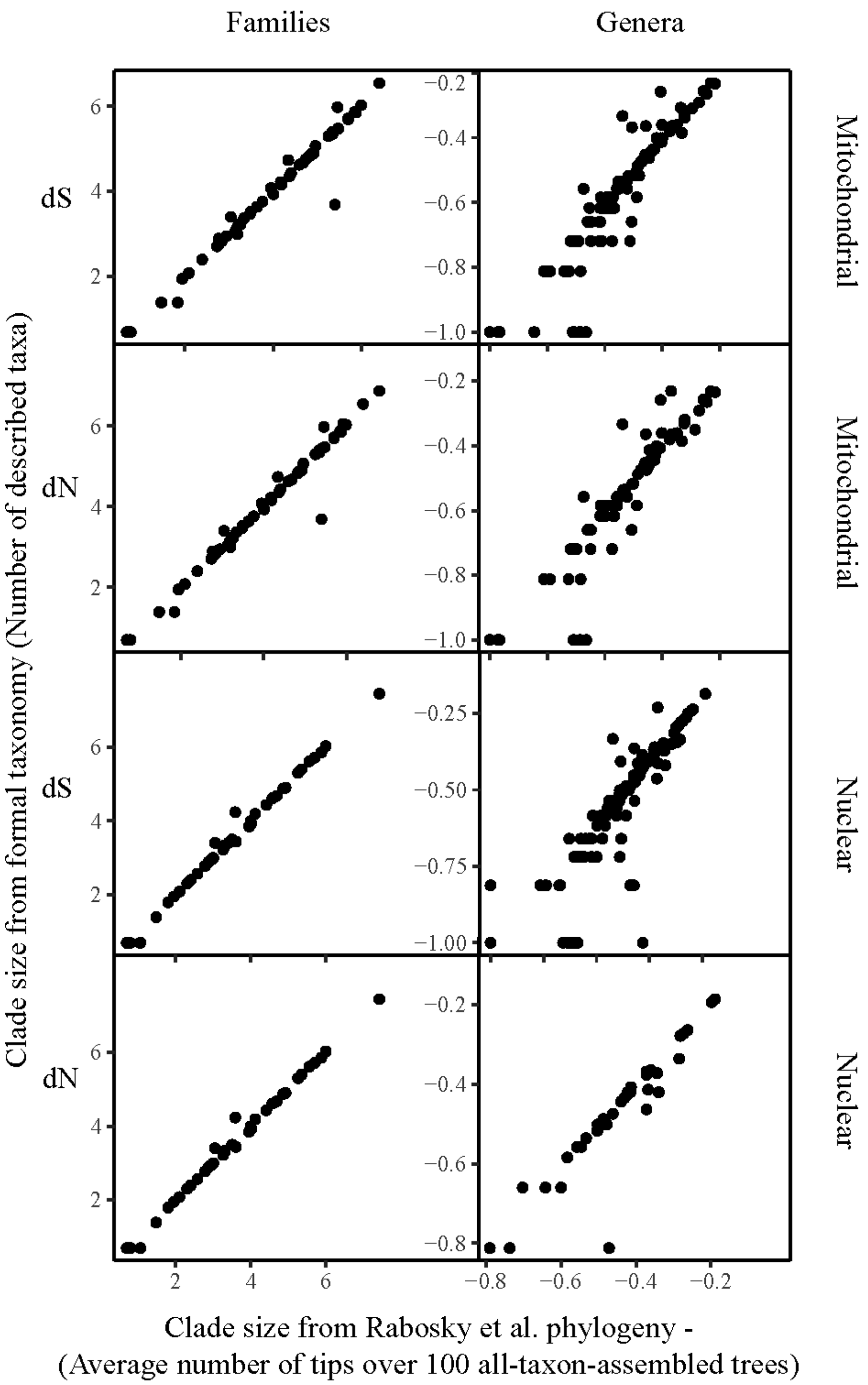


**Figure S2.** Validation of our clade size measure for dN and dS substitution rate (codeml) data. Our measure was taken to be the average size of the clade across 100 all-taxon assembled trees, accounting for possible uncertainty in the placement of species without molecular data. These averages are plotted against a count derived from the FishBase taxonomy (horizontal axis). This count is the sum of FishBase described species for each genus descending from the common ancestor of that sister clade in the phylogeny of Rabosky et al. (2018). Some genera are not represented in the backbone tree, leading to a greatly increased clade size when averages are taken over the all-taxon-assembled phylogeny. Otherwise clade size are highly correlated between the two methods.

# 2. Alignment Length Distributions

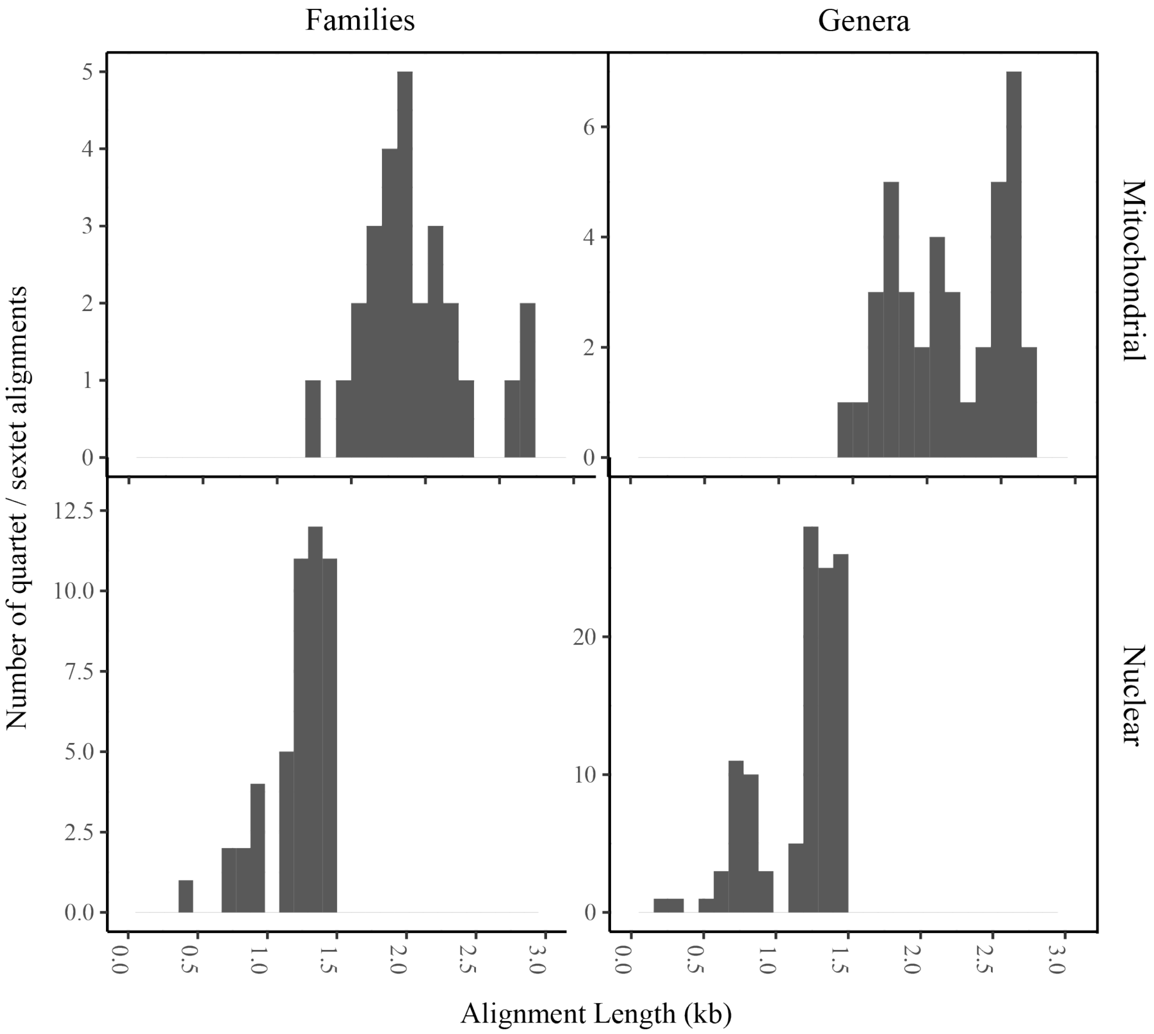


**Figure S3.** Histogram of quartet alignment lengths in kilobases (kb) used to infer Total substitution rates (baseml) for the Full data sets.

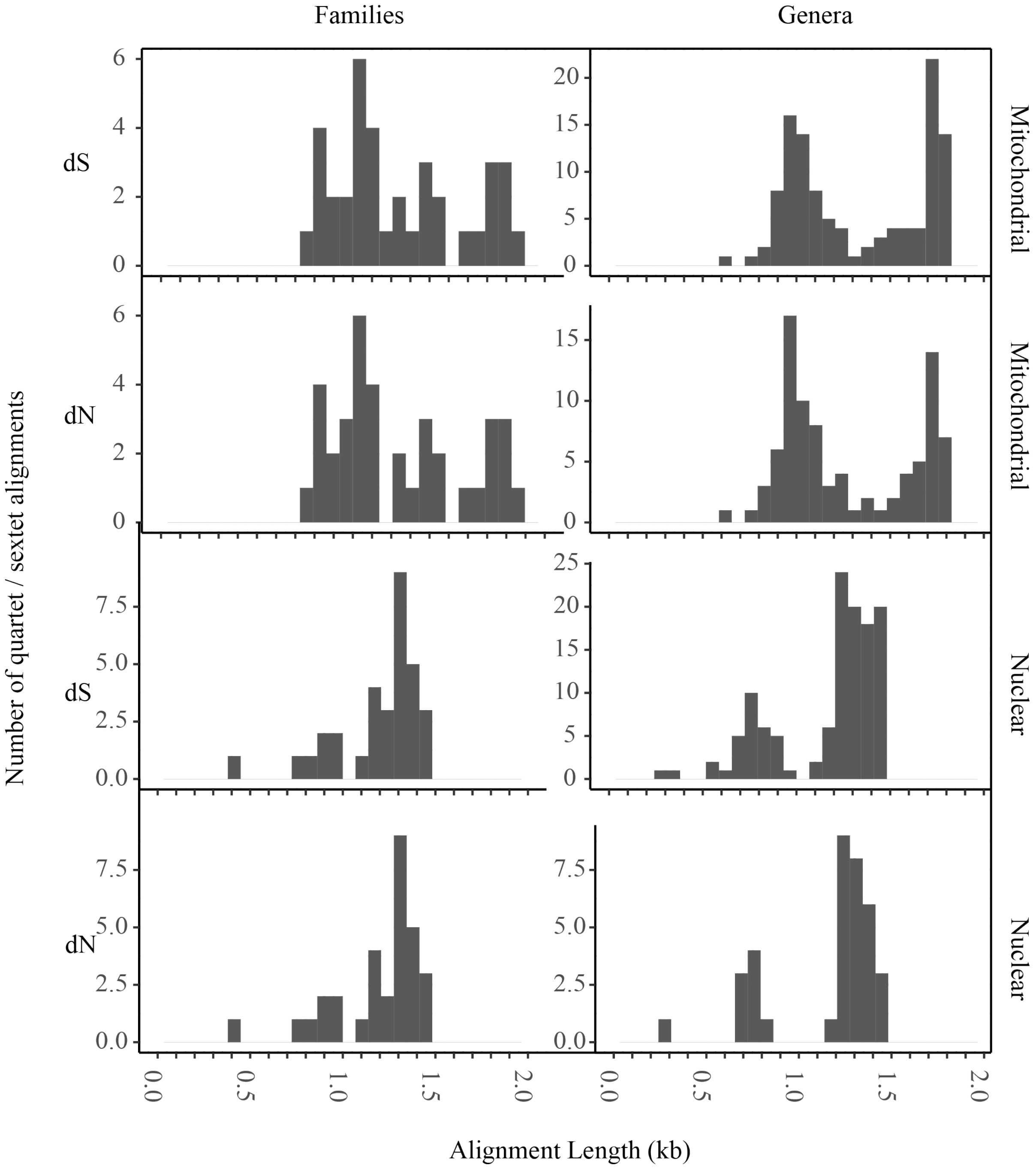


**Figure S4.** Histogram of quartet alignment lengths in kilobases (kb) used to infer dS or dN (codeml) substitution rates for Full data sets.

# 3. Pair Age Distributions

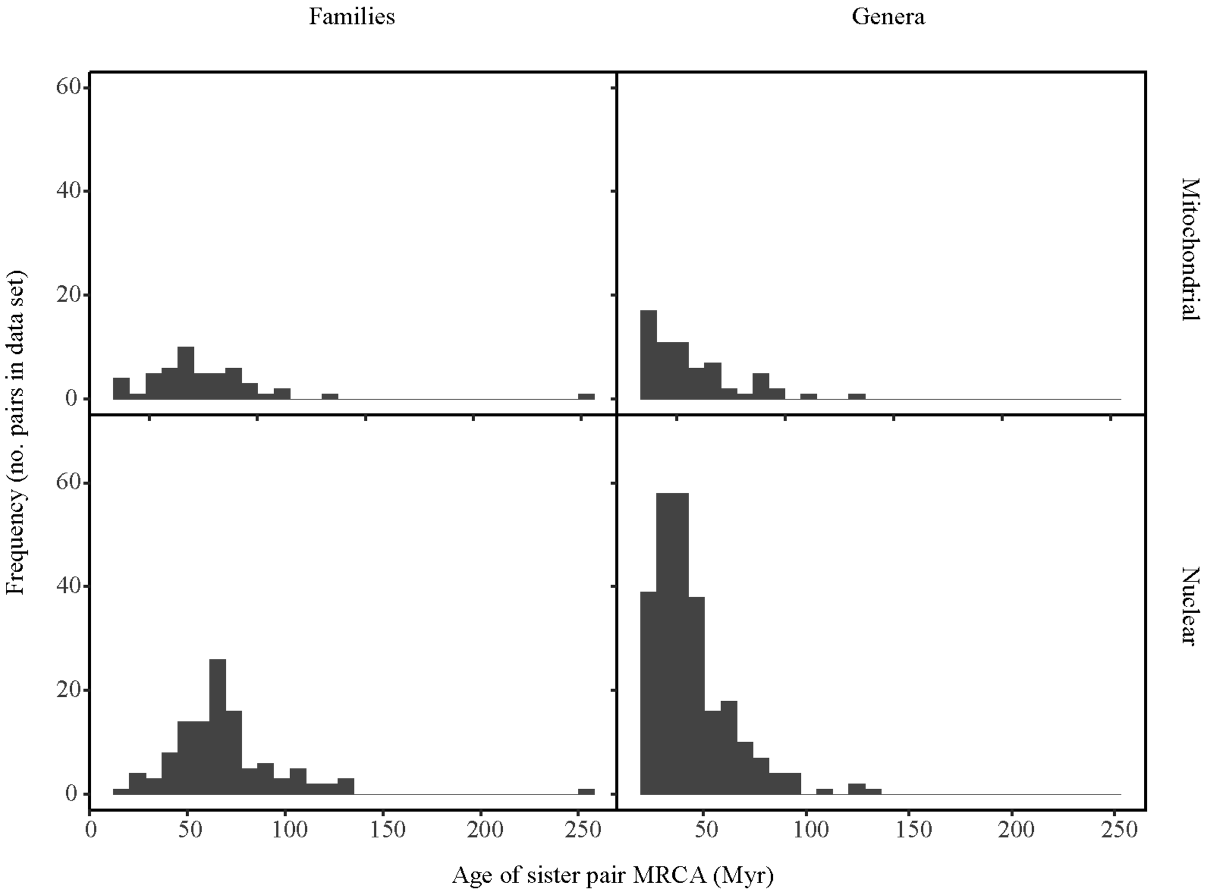


**Fig. S5.** Distribution of the age of the most recent common ancestor (MRCA) of the sister pairs forming the Full data sets for analysing Total substitution rates.Age estimates are molecular dates taken from Rabosky et al. (2018) in millions of years (Myr).

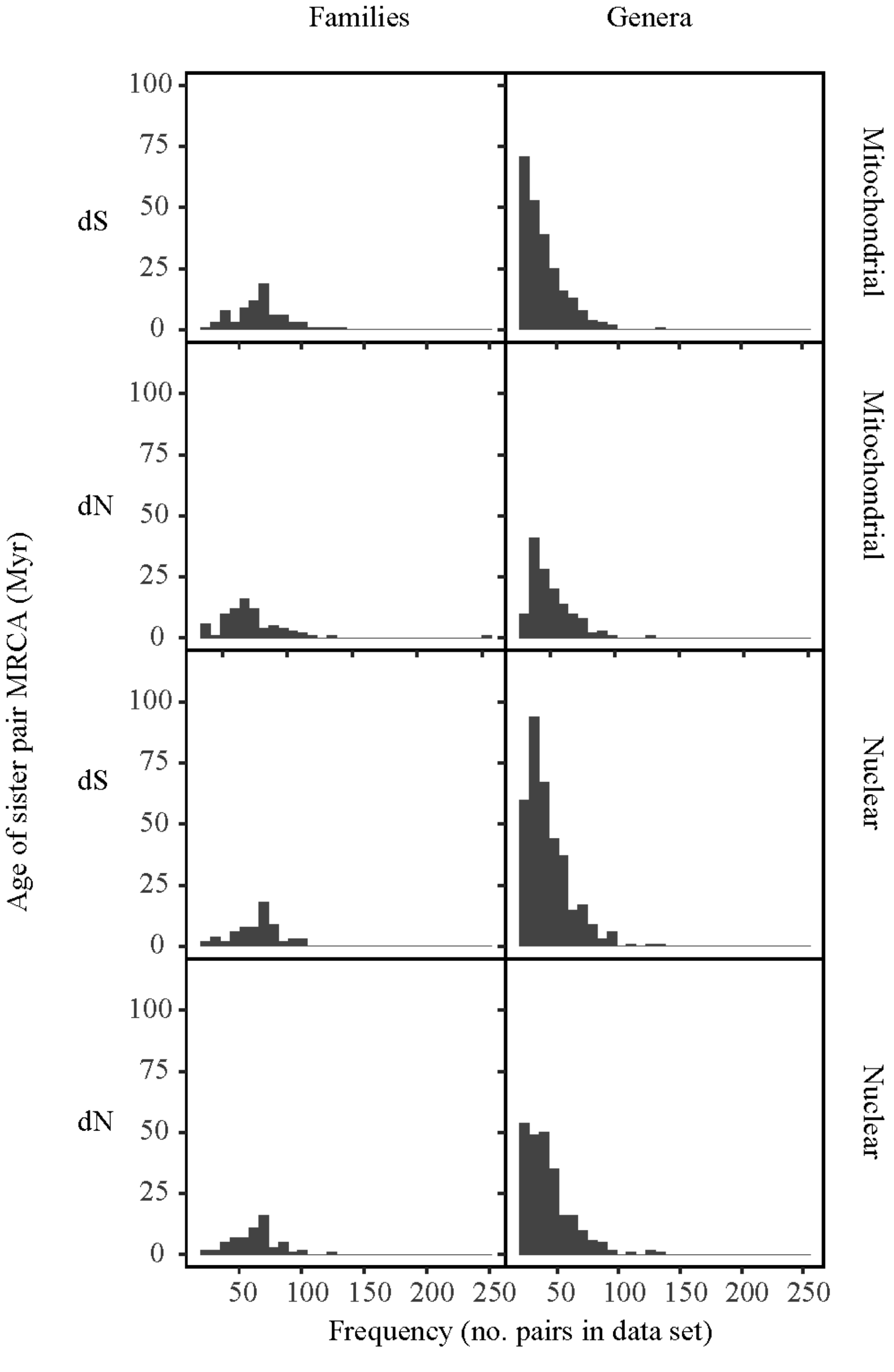


**Fig. S6.** Distribution of the age of the most recent common ancestor (MRCA) of the sister pairs forming the Full data sets for analysing dS and dN substitution rates. Age estimates are molecular dates taken from Rabosky et al. (2018) in millions of years (Myr).

# 4. Relationship between Clade Size and Substitution Rate Contrasts

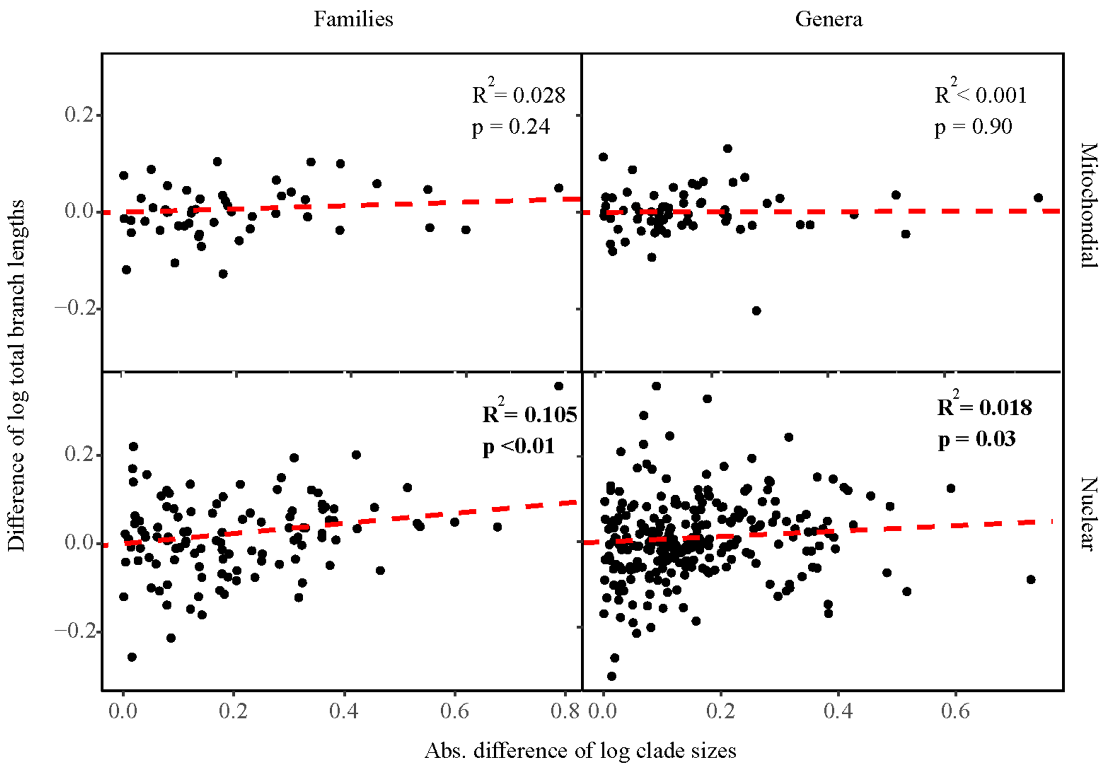


**Fig. S7. Total substitution rates, Full data set, no additional validation/transformation.** Sister pair contrasts in Total substitution rates plotted against contrasts in clade sizes, with no checking for monophyly or extra validation/transformation steps. Data are shown for mitochondrial and nuclear sequences and family- and genus-level data sets. All variables are log-transformed and standardised by dividing by the square root of the age of the pair MRCA. Substitution rates are transformed by logarithms at all levels. Contrasts are standardised by the square root of pair age. The dotted line shows the least squares regression line forced through the origin. Bold text indicates $p < 0.05$

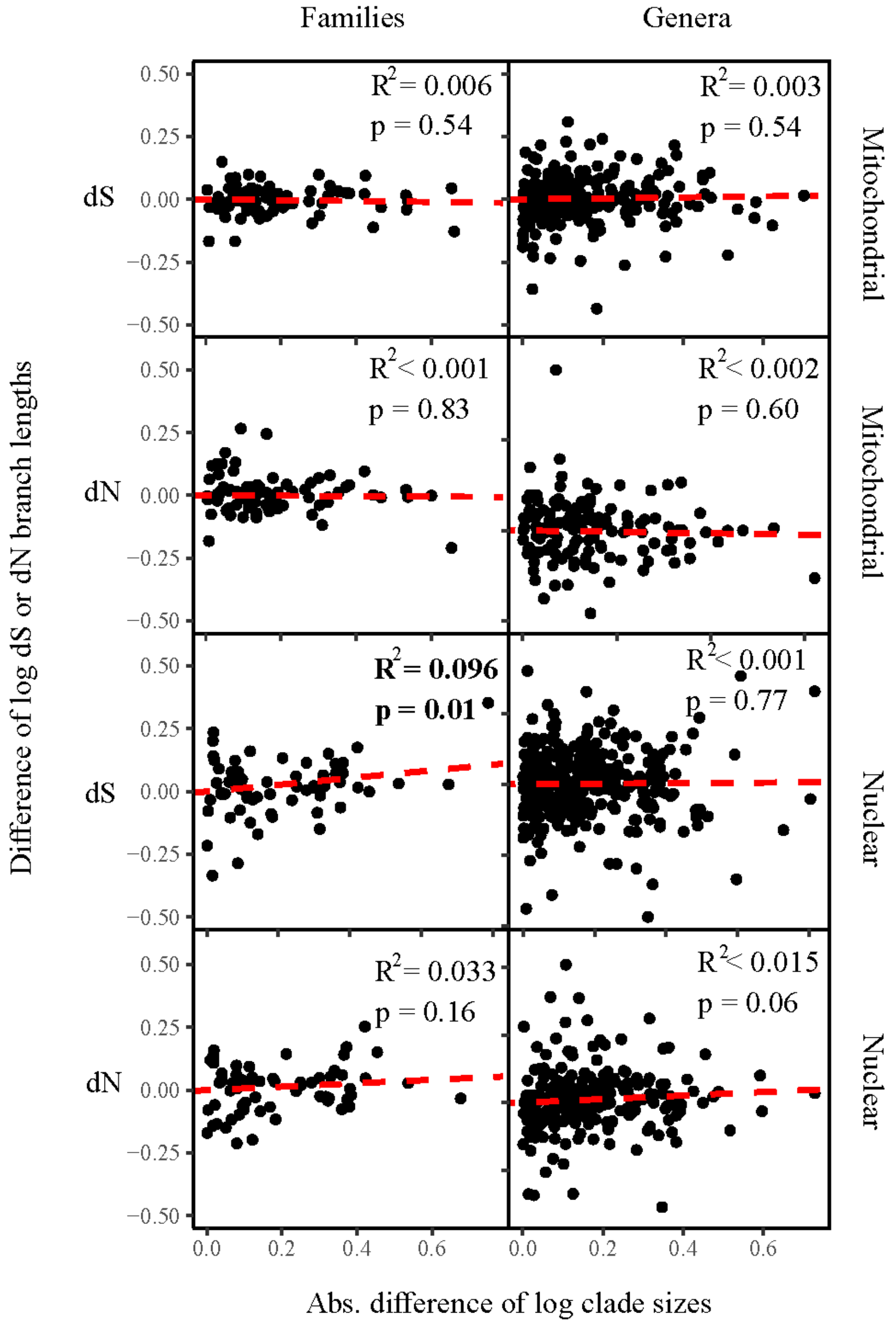


**Fig. S8. dS/dN substitution rates, Full data set, no additional validation/transformation.** Sister pair contrasts in synonymous (dS) or nonsynonymous (dN) substitution rates plotted against contrasts in clade sizes, with no checking for monophyly or extra validation/transformation steps (Supplement 0). Data are shown for mitochondrial and nuclear sequences and family- and genus-level data sets. All variables are log-transformed and standardised by dividing by the square root of the age of the pair MRCA. Substitution rates are transformed by logarithms at all levels. Contrasts are standardised by the square root of pair age. The dotted line shows the least squares regression line forced through the origin. Bold text indicates $p < 0.05$.

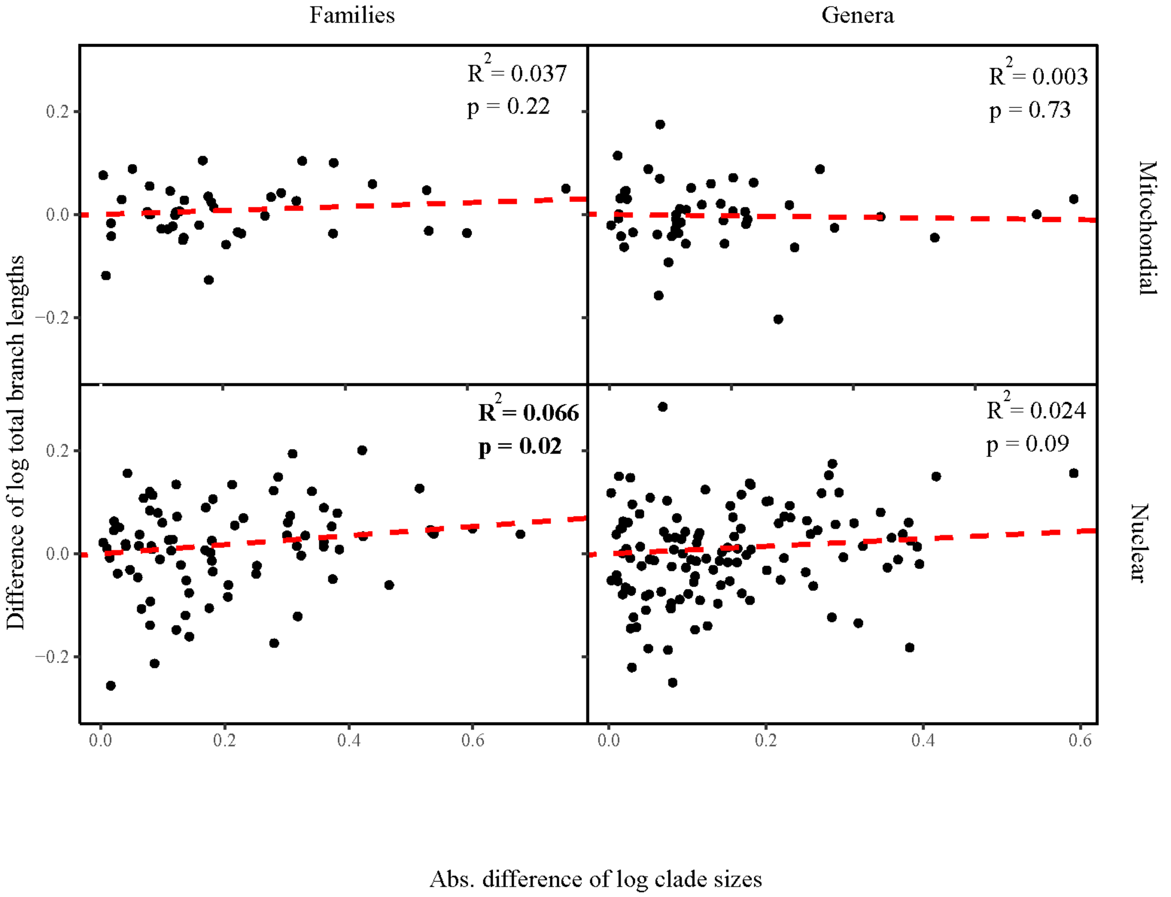


**Fig. S9. Total substitution rates, Reduced data set, no additional validation/transformation.** Sister pair contrasts in Total substitution rates plotted against contrasts in clade sizes, with checking for mutual monophyly of clades but without extra validation/transformation steps. The monophyly checking step removes pairs where one or more genera are divided among both sides of the clade in 80 or more of the 100 all-taxon assembled trees in the Rabosky et al. (2018) data set. Data are shown for mitochondrial and nuclear sequences and family- and genus-level data sets. All variables are log-transformed and standardised by dividing by the square root of the age of the pair MRCA. Substitution rates are transformed by logarithms at all levels. Contrasts are standardised by the square root of pair age. The dotted line shows the least squares regression line forced through the origin. Bold text indicates $p < 0.05$.

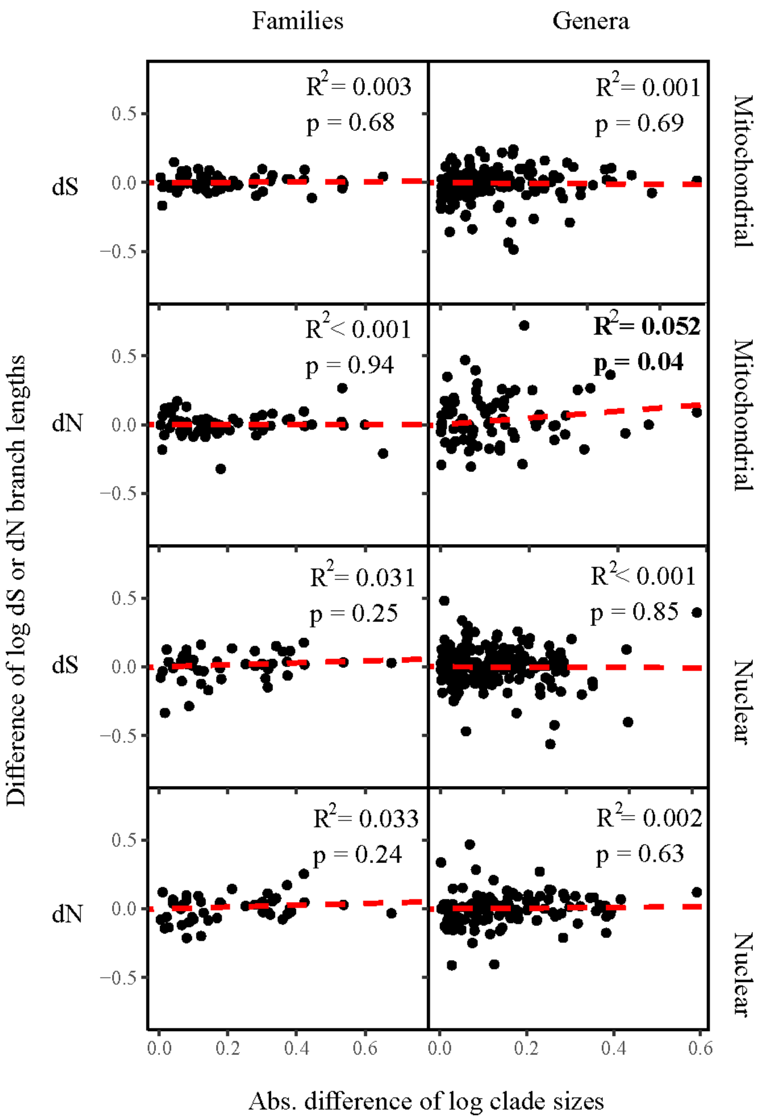


**Fig. S10. dS/dN substitution rates, Reduced data set, no additional validation/transformation.** Sister pair contrasts in synonymous (dS) or nonsynonymous (dN) substitution rates plotted against contrasts in clade sizes, with checking for mutual monophyly of clades but without extra validation/transformation steps. The monophyly checking step removes pairs where one or more genera are divided among both sides of the clade in 80 or more of the 100 all-taxon assembled trees in the Rabosky et al. (2018) data set. Data are shown for mitochondrial and nuclear sequences and family- and genus-level data sets. All variables are log-transformed and standardised by dividing by the square root of the age of the pair MRCA. Substitution rates are transformed by logarithms at all levels. Contrasts are standardised by the square root of pair age. The dotted line shows the least squares regression line forced through the origin. Bold text indicates $p < 0.05$.

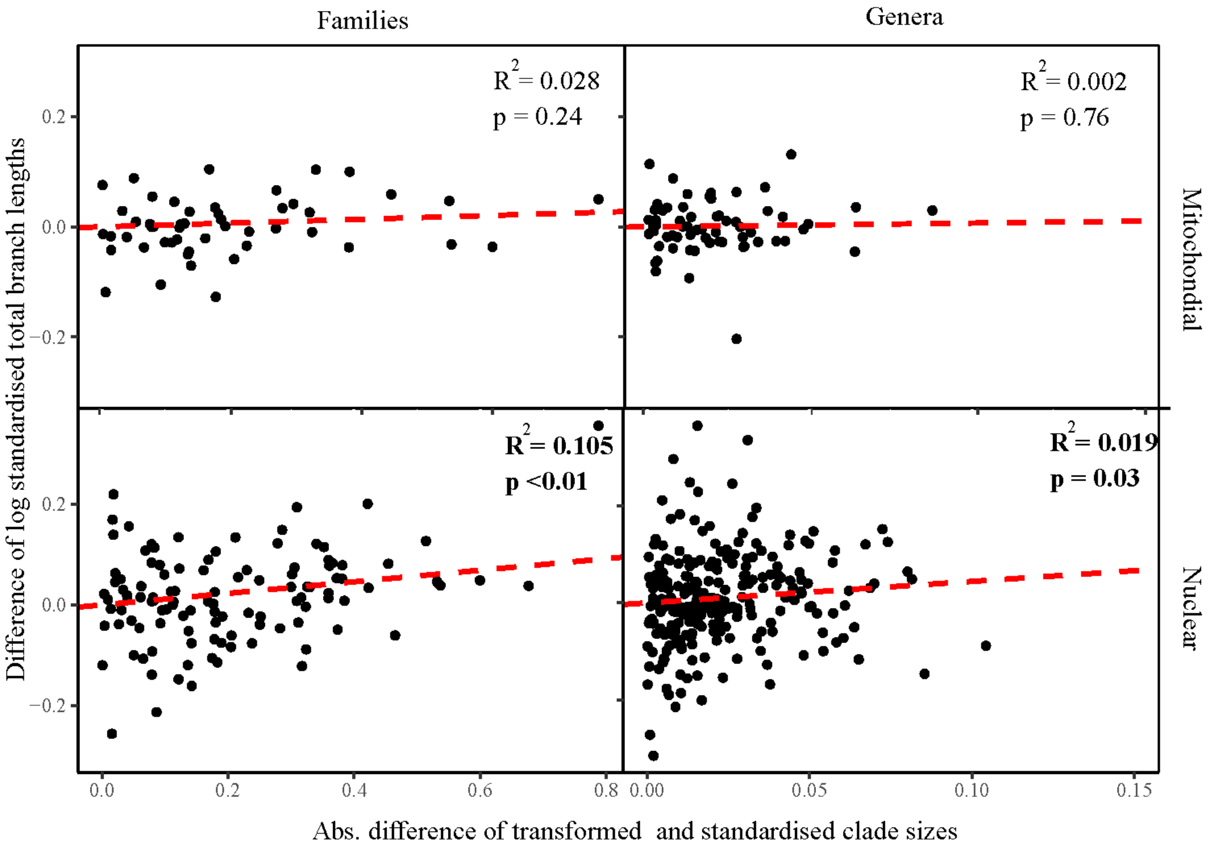


**Fig. S11. Total substitution rates, Full data set, with additional validation/transformation.** Sister pair contrasts in Total substitution rates plotted against contrasts in clade sizes, with no checking for monophyly. Additional validation steps and transformations were performed as detailed in Supplement 0. Clade sizes are transformed by logarithm (family-level) or by $N^* = -1/N^{0.3}$ (genus level). Substitution rates are transformed by logarithms at all levels. Contrasts are standardised by the square root of pair age. Data are shown for mitochondrial and nuclear sequences and family- and genus-level data sets. The dotted line shows the least squares regression line forced through the origin. Bold text indicates $p < 0.05$

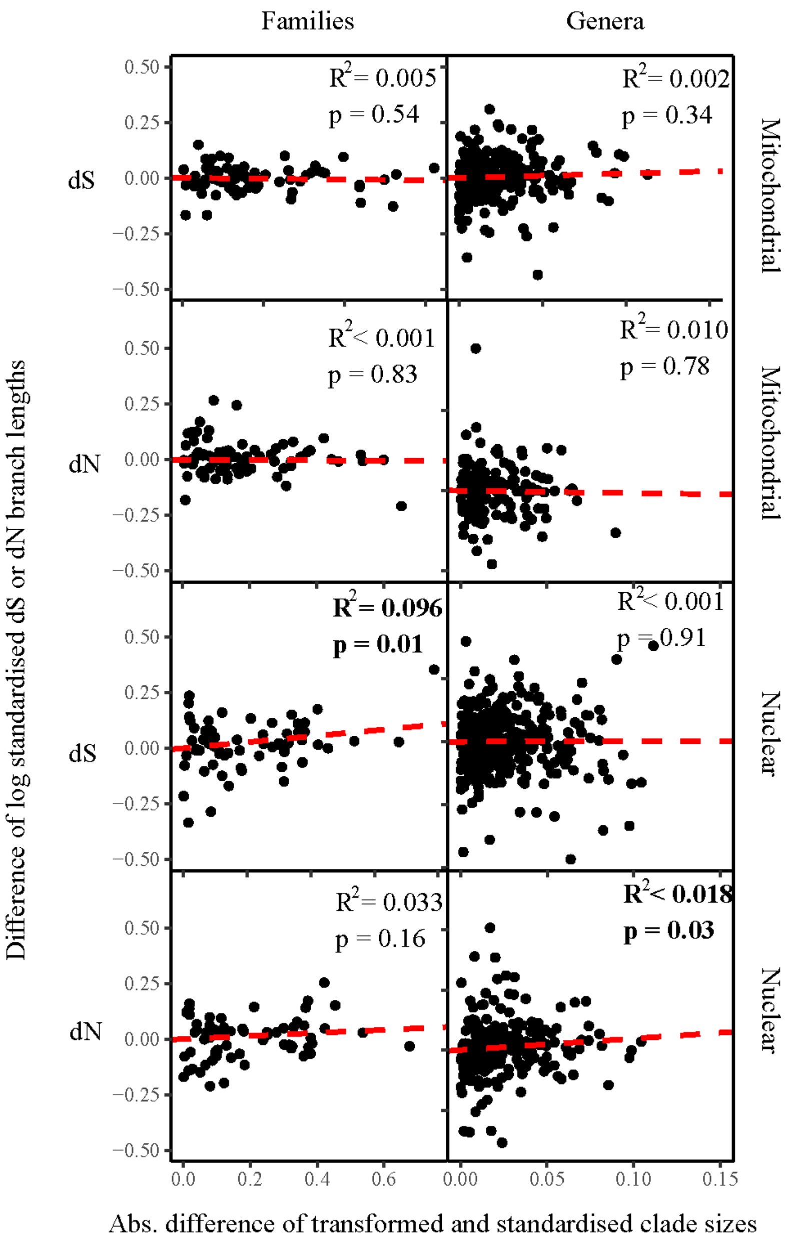


**Fig. S12. dS/dN substitution rates, Full data set, with additional validation/transformation.** Sister pair contrasts in synonymous (ds) and nonsynonymous (dN) substitution rates plotted against contrasts in clade sizes, with no checking for monophyly. Additional validation steps and transformations were performed as detailed in Supplement 0. Clade sizes are transformed by logarithm (family-level) or by $N^* = -1/N^{0.3}$ (genus level). Substitution rates are transformed by logarithms at all levels. Contrasts are standardised by the square root of pair age. Data are shown for mitochondrial and nuclear sequences and family- and genus-level data sets. The dotted line shows the least squares regression line forced through the origin. Bold text indicates $p < 0.05$

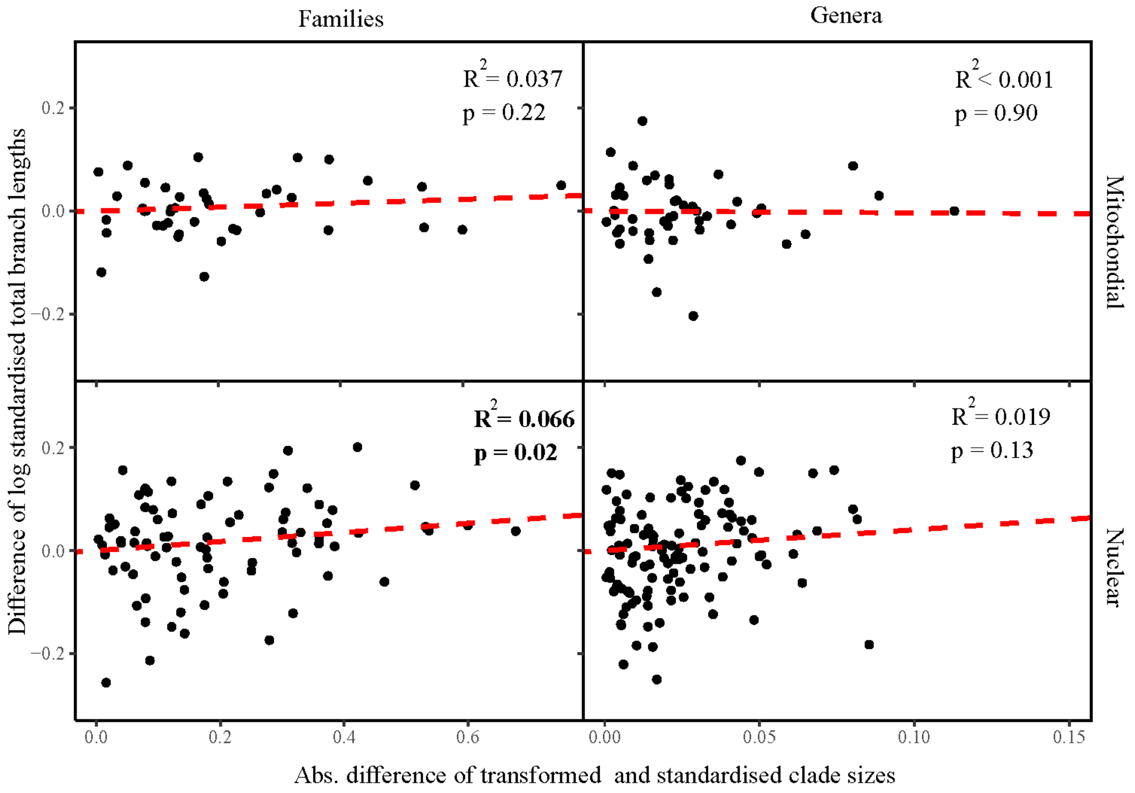


**Fig. S13. Total substitution rates, Reduced data set, with additional validation/transformation.** Sister pair contrasts in Total substitution rates plotted against contrasts in clade sizes, with checking for mutual monophyly of sister pair clades. Additional validation steps and transformations were performed as detailed in Supplement 0. Clade sizes are transformed by logarithm (family-level) or by $N^* = -1/N^{0.3}$ (genus level). Substitution rates are transformed by logarithms at all levels. Contrasts are standardised by the square root of pair age. Data are shown for mitochondrial and nuclear sequences and family- and genus-level data sets. The dotted line shows the least squares regression line forced through the origin. Bold text indicates $p < 0.05$

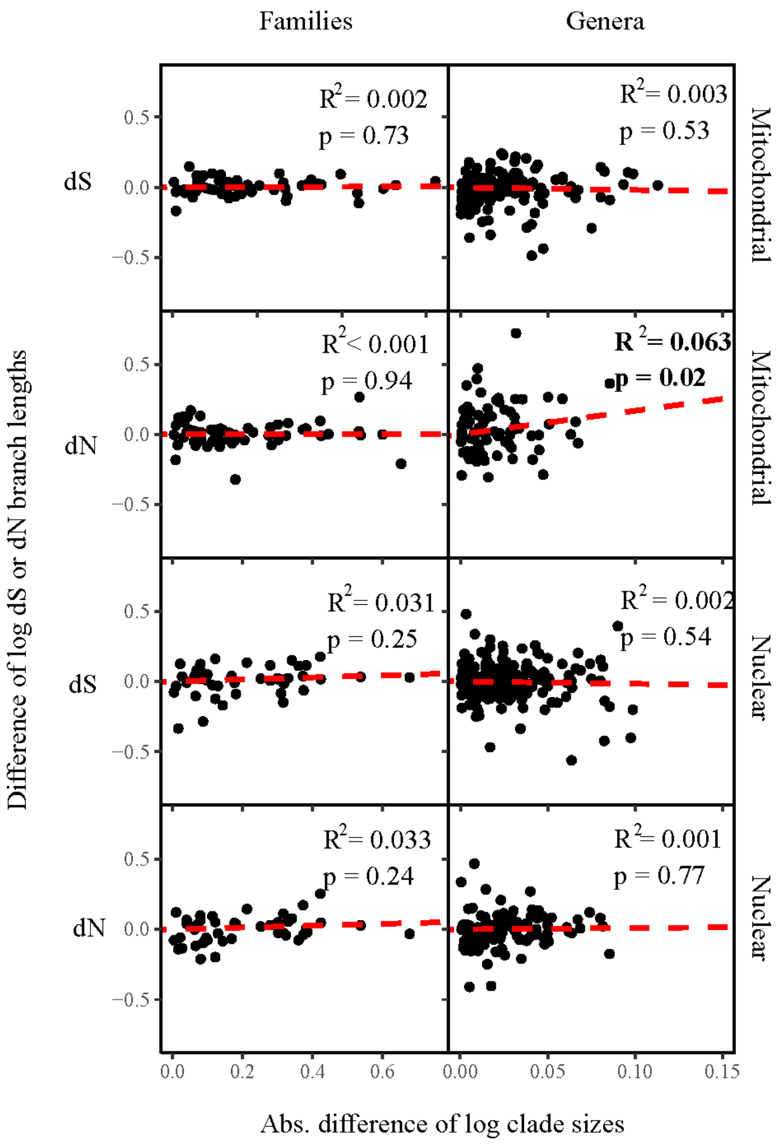


**Fig. S14. dS/dN substitution rates, Reduced data set, with additional validation/transformation.** Sister pair contrasts in synonymous (ds) and nonsynonymous (dN) substitution rates plotted against contrasts in clade sizes, with checking for mutual monophyly of sister pair clades. The monophyly checking step removes pairs where one or more genera are divided among both sides of the clade in 80 or more of the 100 all-taxon assembled trees in the Rabosky et al. (2018) data set. Additional validation steps and transformations were performed as detailed in Supplement 0. Clade sizes are transformed by logarithm (family-level) or by $N^* = -1/N^{0.3}$ (genus level). Substitution rates are transformed by logarithms at all levels. Contrasts are standardised by the square root of pair age. Data are shown for mitochondrial and nuclear sequences and family- and genus-level data sets. The dotted line shows the least squares regression line forced through the origin. Bold text indicates $p < 0.05$

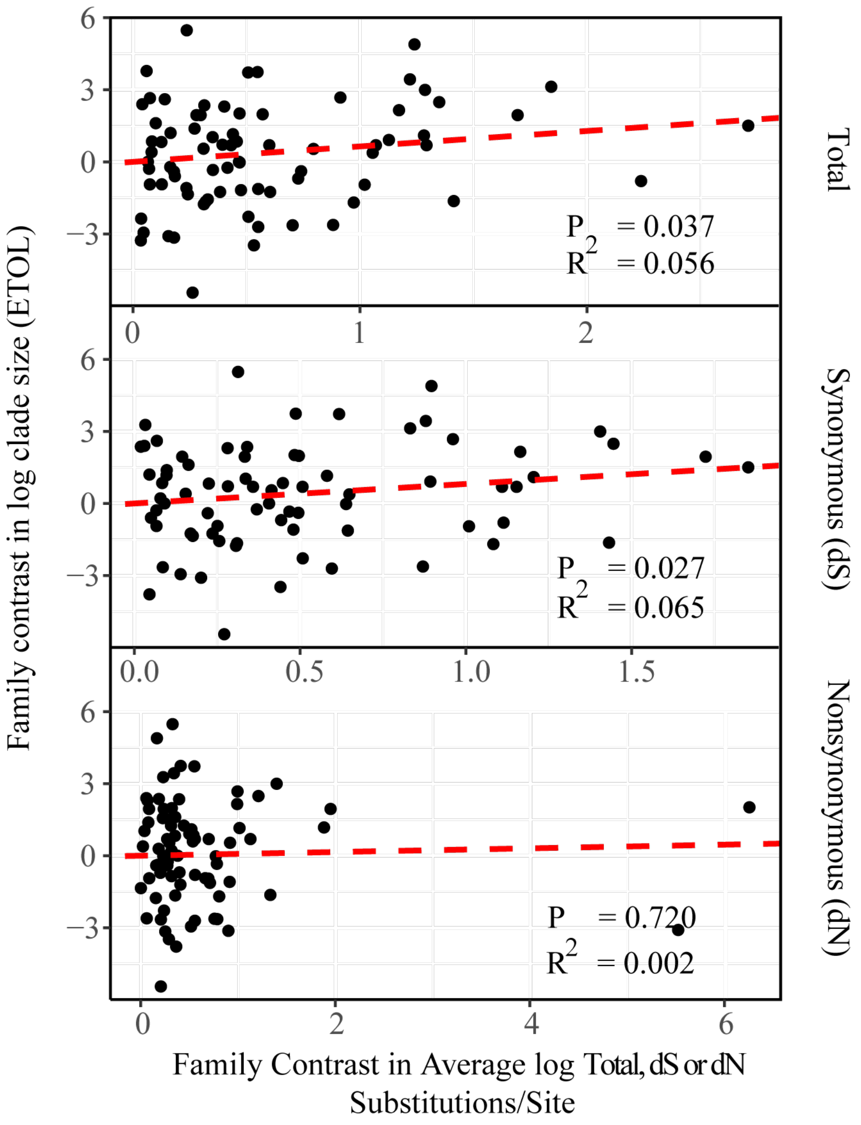


**Fig. S15. Euteleost Tree Of Life (ETOL) data.** Sister pair contrasts in Total, dS and dN substitution rates plotted against contrasts in clade sizes, using the phylogeny and molecular data set of Betancur-R. et al (2013). Only monophyletic clades are used. No additional validation steps or transformations were performed. Clade sizes and substitution rates are transformed by logarithms at all levels. Contrasts are standardised by the square root of pair age. Data are shown for mitochondrial and nuclear sequences and family- and genus-level data sets. The dotted line shows the least squares regression line forced through the origin. Bold text indicates $p < 0.05$.

# 5. Validation checks – Homoskedasticity

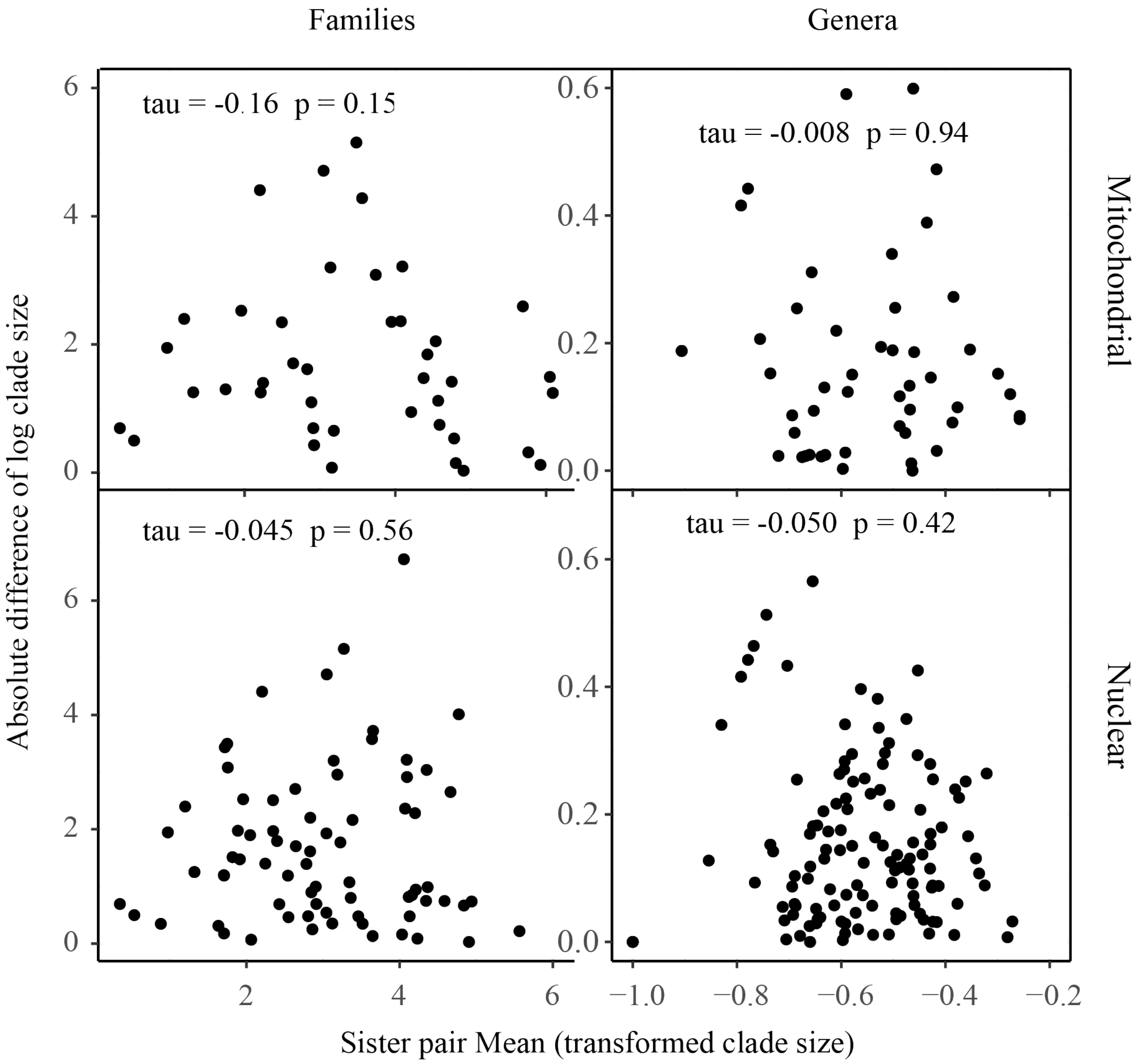


**Figure S16.** Full dataset, Total substitution rates (baseml) - test for heteroscedasticity of sister pair log clade size contrasts. Data is shown for mitochondrial and nuclear sequences at the family and genus taxonomic levels. Absolute contrasts (vertical axis) are plotted against the sister pair mean of the clade sizes in each sister clade within the pair (horizontal axis). Clade sizes are then transformed so that positive relationship remains (Kendall rank-correlation test, $p > 0.05$). For family-level data a log transformation is used, while for genus-level data the transformation applied is $N^* = -1/N^{0.3}$.

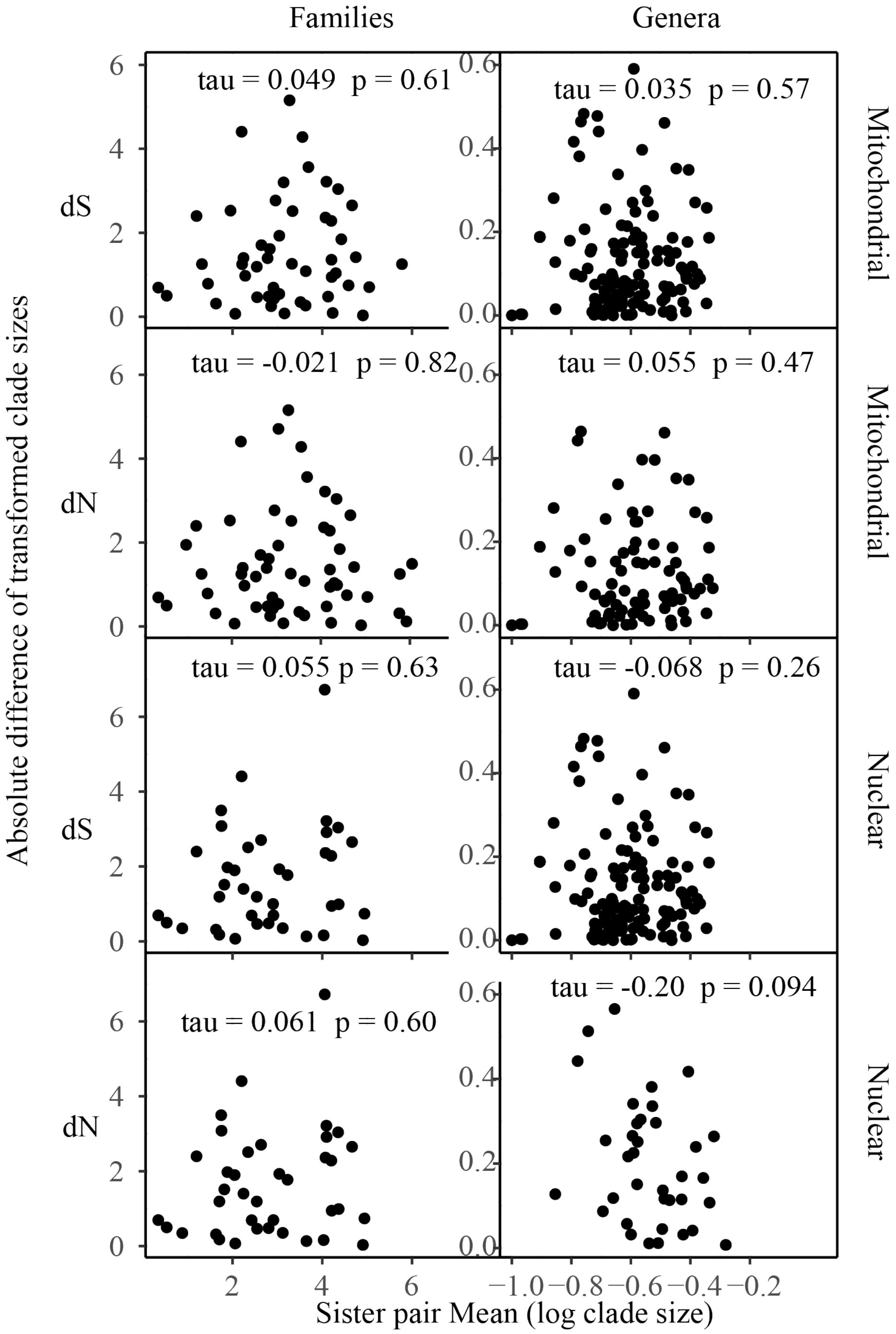


**Figure S17.** Full data sets, dS or dN (codeml) substitution rates - test for heteroscedasticity of sister pair log clade size. Data is shown for mitochondrial and nuclear sequences at the family and genus taxonomic levels. Absolute contrasts (vertical axis) are plotted against the sister pair mean of the clade sizes in each sister clade within the pair (horizontal axis). Clade sizes are then transformed so that positive relationship remains (Kendall rank-correlation test, $p > 0.05$). For family-level data a log transformation is used, while for genus-level data the transformation applied is $N^* = -1/N^{0.3}$.

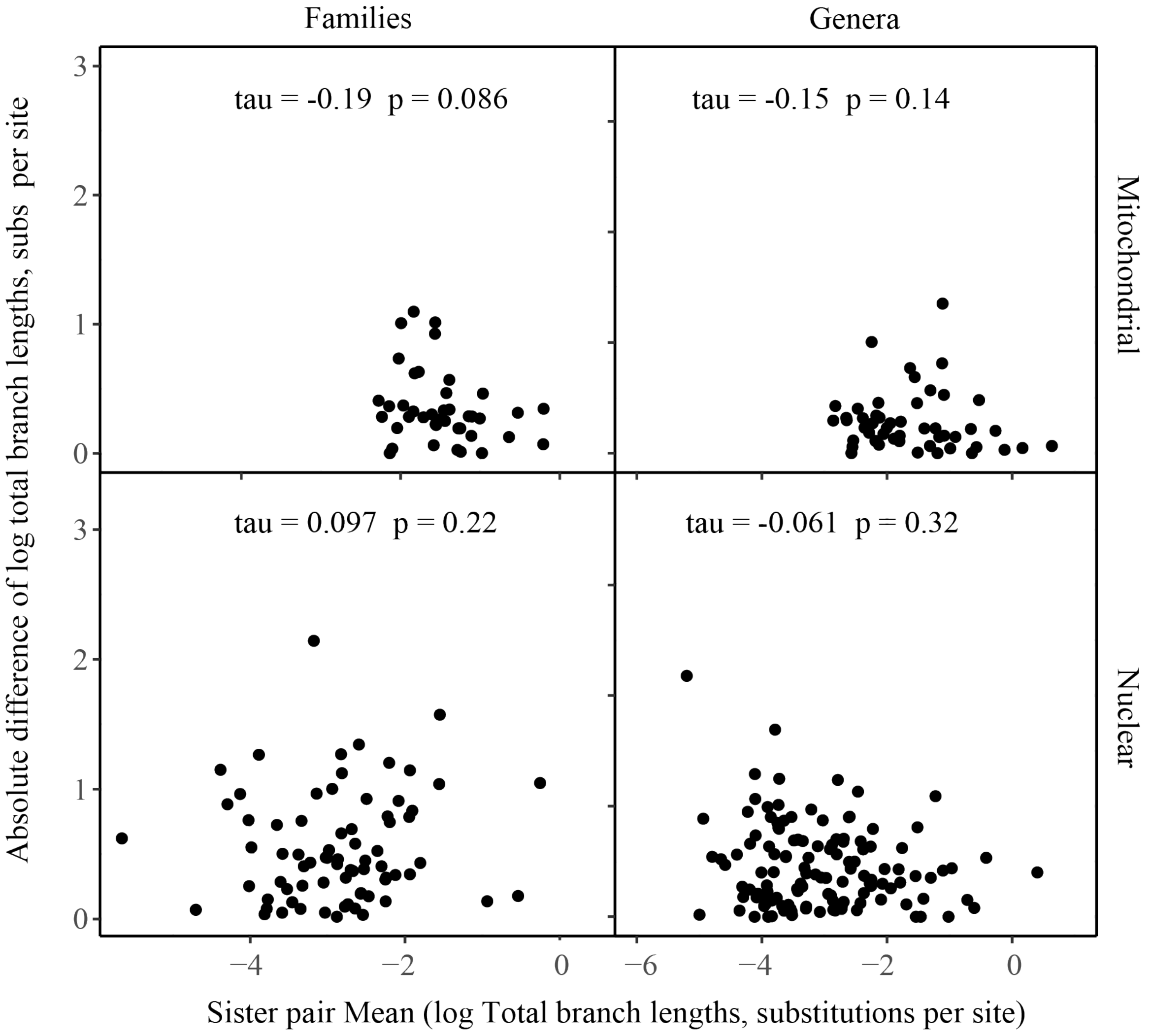


**Figure S18.** Full data sets, Total substitution rates (baseml) - test for heteroscedasticity of sister pair log branch length contrasts. Data is shown for mitochondrial and nuclear sequences at the family and genus taxonomic levels. Absolute contrasts (vertical axis) are plotted against the sister pair mean of the log substitution rates in each sister clade within the pair (horizontal axis). Substitution rates are then transformed so that no positive relationship remains (Kendall rank-correlation test, $p > 0.05$). The log transformation is found to be adequate for all data sets.

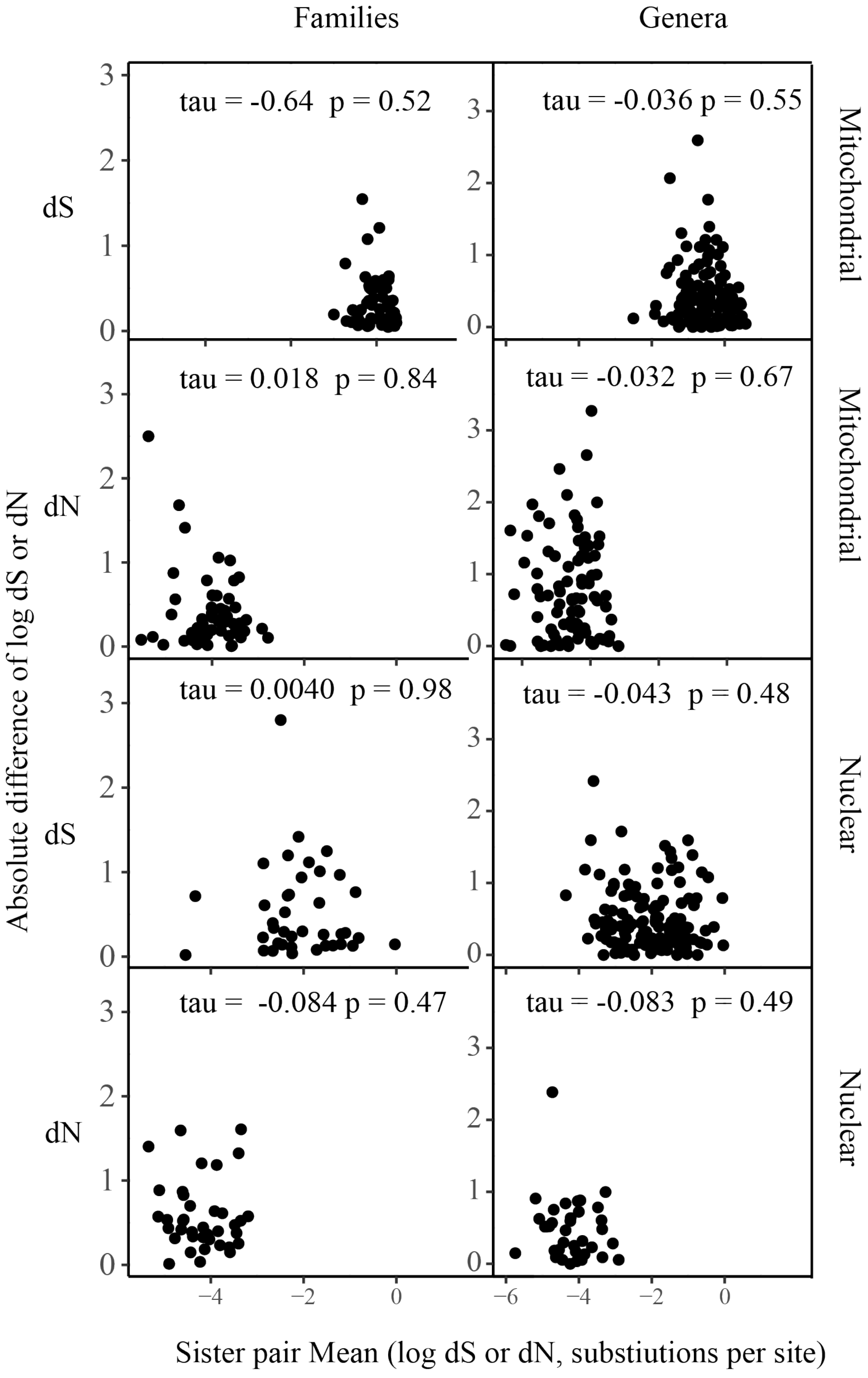


**Figure S19.** Full data sets, dS or dN (codeml) substitution rates - test for heteroscedasticity of sister pair log branch length contrasts. Data is shown for mitochondrial and nuclear sequences at the family and genus taxonomic levels. Substitution rates are for non-synonymous (dN) or synonymous (dS) substitutions. Absolute contrasts (vertical axis) are plotted against the sister pair mean of the log substitution rates in each sister clade within the pair (horizontal axis). Substitution rates are then transformed so that no positive relationship remains (Kendall rank-correlation test, $p > 0.05$). The log transformation is found to be adequate for all data sets.

# 6. Validation checks – Relationship of Variance with Pair Age

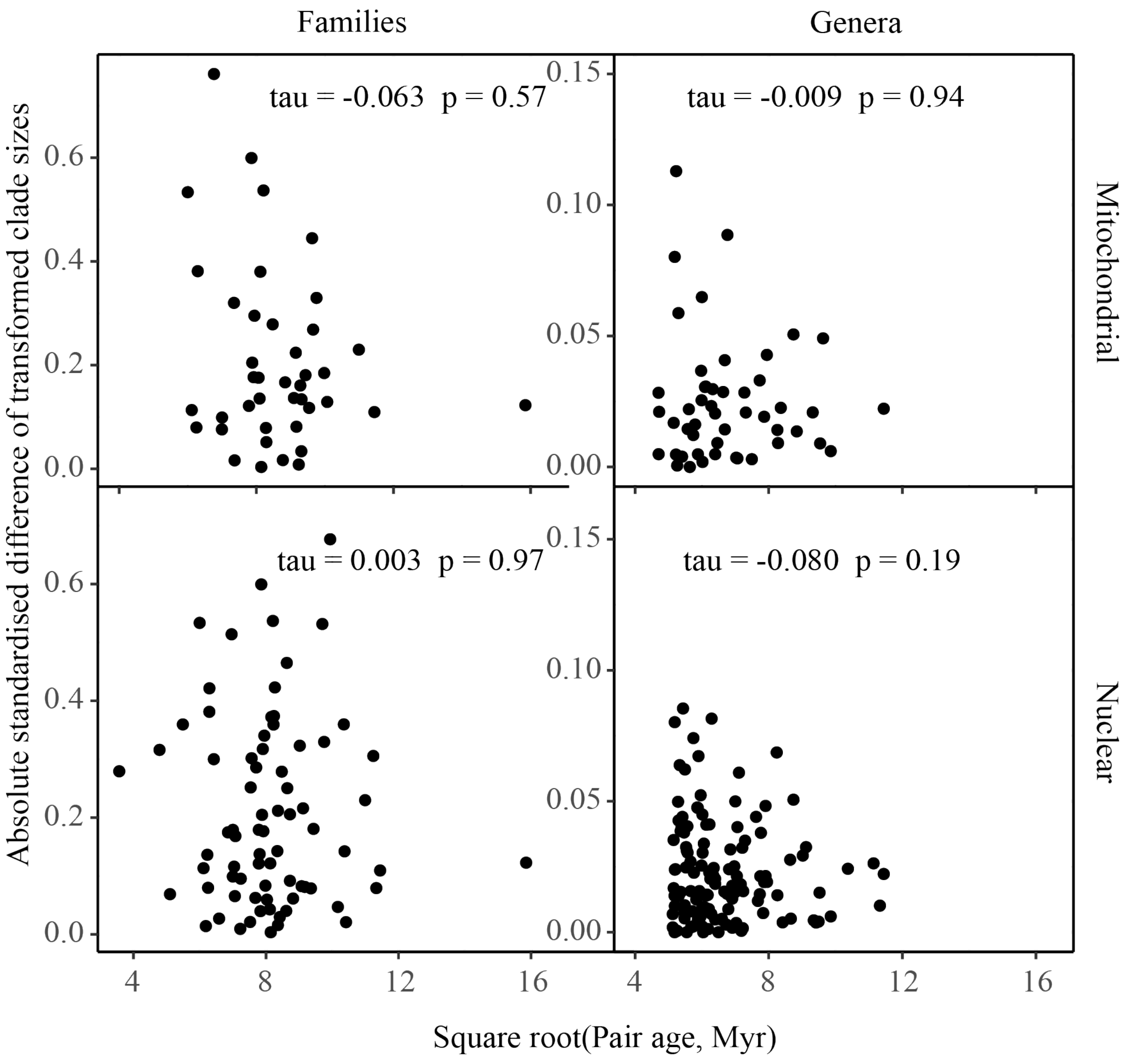


**Figure S20.** Full data sets, Total substitution rates (baseml) - tests for nonhomogeneity of variance in transformed sister pair species contrasts due to the age of sister pairs. Data is shown for mitochondrial and nuclear sequences at the family and genus taxonomic levels. Vertical axis is the absolute values of the contrasts standardised by the square root of pair age in millions of years (Myr) and plotted against the standardisation factor (Sqrt pair age). For family-level data a log transformation is used, while for genus-level data the transformation applied is $N^* = -1/N^{0.3}$. We then test for any remaining correlation (Kendall rank-correlation test, p > 0.05). This test showed that standardisation by the square root of sister pair age is adequate.

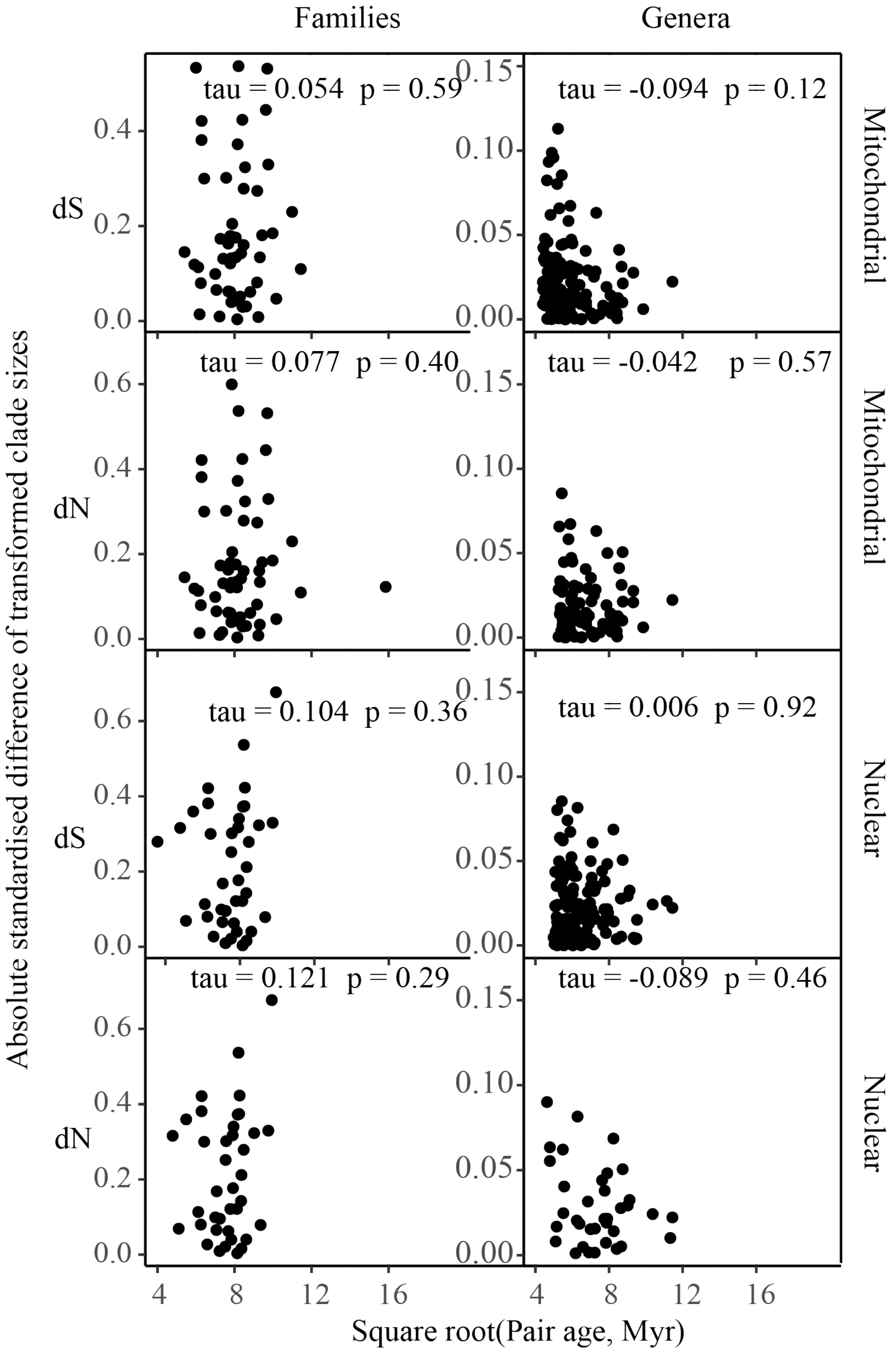


**Figure S21.** Full data sets, dS or dN (codeml) substitution rates - tests for nonhomogeneity of variance in transformed sister pair species contrasts due to the age of sister pairs. Data is shown for mitochondrial and nuclear sequences at the family and genus taxonomic levels. Vertical axis is the absolute values of the contrasts standardised by the square root of pair age in millions of years (Myr) and plotted against the standardisation factor (Sqrt pair age). For family-level data a log transformation is used, while for genus-level data the transformation applied is $N^* = -1/N^{0.3}$. We then test for any remaining correlation (Kendall rank-correlation test, $p > 0.05$). This test showed that standardisation by the square root of sister pair age is adequate.

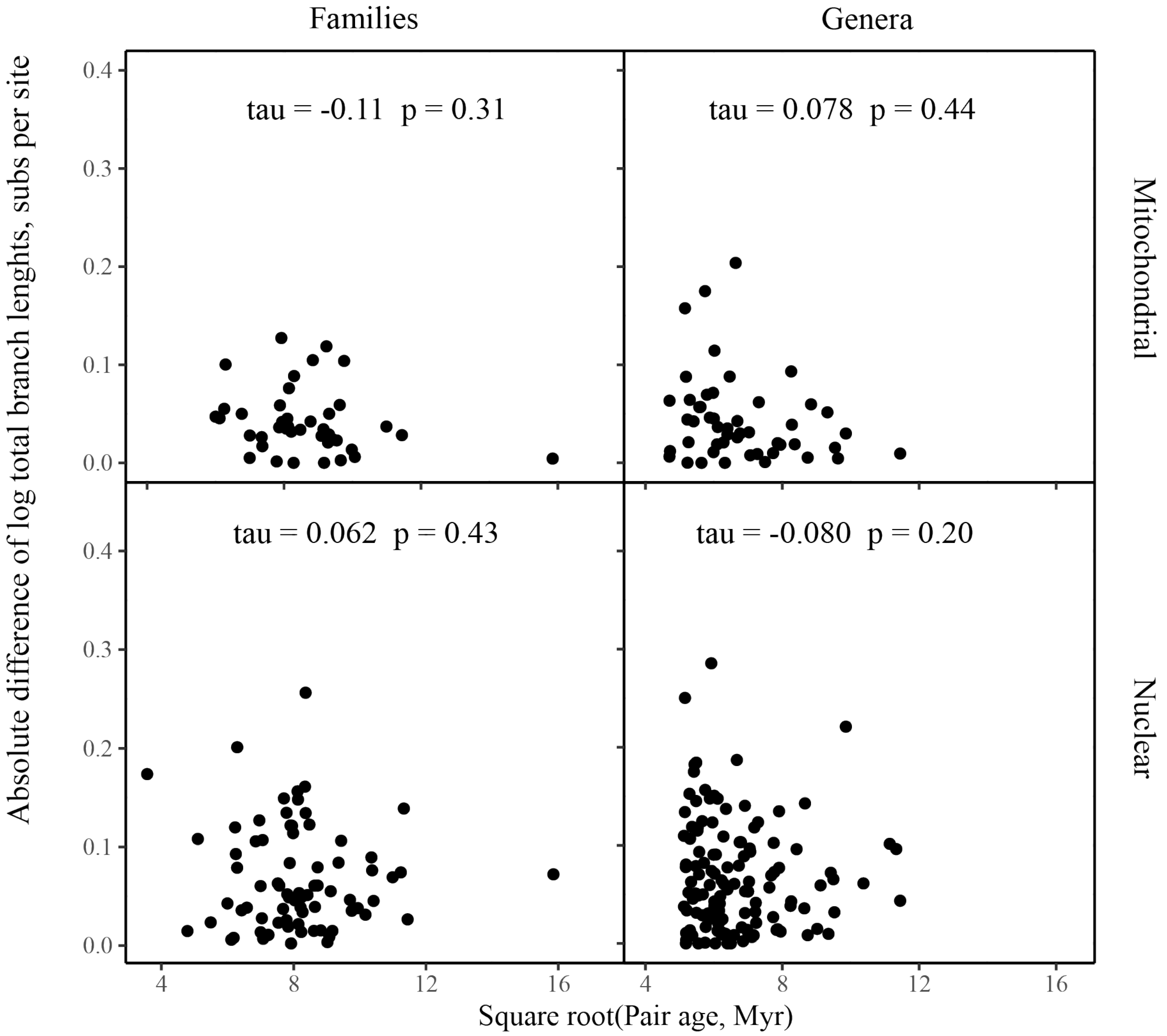


**Figure S22.** Full data sets, Total branch length (baseml) substitution rates - Tests for an effect of pair age on the variance of branch length estimates. Vertical axis is the absolute values of the contrasts standardised by the square root of pair age in millions of years (Myr) and plotted against the standardisation factor (Sqrt pair age). Shallow pairs with small substitution rates have higher variance because they are estimated from small numbers of observed substitutions. The shallowest pairs are successively removed until a linear model shows no evidence of a negative relationship (Wald test, $p > 0.05$). The data sets shown have undergone this process and have no detectable remaining relationship.

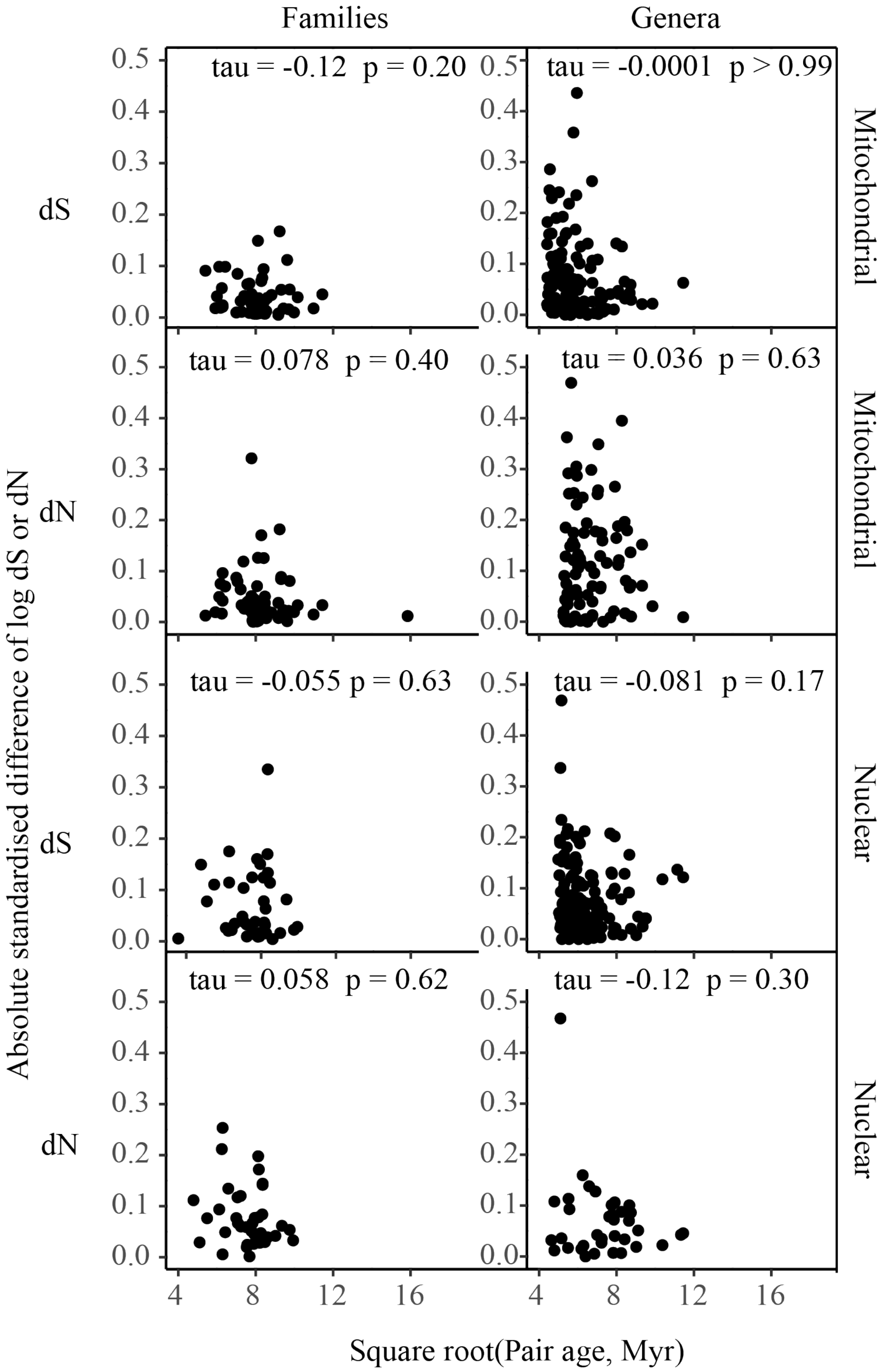


**Figure S23.** Tests for an effect of pair age on the variance of substitution rate estimates. Vertical axis is the absolute values of the contrasts standardised by the square root of pair age in millions of years (Myr) and plotted against the standardisation factor (Sqrt pair age). Non-synonymous (dN) or synonymous (dS) substitutions are inferred. Shallow pairs have higher variance because they are estimated from small numbers of observed substitutions. The shallowest pairs are successively removed until a linear model shows no evidence of a negative relationship (Wald test, $p > 0.05$). The data sets shown have undergone this process and have no detectable remaining relationship.

# 7. Whole Tree Analyses

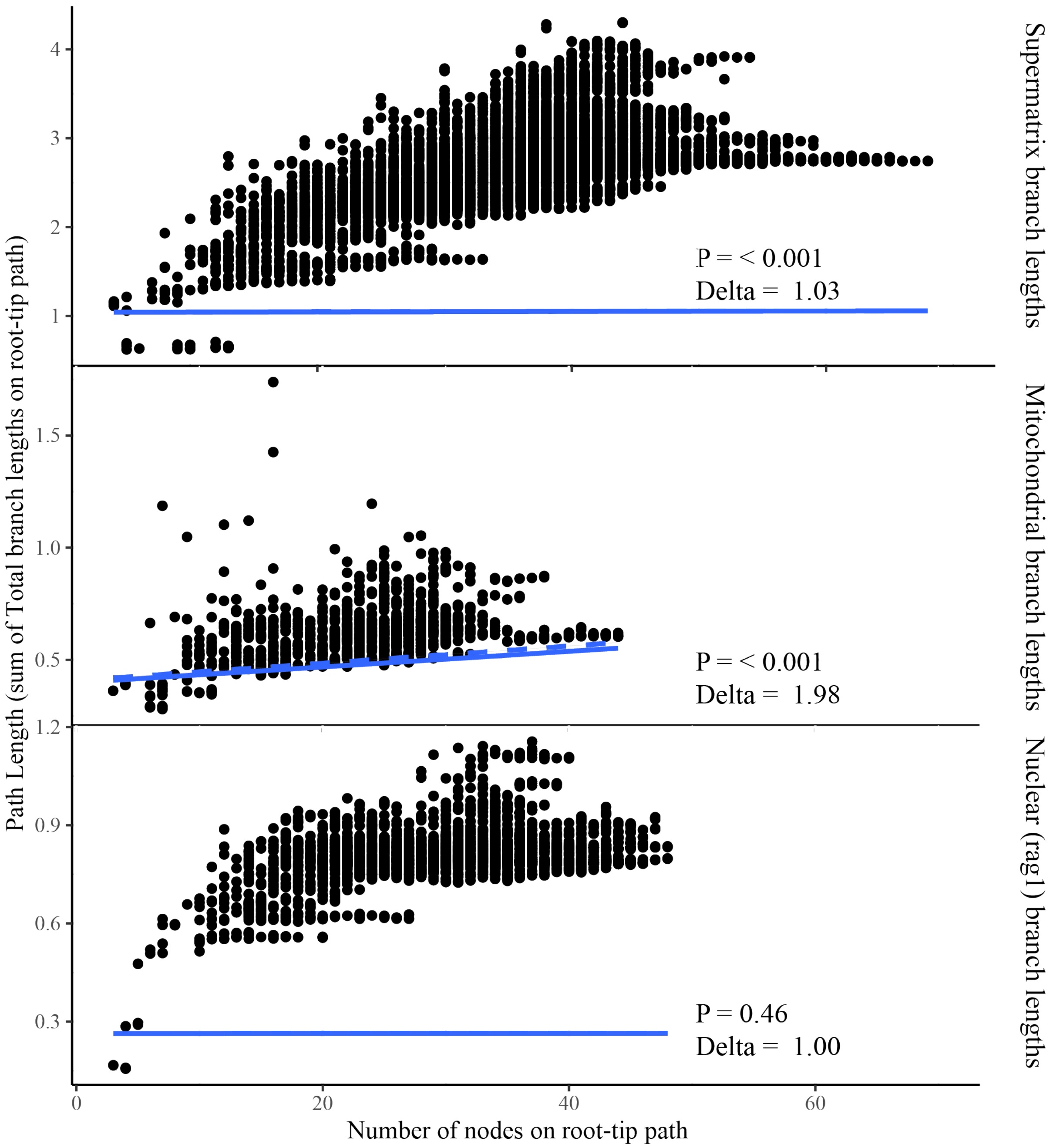


**Figure S24.** Tree-based analysis of the relationship between molecular substitutions and diversification by the method of Webster et al. (2003). The scatter plot shows the total estimated substitution rates on each root-to-tip path (the pathlength). The analysis is shown for the substitutions inferred from the full 11,638-sequence supermatrix from Rabosky et al. (2018), and the substitutions inferred from mitochondrial (1130 sequences) and nuclear (rag1 only, 3035 sequences) alignments assembled in this study. These pathlengths are plotted against the number of nodes on the root-to-tip path. The trend line shows the result of a phylogenetic linear regression of pathlengths against numbers of nodes on the path. The dashed line in the mitochondrial plot shows the fit of a three-parameter model allowing for non-linearity caused by the node density effect. The best-fitting model is concave with exponent approximately 1, indicating minimal contribution from a node-density artefact.

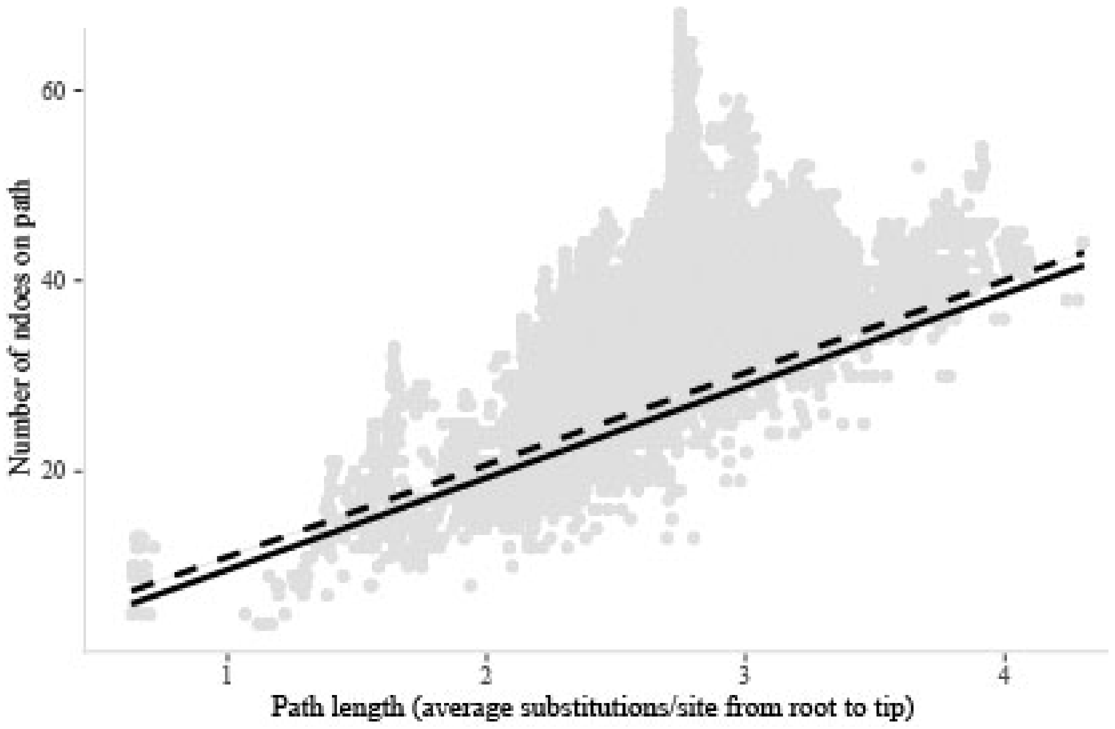


**Figure S25.** Axis – inverted tree-based analysis of the relationship between molecular substitutions and diversification by the method of Webster et al. (2003). Using the number of nodes as the response variable is the preferred method of Venditti et el. (2006) for diagnosing the node density effect, but was not usable for all of our tree-based analyses because the curve was fit to outlying points, producing trends with unreasonably large slopes. The scatter plot shows the total estimated total substitutions using mitochondrial data on each root-to-tip path in a 1133-tip tree from Rabosky et al. (2018). These pathlengths are plotted against the number of nodes on the root-to-tip path. The dashed trend line shows the result of a phylogenetic linear regression with a significant positive trend (likelihood ratio test; LR = 27.0 with 1 degree of freedom, $p < 10^{-6}$). The solid line shows the fit of a three-parameter model allowing for non-linearity caused by the node density effect. The best-fitting model is concave with exponent 0.95, indicating minimal contribution of the node-density effect.

# 8. Relationships among sister pairs

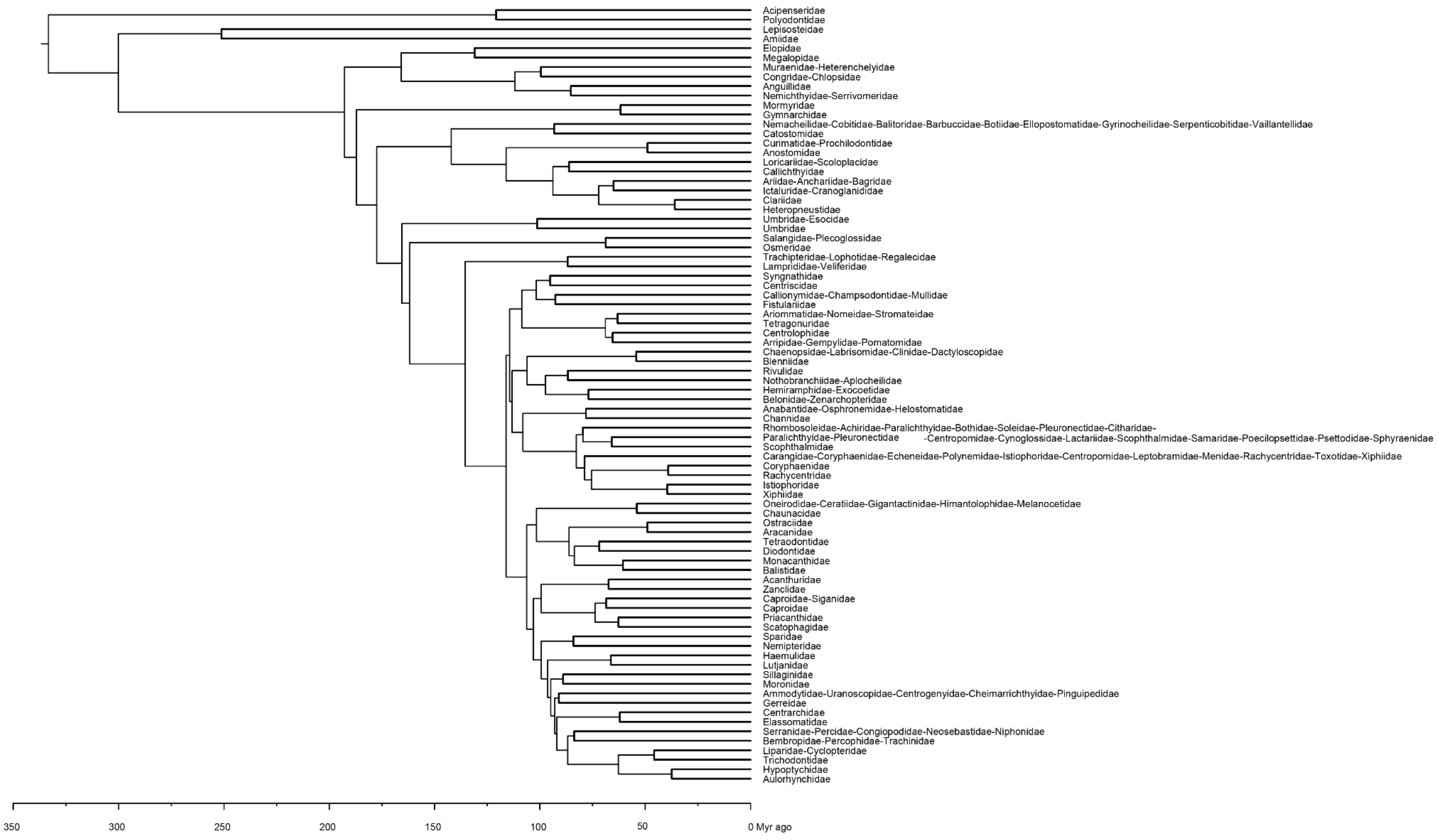

Acipenseridae
Polyodontidae
Lepisosteidae
Amiidae
Elopidae
Megalopidae
Muraenidae-Heterenchelyidae
Congridae-Chlopsidae
Anguillidae
Nemichthyidae-Serrivomeridae
Mormyridae
Gymnarchidae
Nemacheilidae-Cobitidae-Balitoridae-Barbuccidae-Botiidae-Ellopostomatidae-Gyrinocheilidae-Serpenticobitidae-Vaillantellidae
Catostomidae
Curimatidae-Prochilodontidae
Anostomidae
Loricariidae-Scoloplacidae
Callichthyidae
Ariidae-Anchariidae-Bagridae
Ictaluridae-Cranoglanididae
Clariidae
Heteropneustidae
Umbridae-Esocidae
Umbridae
Salangidae-Plecoglossidae
Osmeridae
Trachipteridae-Lophotidae-Regalecidae
Lampridae-Veliferidae
Syngnathidae
Centriscidae
Callionymidae-Champsodontidae-Mullidae
Fistulariidae
Ariommatidae-Nomeidae-Stromateidae
Tetragonuridae
Centrolophidae
Arripidae-Gempylidae-Pomatomidae
Chaenopsidae-Labrisomidae-Clinidae-Dactyloscopidae
Blenniidae
Rivulidae
Nothobranchiidae-Aplocheilidae
Hemiramphidae-Exocoetidae
Belonidae-Zenarchopteridae
Anabantidae-Osphronemidae-Helostomatidae
Channidae
Rhombosoleidae-Achiridae-Paralichthyidae-Bothidae-Soleidae-Pleuronectidae-Citharidae-
Paralichthyidae-Pleuronectidae
-Centropomidae-Cynoglossidae-Lactariidae-Scophthalmidae-Samaridae-Poecilopsettidae-Psettodidae-Sphyraenidae
Scophthalmidae
Carangidae-Coryphaenidae-Echeneidae-Polynemidae-Istiophoridae-Centropomidae-Leptobramidae-Menidae-Rachycentridae-Toxotidae-Xiphiidae
Coryphaenidae
Rachycentridae
Istiophoridae
Xiphiidae
Oneirodidae-Ceratiidae-Gigantactinidae-Himantolophidae-Melanocetidae
Chaunacidae
Ostraciidae
Aracanidae
Tetraodontidae
Diodontidae
Monacanthidae
Balistidae
Acanthuridae
Zanclidae
Caproidae-Siganidae
Caproidae
Priacanthidae
Scatophagidae
Sparidae
Nemipteridae
Haemulidae
Lutjanidae
Sillaginidae
Moronidae
Ammodytidae-Uranoscopidae-Centrogenyidae-Cheimarrichthyidae-Pinguipedidae
Gerreidae
Centrarchidae
Elassomatidae
Serranidae-Percidae-Congiopodidae-Neosebastidae-Niphonidae
Bembropidae-Percophidae-Trachinidae
Liparidae-Cyclopteridae
Trichodontidae
Hypoptychidae
Aulorhynchidae
350
300
250
200
150
100
50
0 Myr ago

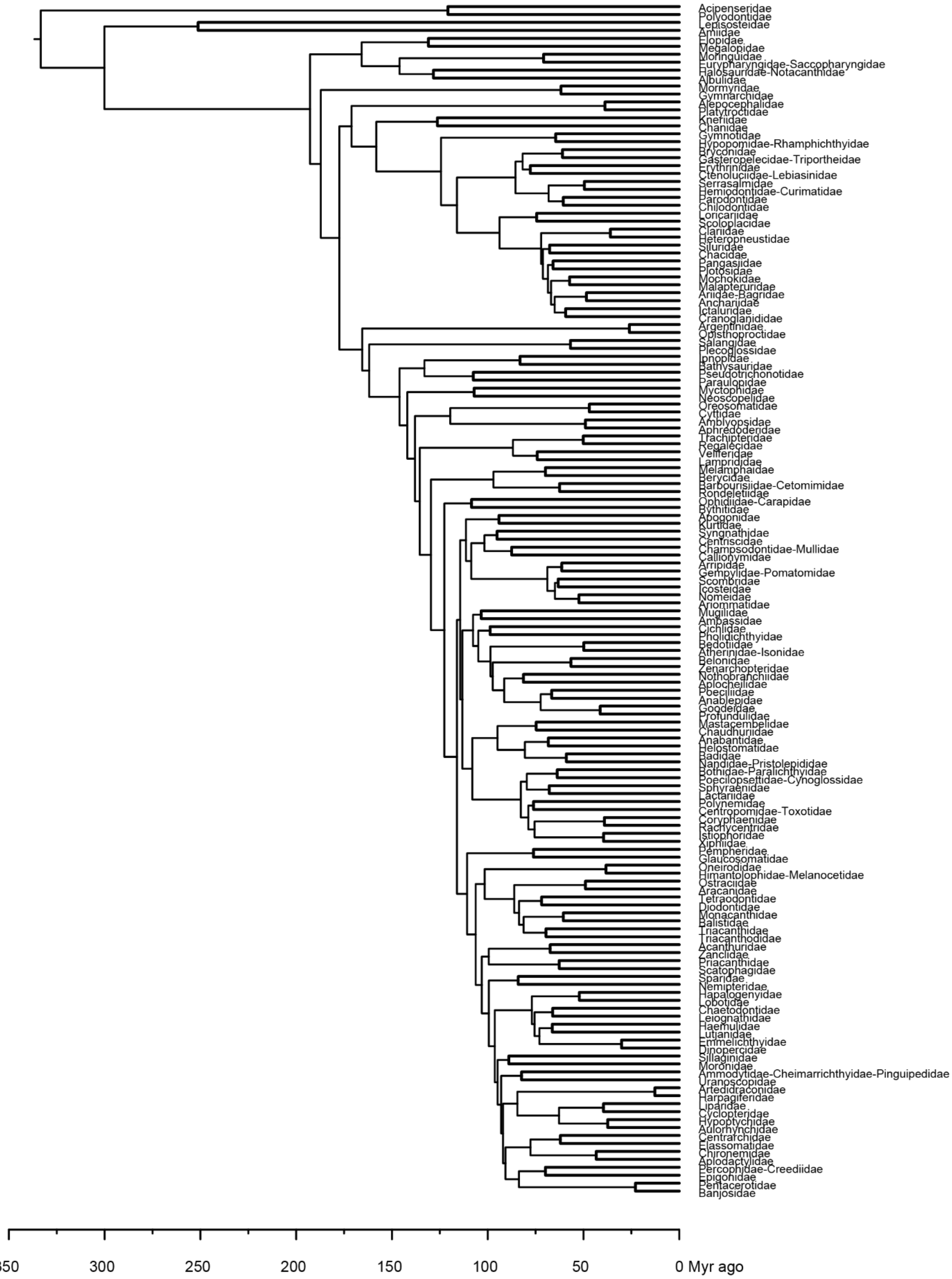


**Figure S27.** Visualisations of the set of family-level sister pairs for analysing nuclear total substitution rates. Thick lines show sister relationships. Tips not included in the analysis are not shown. Time is in millions of years ago. Some tips consist of clusters of families separated by dashes.

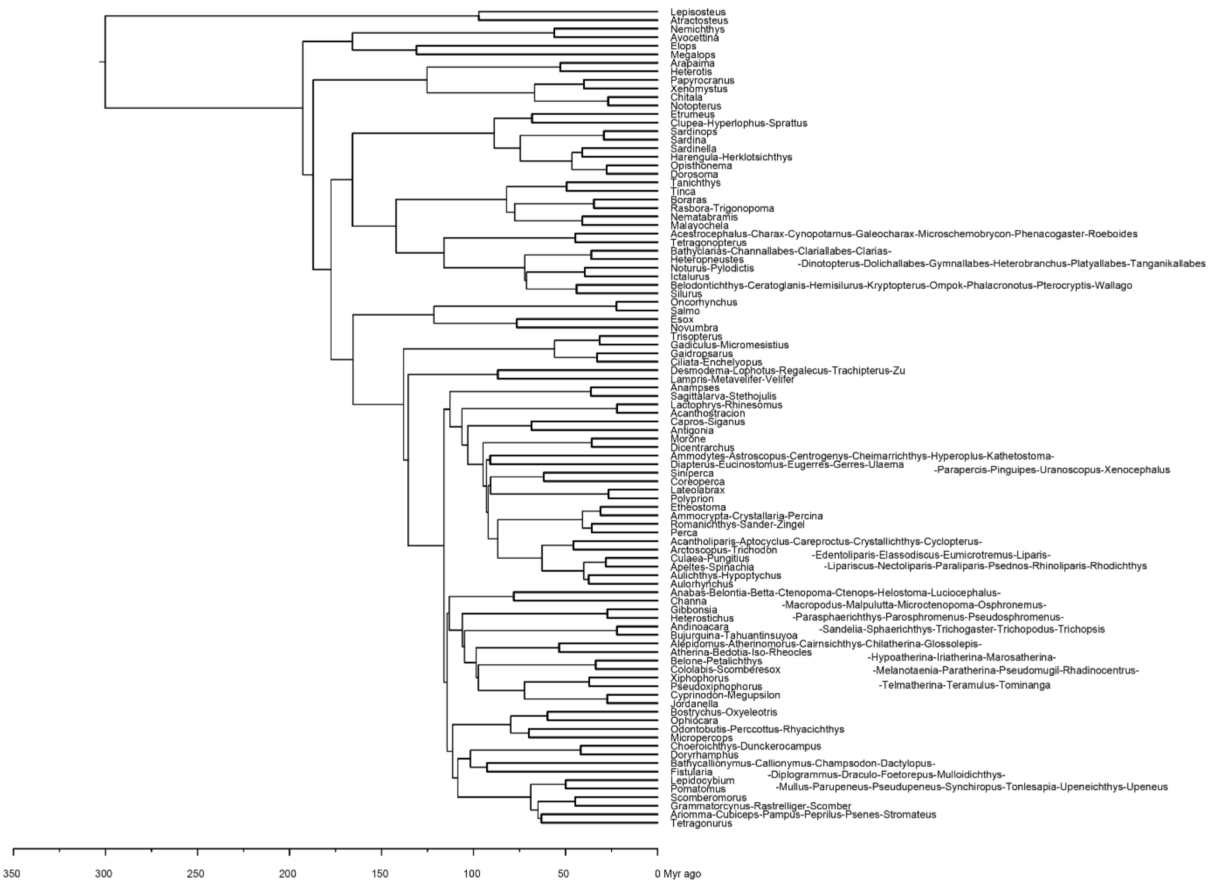


**Figure S28.** Visualisations of the set of genus-level sister pairs for analysing mitochondrial total substitution rates. Thick lines show sister relationships. Tips not included in the analysis are not shown. Time is in millions of years ago. Some tips consist of clusters of families separated by dashes.

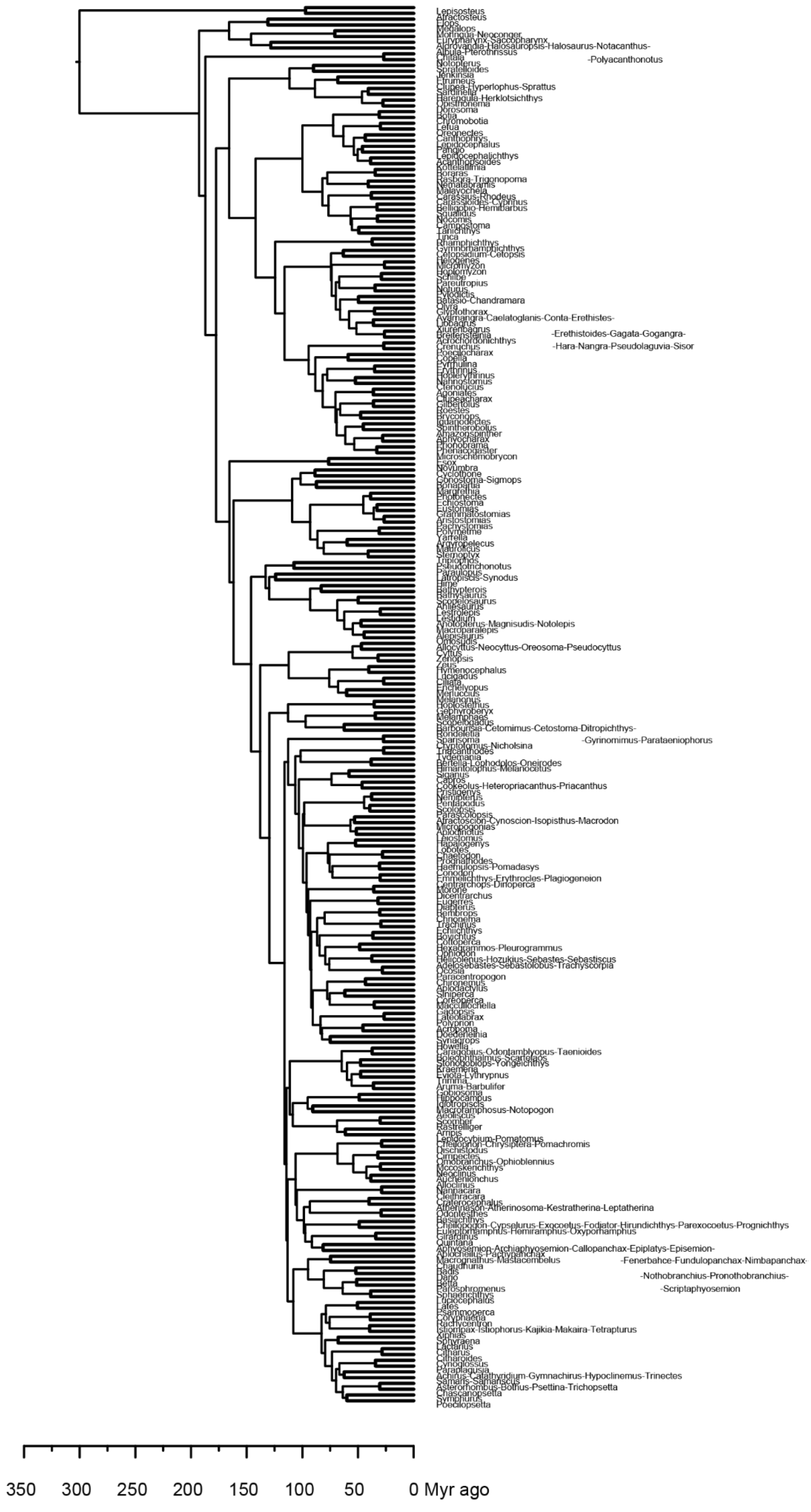


**Figure S29.** Visualisations of the set of genus-level sister pairs for analysing nuclear total substitution rates. Thick lines show sister relationships. Tips not included in the analysis are not shown. Time is in millions of years ago. Some tips consist of clusters of families separated by dashes.

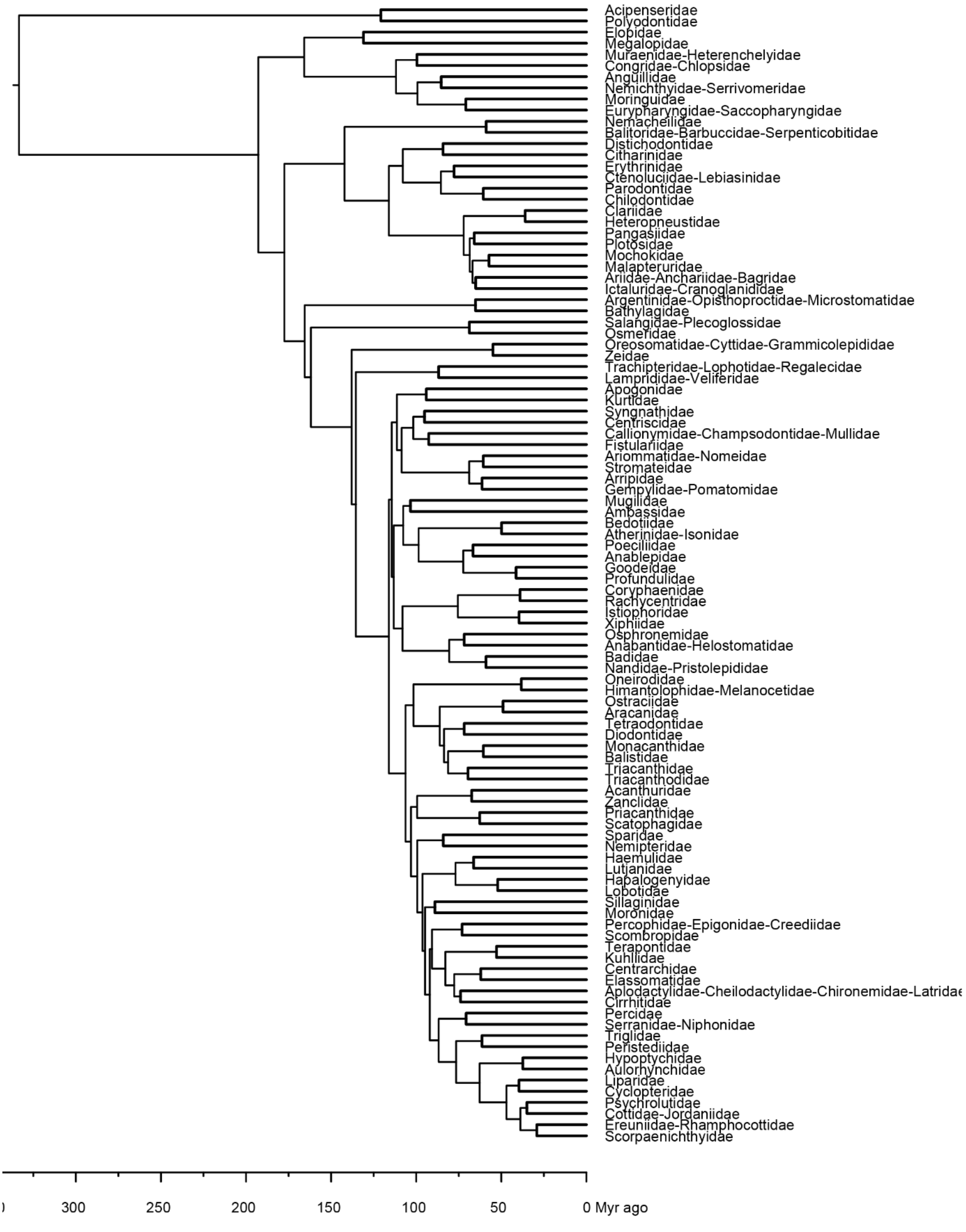
Acipenseridae
Polyodontidae
Elopidae
Megalopidae
Muraenidae-Heterenchelyidae
Congridae-Chlopsidae
Anguillidae
Nemichthyidae-Serrivomeridae
Moringuidae
Eurypharyngidae-Saccopharyngidae
Nemacheilidae
Balitoridae-Barbuccidae-Serpenticobitidae
Distichodontidae
Citharinidae
Erythrinidae
Ctenoluciidae-Lebiasinidae
Parodontidae
Chilodontidae
Clariidae
Heteropneustidae
Pangasiidae
Plotosidae
Mochokidae
Malapteruridae
Ariidae-Anchariidae-Bagridae
Ictaluridae-Cranoglanididae
Argentinidae-Opisthoproctidae-Microstomatidae
Bathylagidae
Salangidae-Plecoglossidae
Osmeridae
Oreosomatidae-Cyttidae-Grammicolepididae
Zeidae
Trachipteridae-Lophotidae-Regalecidae
Lamprididae-Veliferidae
Apogonidae
Kurtidae
Syngnathidae
Centriscidae
Callionymidae-Champsodontidae-Mullidae
Fistulariidae
Ariommatidae-Nomeidae
Stromateidae
Arripidae
Gempylidae-Pomatomidae
Mugilidae
Ambassidae
Bedotiidae
Atherinidae-Isonidae
Poeciliidae
Anablepidae
Goodeidae
Profundulidae
Coryphaenidae
Rachycentridae
Istiophoridae
Xiphiidae
Osphronemidae
Anabantidae-Helostomatidae
Badidae
Nandidae-Pristolepididae
Oneirodidae
Himantolophidae-Melanocetidae
Ostraciidae
Aracanidae
Tetraodontidae
Diodontidae
Monacanthidae
Balistidae
Triacanthidae
Triacanthodidae
Acanthuridae
Zanclidae
Priacanthidae
Scatophagidae
Sparidae
Nemipteridae
Haemulidae
Lutjanidae
Hapalogenyidae
Lobotidae
Sillaginidae
Moronidae
Percophidae-Epigonidae-Creediidae
Scombropidae
Terapontidae
Kuhliidae
Centrarchidae
Elassomatidae
Aplodactylidae-Cheilodactylidae-Chironemidae-Latridae
Cirrhitidae
Percidae
Serranidae-Niphonidae
Triglidae
Peristediidae
Hypoptychidae
Aulorhynchidae
Liparidae
Cyclopteridae
Psychrolutidae
Cottidae-Jordaniidae
Ereuniidae-Rhamphocottidae
Scorpaenichthyidae
300
250
200
150
100
50
0 Myr ago

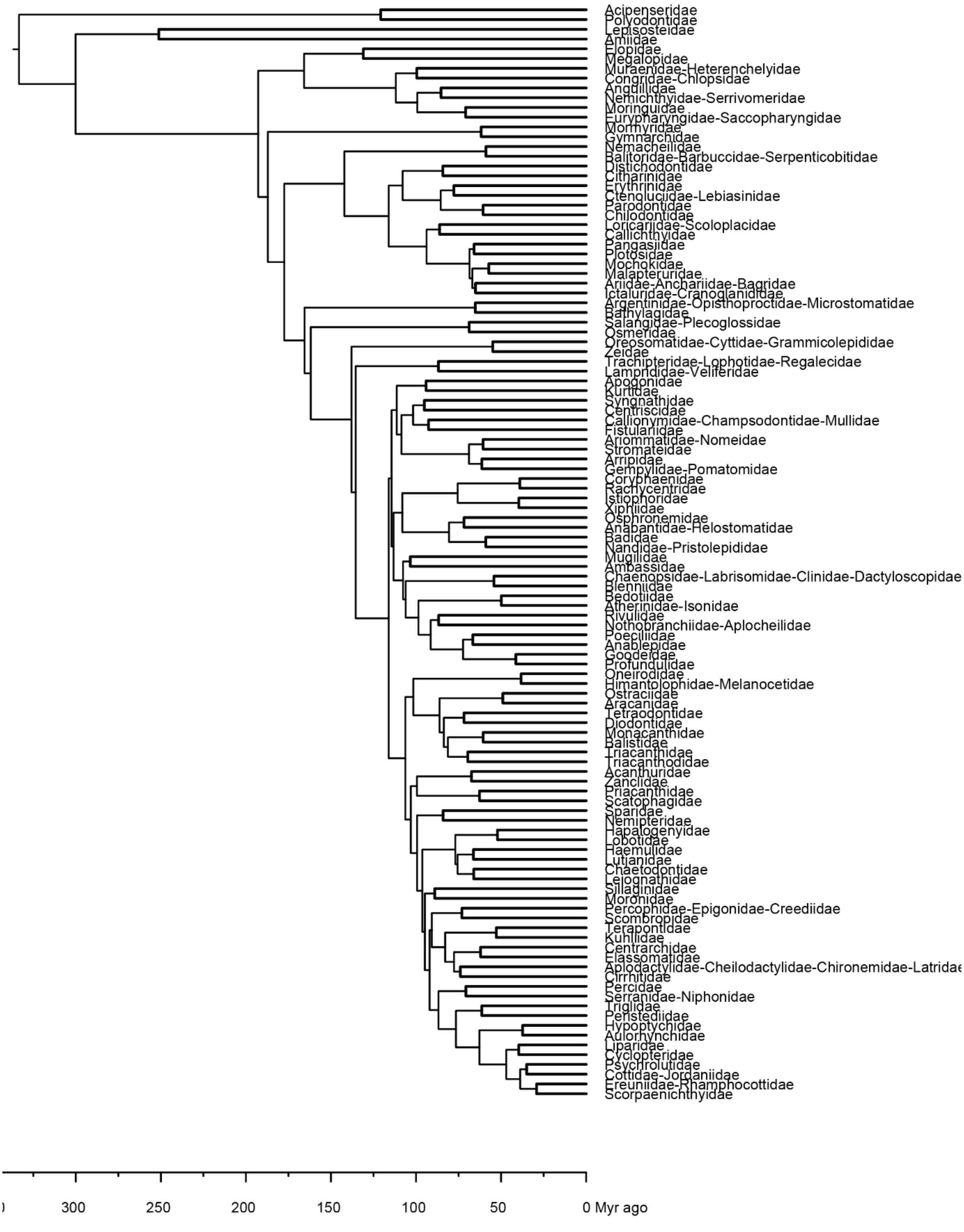


**Figure S31.** Visualisations of the set of family-level sister pairs for examining mitochondrial dN rates. Thick lines show sister relationships. Tips not included in the analysis are not shown. Time is in millions of years ago. Some tips consist of clusters of families separated by dashes.

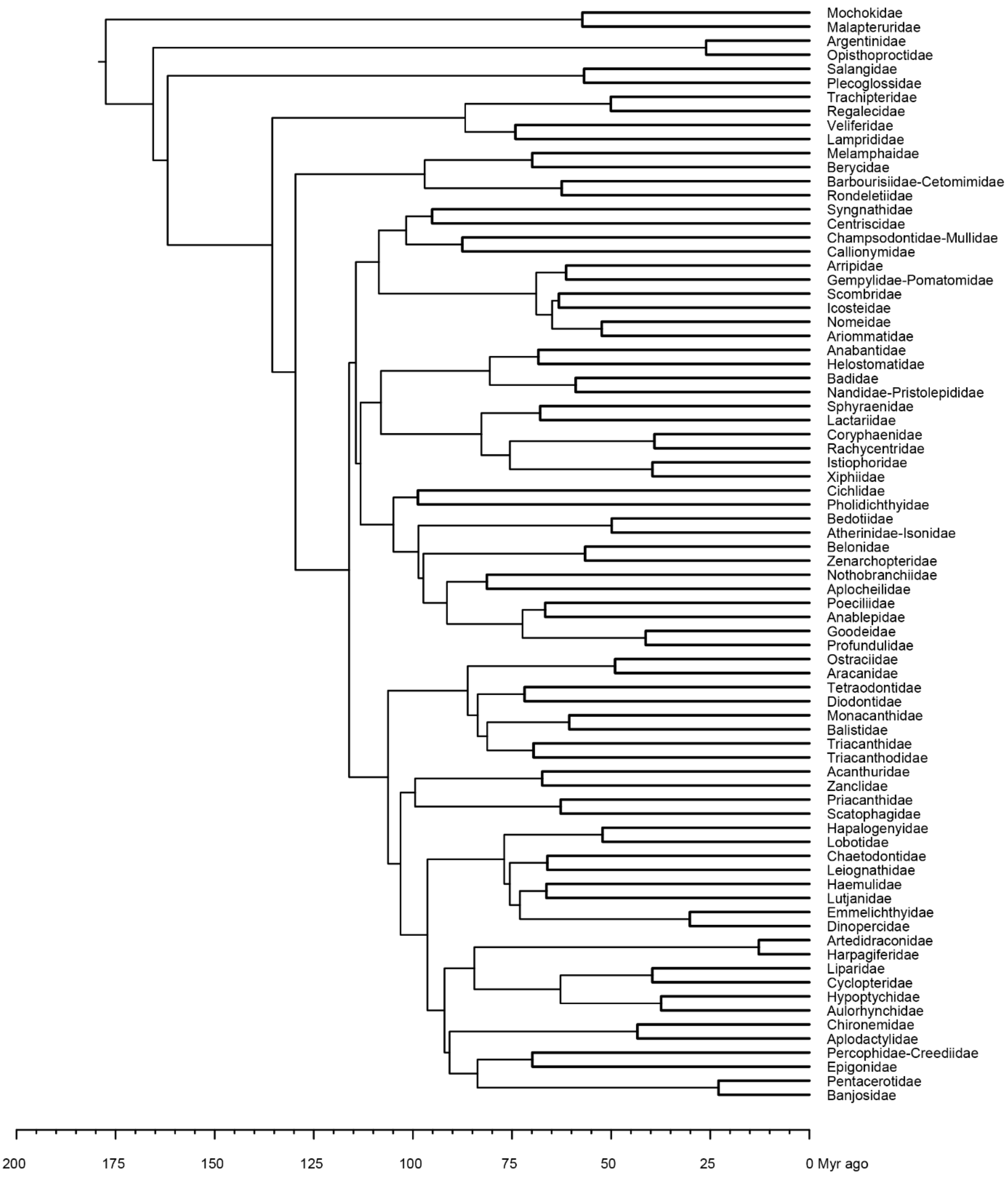


**Figure S32.** Visualisations of the set of family-level sister pairs for examining nuclear dS rates. Thick lines show sister relationships. Tips not included in the analysis are not shown. Time is in millions of years ago. Some tips consist of clusters of families separated by dashes.

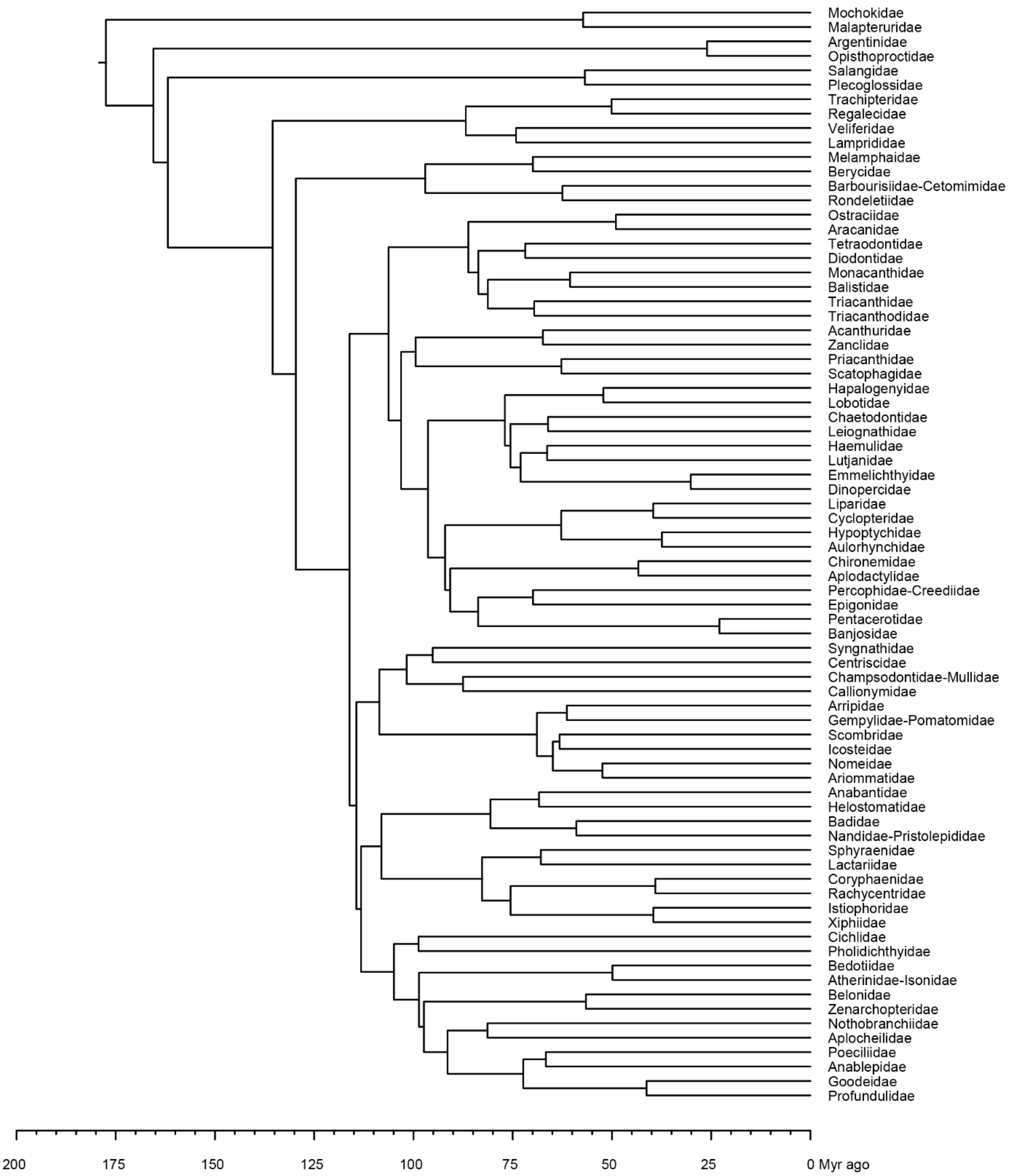


**Figure S33.** Visualisations of the set of family-level sister pairs for examining nuclear dN rates. Thick lines show sister relationships. Tips not included in the analysis are not shown. Time is in millions of years ago. Some tips consist of clusters of families separated by dashes.

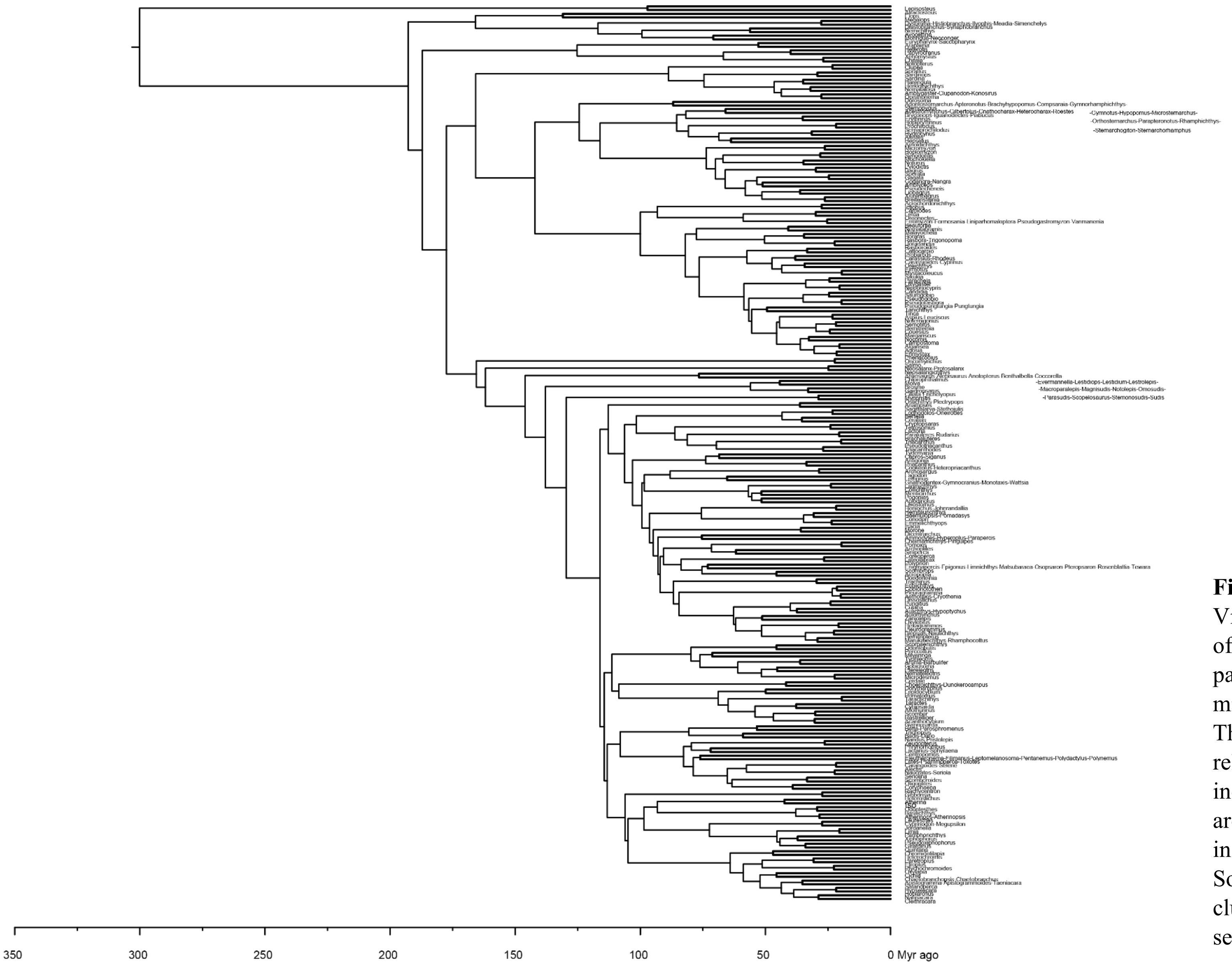


**Figure S34.** Visualisations of the set of genus - level sister pairs for examining mitochondrial dS rates. Thick lines show sister relationships. Tips not included in the analysis are not shown. Time is in millions of years ago. Some tips consist of clusters of families separated by dashes.

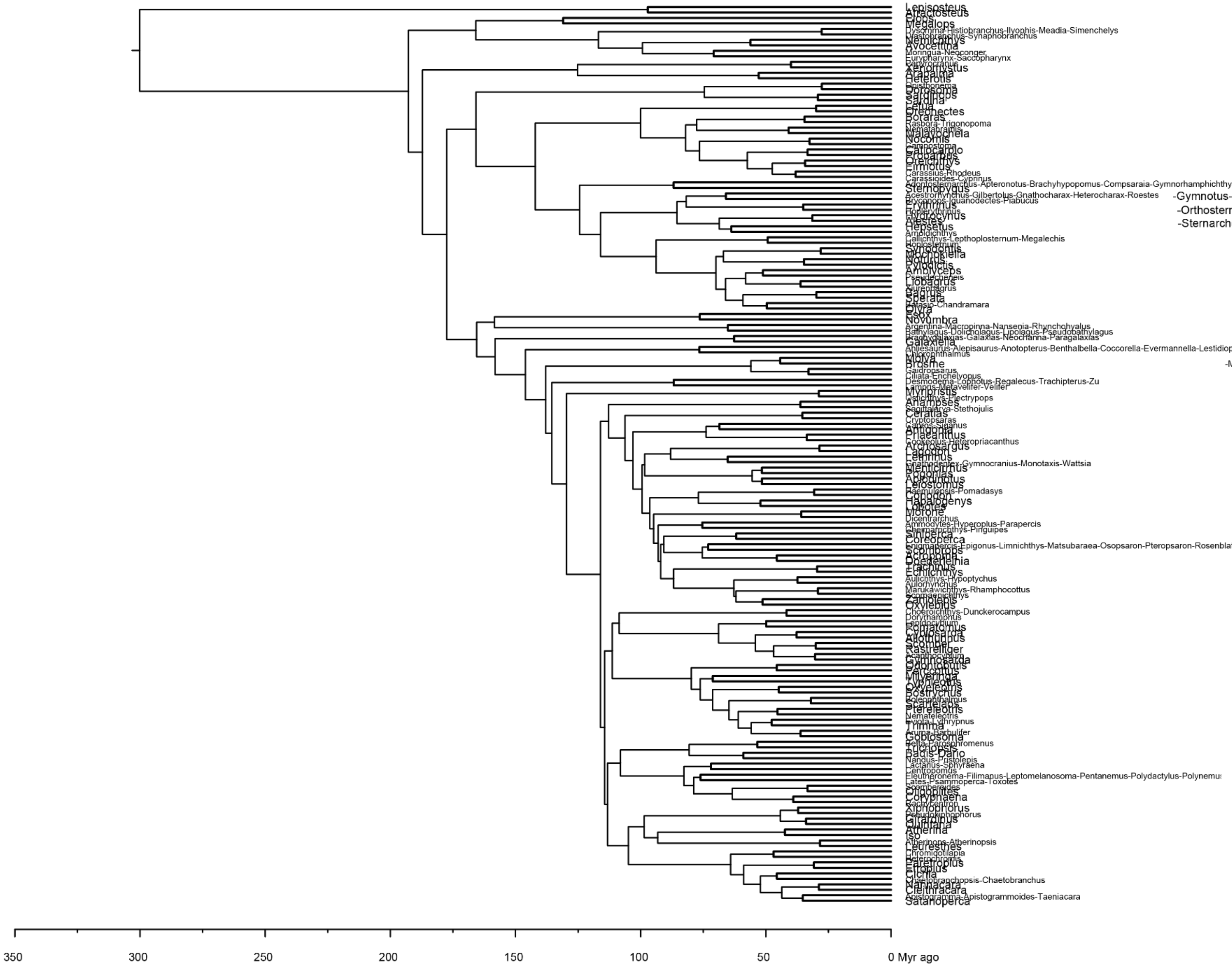


**Figure S35.** Visualisations of the set of genus - level sister pairs for examining mitochondrial dN rates. Thick lines show sister relationships. Tips not included in the analysis are not shown. Time is in millions of years ago. Some tips consist of clusters of families separated by dashes.

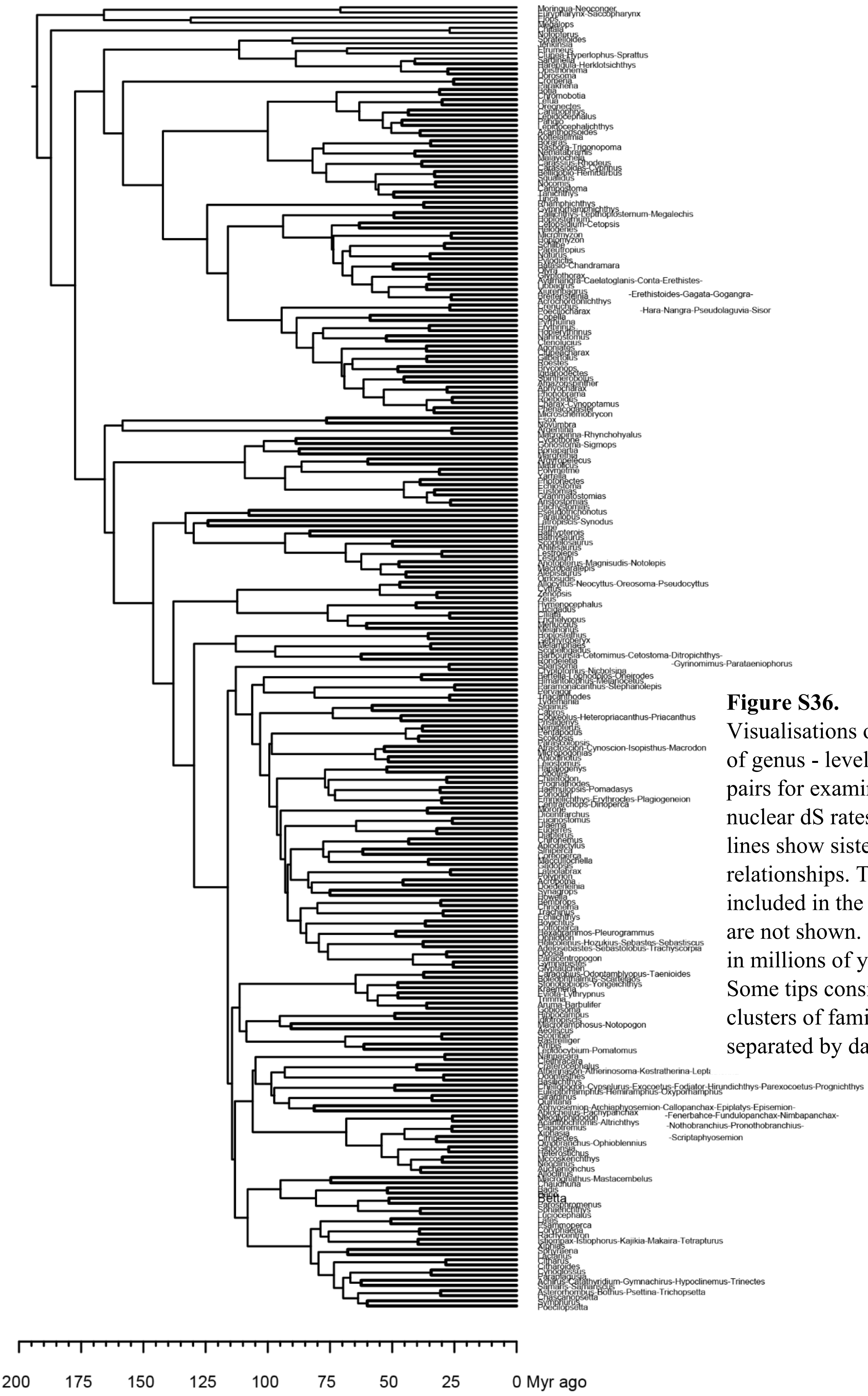


**Figure S36.** Visualisations of the set of genus - level sister pairs for examining nuclear dS rates. Thick lines show sister relationships. Tips not included in the analysis are not shown. Time is in millions of years ago. Some tips consist of clusters of families separated by dashes.

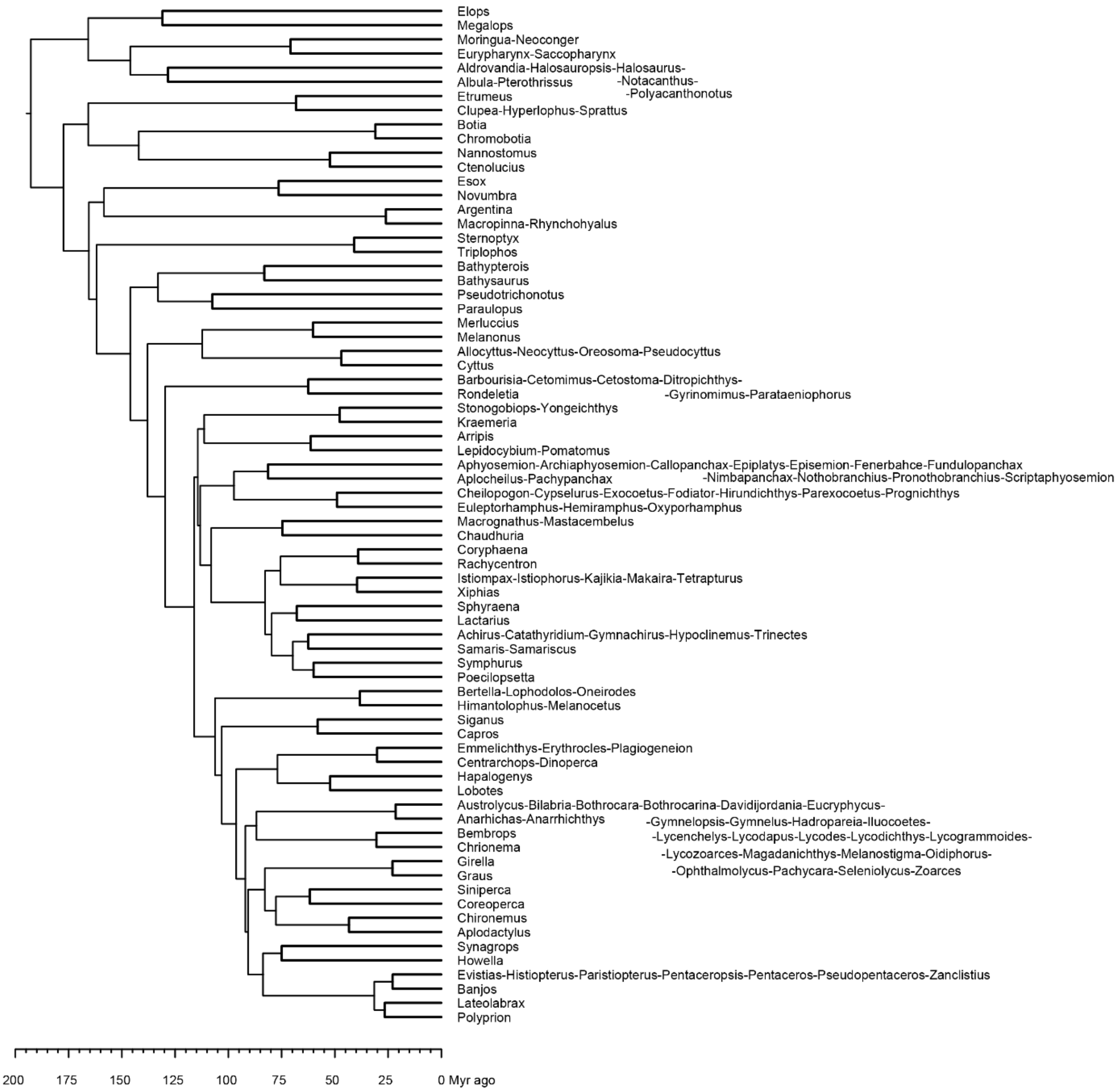


**Figure S37.** Visualisations of sister pairs on the consensus time-resolved phylogeny of Rabosky et al. (2018). The tree shows the set of genus-level sister pairs for examining nuclear dN rates. Thick lines show sister relationships. Tips not included in the analysis are not shown. Time is in millions of years ago. Some tips consist of clusters of families separated by dashes.
